# Purple *Brassica oleracea var. capitata F. rubra* is due to the loss of *BoMYBL2–1* expression

**DOI:** 10.1186/s12870-018-1290-9

**Published:** 2018-05-08

**Authors:** Hayoung Song, Hankuil Yi, Myungjin Lee, Ching-Tack Han, Jeongyeo Lee, HyeRan Kim, Jong-In Park, Ill-Sup Nou, Sun-Ju Kim, Yoonkang Hur

**Affiliations:** 1 Department of Biological Sciences, College of Biological Science and Biotechnology, Chungnam National University, Daejeon, 34134 Republic of Korea; 2 Department of Life Science, Sogang University, Seoul, 04107 Republic of Korea; 3 Korea Research Institute of Bioscience and Biotechnology, 125 Gwahangno, Yuseong-gu, Daejoen, 34141 Republic of Korea; 4 Department of Horticulture, Sunchon National University, Suncheon, Jeonnam 57922 Republic of Korea; 5 Department of BioEnvironmental Chemistry, College of Agriculture & Life Sciences, Chungnam National University, Daejeon, 34134 Republic of Korea

**Keywords:** Anthocyanin, *BoMYBL2–1*, Purple cabbage, Promoter substitution, Molecular marker

## Abstract

**Background:**

Water-soluble anthocyanin pigments are important ingredients in health-improving supplements and valuable for the food industry. Although great attention has been paid to the breeding and production of crops containing high levels of anthocyanin, genetic variation in red or purple cabbages (*Brassica oleracea var. capitata F. rubra*) has not yet been characterized at the molecular level. In this study, we identified the mechanism responsible for the establishment of purple color in cabbages.

**Results:**

*BoMYBL2–1* is one of the regulatory genes in the anthocyanin biosynthesis pathway in cabbages. It is a repressor whose expression is inversely correlated to anthocyanin synthesis and is not detectable in purple cabbages. Sequence analysis of purple cabbages revealed that most lacked *BoMYBL2–1* coding sequences, although a few had a substitution in the region of the promoter 347 bp upstream of the gene that was associated with an absence of *BoMYBL2–1* expression. Lack of transcriptional activity of the substitution-containing promoter was confirmed using transgenic *Arabidopsis* plants transformed with promoter::GUS fusion constructs. The finding that the defect in *BoMYBL2–1* expression was solely responsible for purple coloration in cabbages was further demonstrated using genomic PCR and RT-PCR analyses of many other structural and regulatory genes in anthocyanin biosynthesis. Molecular markers for purple cabbages were developed and validated using 69 cabbage lines.

**Conclusion:**

Expression of *BoMYBL2–1* was inversely correlated to anthocyanin content, and purple color in cabbages resulted from a loss of *BoMYBL2–1* expression, caused by either the promoter substitution or deletion of the gene. This is the first report of molecular markers that distinguish purple cabbages. Such markers will be useful for the production of intraspecific and interspecific hybrids for functional foods, and for industrial purposes requiring high anthocyanin content.

**Electronic supplementary material:**

The online version of this article (10.1186/s12870-018-1290-9) contains supplementary material, which is available to authorized users.

## Background

Anthocyanins synthesized by the flavonoid biosynthetic pathway are water-soluble pigments, responsible for pink, purple, red, and blue colors that are widely distributed in flowering plants [1–3]. Not only do anthocyanins attract animals for pollination and seed dispersal purposes, but they also protect plants from biotic and abiotic stresses, such as UV damage, cold stress, drought stress, and microbial agents [4–7]. Since stress resistance caused by anthocyanins mainly results from their strong antioxidant activity [8–12], anthocyanins are important ingredients in health improvement and prevents the onset of human metabolic syndromes; anthocyanins, for example, can improve visual function [13, 14], reduce the risk of cardiovascular disease [15–17], inhibit obesity and diabetes [9, 18], and exert anti-tumor effects by anti-inflammation and anti-cancer activity [19–21]. In addition to improving health and curing disease, anthocyanins are heavily used in the food industry [22], and thus an increase in anthocyanin content is one of the most important target traits in crop breeding.

Genes involved in anthocyanin biosynthesis can be divided into two groups: early biosynthesis genes (EBGs) and late biosynthetic genes (LBGs) [23, 24]. The EBG group consists of chalcone synthase (*CHS*), chalcone isomerase (*CHI*), flavanone 3-hydroxylase (*F3H*), flavanone 3′-hydroxylase (*F3’H*), and flavonol synthase (*FLS*) genes, which are regulated by three R2R3-MYB transcription factors (MYB11, MYB12, and MYB111) in the model plant *Arabidopsis* (*Arabidopsis thaliana*) [25–29]. The LBGs include dihydroflavonol 4-reductase (*DFR*), leucoanthocyanidin oxygenase (*LDOX*), anthocyanidin reductase (*ANR*), and UDP-glucose: flavonoid 3-*O*-glucosyltransferase (*UD3GT*), which is activated by the MYB-bHLH-WD40 (MBW) complex [23, 28–31].

The MBW complex is composed of R2R3-type MYB, bHLH042 (TT8), and bHLH042 (TRANSPARENT TESTA GLABRA 1; TTG1). Its formation is controlled by various environmental factors and repressors, including MYBL2 (MYB-LIKE 2) and SPL (SQUAMOSA PROMOTER BINDING PROTEIN-LIKE) [28, 29, 32]. MYBL2 is designated as one of CPC-like MYB-related genes originated from R2R3-MYBs [33], but it turns out to be a transcriptional repressor which contains two functional motifs, ethylene-responsive element binding factor-associated Amphiphilic Repression (EAR) motif [34] and TLLLER motif [24, 35–37]. It interacts with TT8 in the MBW complex, thereby inhibiting the expression of LBGs [24, 35]. In petunia, an EAR motif-containing R2R3-MYB, MyB27, represses transcription of both anthocyanin pathway genes and an essential component of MBW complex [38]. MYBL2, however, is a positive regulator of brassinosteroid (BR)-regulated plant growth during development; MYBL2 represses BR-repressed gene expression and is stabilized by phosphorylation by BRASSINOSTEROID INSENSITIVE 2 (BIN2), a GSK3-like kinase [39, 40]. Repression of *MYBL2* expression increases anthocyanin content in *Arabidopsis* [41, 42], but the role of *MYBL2* in anthocyanin biosynthesis in crop plants has not yet been reported.

In *Brassica* species, purple or red color by anthocyanin accumulation is closely related to the induction of anthocyanin biosynthetic genes and/or their transcription factors. Increases in expression of *TT8* and most anthocyanin biosynthetic genes are associated with purple coloration in pak choi (*Brassica rapa* var. *chinensis*) [40] and red mustard (*Brassica juncea* var. *tumida* Tsen et Lee) [39]. Similarly, accumulation of anthocyanin is caused by increases of *BoMYB2* and *bHLH-WD40* expression in purple cauliflower (*B. oleracea* L. var. *botrytis*) [44, 45]; of *BoMYB2* and *BoTT8* expression in red cabbage (*B. oleracea* var. *capitata*) [46]; of *B. oleracea PRODUCTION OF ANTHOCYANIN PIGMENTATION 1* (*BoPAP1/BoMYB2*) and downstream genes, such as *DFR* and anthocyanidin synthase (*ANS*), under the control of *BoPAP1* control in purple kale (*B. oleracea* var. *acephala F. tricolor*) [47]; and of most of the anthocyanin biosynthesis genes, as well as *BoPAP1* and *2* (*BoMYB4*), in kohlrabi [48]. Other purple or red crops derived by natural mutations in anthocyanin biosynthetic and regulatory genes have also been reported; for example, red apples resulting from a promoter rearrangement of *MdMYB10* [49, 50]; pink onions resulting from mutation in the *ANS* or *DFR* genes [51, 52]; beans with a black seed coat caused by deletion of the *CHS* promoter [53, 54]; purple cauliflower resulting from mutation in the *BoMYB2* promoter activating its expression [44]; and purple ornamental kale caused by the deletion of *DFR* [55]. Red or purple cabbage (*Brassica oleracea* L. var. *capitata* f, *rubra*), a crop native to the Mediterranean region of Europe, is now grown all over the world as a fresh market vegetable [46].

The molecular mechanism responsible for the establishment of heading-type purple cabbages is, however, largely unknown. We found that either the promoter substitution of *BoMYBL2–1* or deletion of the entire *BoMYBL2–1* gene resulted in the establishment of purple cabbages. Molecular markers to discriminate purple cabbage from green ones were developed based on sequence variation. These markers will be used to develop purple vegetable crops containing high levels of anthocyanin that will be valuable for health improvements and industry use.

## Methods

### Plant materials

Cabbages used for *BoMYBL2–1* cloning and marker validation were *B. oleracea* var. *capitata* f, *alba* or *rubra* plants from six green inbred lines, 32 green F_1_ cultivars, two purple inbred lines, two near-isogenic lines (NILs), 16 purple F_1_ cultivars, and 11 recombinants, as shown in Table 1. Inbred lines of green cabbages and purple cabbages, supplied by Asia Seed Co. (Korea), were selected to distinguish the genetic differences responsible for anthocyanin content.

**Table 1:** List of the *B. oleracea* lines and cultivars used in this study

Classification	Seed source	Name	Characteristics
Green cabbage	Asia Seed Co. (Inbred line)	337	High anthocyanin at low temperature
		154	..
		2437	Low anthocyanin at low temperature
		09WH-45	..
		2409	Green cabbage (no anthocyanin)
		842	..
	Botanical Interests Inc.	Green A	US cultivar (95–110 days)
	Lake Valley Seed	Green B	US cultivar (65 days)
	Asia Seed Co. (F1)	Daebakna	F1 hybrid
	Japan cultivar	NP-J-4 (Shoshu)	Takii seed (Extreme-early)
		NP-J-27 (YR danryu)	Masuda seed (Medium-early)
		NP-J-28 (YR Kinshukyouryoku 152)	Masuda seed (Medium-late)
		NP-J-31 (Saiho)	Takii seed (Early)
		NP-J-34 (YR Uijin)	Nakahara seed (Early)
		NP-J-51 (Kinryoku)	Yamatonoen seed (Early)
		NP-J-90 (Shoshudori)	Nosaki seed (Medium-early)
		NP-J-93 (Hideaki)	Nosaki seed (Early)
		NP-J-110 (Teruyoshi)	Norin seed (Medium)
		NP-J-117 (Shutoku SP)	Takii seed (Early)
		NP-J-135 (Koikaze)	Kaneko seed (Medium-early)
		NP-J-149 (Shogun)	Mikadokyowa seed (Extreme-early)
		NP-J-150 (Kagayaki)	Mikadokyowa seed (Extreme-early)
		NP-J-152 (YR Seinen)	Mikadokyowa seed (Early)
		NP-J-161 (YR Kiyomi)	Snow brand seed (Medium-early)
		NP-J-162 (YR Hatsumi)	Snow brand seed (Medium-early)
		NP-J-172 (YR Akiwase)	Masuda seed (Medium-early)
		NP-J-173 (Akiwase)	Masuda seed (Medium-early)
		NP-J-189 (YR Seitoku)	Takayama seed (Early)
		NP-J-191 (YR Yutoku)	Takayama seed (Medium)
		NP-J-198 (Best)	Watanabenoji (Early)
		NP-J-199 (Ajitama)	Watanabenoji (Early)
		NP-J-200 (Raien)	Watanabenoji (Early)
		NP-J-216 (YR Biminakawase)	Nohara seed (Early)
		NP-J-218 (Natsuboshi)	Nakahara seed (Early)
		NP-J-219 (Takara)	Tama seed (Early)
		NP-J-3 (Shikidori)	Takii seed (Late)
		NP-J-35 (Terunami)	Nakahara seed (Late)
		NP-J-36 (Harunami)	Nakahara seed (Late)
Purple cabbage	Asia Seed Co. (Inbred line)	7S4–63	Purple cabbage
		7S4–51	..
	HanKook Seed Co.	B90	NIL from J RED (Late)
	HanKook Seed Co.	B98	NIL from Primero (Early)
	Botanical Interests Inc.	Purple A	110–115 days, Red Acre
	Lake Valley Seed	Purple B	Red Acre
	Japan cultivar	NP-J-38 (Red ball)	Nakahara seed (Early)
		NP-J-126 (Power ruby)	Takii seed (Medium-early)
		NP-J-127 (Nakate ruby ball)	Takii seed (Medium-early)
		NP-J-64 (Red cabbage)	Watanabe seed (Early)
		NP-J-71 (Power acre)	Medium-early
	Takii Seed Co.	Junsaeng Rubia	Purple cabbage (Imported)
	SAKATA Seed Co.	Red Jewel	..
	SAKATA Seed Co.	Varna	..
	Bejo Seed Co.	Ranchero	..
	Bejo Seed Co.	Autoro	..
	Bejo Seed Co.	Integro	..
	Bejo Seed Co.	Primero	..
	Nick-Zwaan	Rondale	..
	HanKook Seed Co.	DP60	F1
		HK-014	NR (New Recombination)
		HK-016	BOX-23
		HK-017	HKB-035
		HK-018 N	NR (New Recombination)
		HK-020	HKB-034
		HK-032	HKB-048
		HK-034	HKB-059
		HK-047	NR (New Recombination)
		HK-058	NR (New Recombination)
		HK-069	NR (New Recombination)
		HK-075	NR (New Recombination)

Cabbages used for sequence analyses were as follows: inbred lines 337 and 154, selected for high anthocyanin content at low temperature; inbred lines 2437 and 09WH-45, selected for low anthocyanin content; inbred lines 2409 and 842, selected for no anthocyanin content; and the US cultivars Green A and B. The purple cabbages selected were the inbred lines 7S4–51 and 7S4–63, the NILs B90 and B98, and Purple A and B. Additional seeds not listed in Table 1 were purchased at local markets. Most plants were cultivated in a greenhouse at Chungnam National University, Daejeon, Korea, although some leaf samples were obtained from Korean Seed companies.

### Cloning of genomic DNA and sequence analysis for *BoMYBL2–1*

Genomic DNA was isolated from leaf samples using the DNeasy Plant Mini kit (QIAGEN Gmbh, Germany). The DNA sequences around *BoMYBL2–1* (Bol016164 = Bo6g112670) available in two databases (http://brassicadb.org/brad/ and http://plants.ensembl.org/Brassica_oleracea/Info/Index) did not match each other. Bo6g112680 of TO1000 [56] was annotated as Bol016161, Bol016162, and Bol016163 of O2–12 [57] (Additional file 1: Table S1). Therefore, DNA sequences from three cultivars, kale, broccoli, and cauliflower, were reanalyzed using four primer sets (Additional file 1: Table S1; Additional file 2: Figure S2A). After that, DNA sequences of heading-type cabbages (*B. oleracea* var. *capitate*) were cloned and analyzed.

Genomic PCR was performed under the following conditions: denaturation for 5 min at 94 °C, 30 cycles of amplification (30 s at 94 °C, 30 s at 58 °C, and 2–5 min at 72 °C), and a final extension period of 7 min at 72 °C. PCR products were purified using a LaboPass Gel Extraction kit (Cosmogenetech, Korea) and cloned into the TA-vector using the T&A cloning kit (RBC Bioscience Co., Taiwan). *Escherichia coli* (DH5α) cells were transformed with plasmid DNA carrying the desired insert. Plasmid DNA was purified using DNA-Spin (Intron Biotech. Inc., Korea) before sequencing. As cabbages might contain multiple *MYBL2–1* alleles, at least ten clones from each line were sequenced and analyzed. Any possible PCR and/or sequencing errors were eliminated by aligning independent sequences.

### Inverse PCR (iPCR)

To clone DNA sequences from purple cabbages that could not be amplified using any of the primer sets (Additional file 1: Table S1), inverse PCR (iPCR) was performed on *Bol010163* gene sequences using the Universal GenomeWalker™ 2.0 (Clonetech, USA). DNA from purple cabbages and control DNA from the kit were digested with *Dra*I, *Eco*RV, *Pvu*II, and *Stu*I for 16–18 h at 37 °C, and purified using the NuceloSpin Gel kit and PCR Clean-Up kit (Macherey-Nagel GmbH & Co, Germany). After ligation with the GenomeWalker adaptor, primary PCR was performed with a *Bol010163* gene-specific primer (5’-AGACGTTGATGAGATCAACGGTTGTGA), followed by secondary PCR with an adaptor primer (5’-CATCCAATAAAGGCGAGCAAGAAAGGA). PCR products were electrophoresed on 1% agarose gels, and the resulting bands were excised, purified, and cloned using the T&A cloning kit (RBC Bioscience Co., Taiwan).

Three sizes of DNA fragments were obtained: 700 bp from the *Pvu*II library, 2.1 kbp from the *Dra*I library, and 4.0 kbp from the *Stu*I library. To minimize sequence error, at least five clones from each library were sequenced and analyzed.

### RT-PCR

Leaf samples were collected from at least three individual plants, and total RNA was isolated from liquid nitrogen-ground samples using TRIzol reagent (Invitrogen, USA), and further purified using the NucleoSpin RNA Clean-up Kit (Macherey-Nagel GmbH & Co., Germany). Total RNA (1 μg) was treated with RQ1 RNase-free DNAase (Promega, USA), and cDNA was synthesized using the Ace-α kit with oligo(dT) primers (Toyobo, Japan). Complementary DNA was diluted 10-fold, and 1 μl of diluted cDNA was used in a 20 μl PCR mixture. RT-PCR primers are listed in Additional file 3: Table S2; primers for *ACTIN 2* (*BoACT2*) were used as a control. A standard PCR was performed with a 5 min denaturation at 94 °C, followed by 20–30 cycles of amplification (30 s at 94 °C, 30 s at 60 °C, and 30 s at 72 °C), and a final extension time of 7 min at 72 °C. PCR products were analyzed following electrophoresis through 1.2% agarose gels.

### Marker development and validation

To distinguish green and purple cabbages based on their *BoMYBL2–1* sequences, three primer sets were designed following sequence alignment using ClustalW2 (http://www.ebi.ac.uk/Tools/msa/clustalw2/) (Table 2). The PCR mix consisted of 10–30 ng of genomic DNA, 5 pmol of each forward and reverse primer, 10 pmol of *BoACT2* primers, and 1× HiPi Plus PCR premix buffer in 20 μl. To optimize PCR conditions for each primer pair (BoMYBL2–1w-F and BoMYBL2–1w-R; BoMYBL2–1v-F and BoMYBL2–1v-R; BoMYBL2–1sub-F and BoMYBL2–1sub-R), several annealing temperatures, extension times, and cycle numbers were tested and set. For the primer pair BoMYBL2–1w-F and BoMYBL2–1w-R, used to identify the presence of *BoMYBL2–1*, the PCR conditions were 5 min at 94 °C, followed by 28 cycles of 30 s at 94 °C, 30 s at 65 °C, and 2 min at 72 °C, with a final extension phase of 5 min at 72 °C. For the primer pair BoMYBL2–1v-F and BoMYBL2–1v-R, used to identify promoter-substituted *BoMYBL2–1*, the reaction conditions were 5 min at 94 °C, followed by 30 cycles of 30 s at 94 °C, 30 s at 60 °C, and 1 min at 72 °C, and a final extension of 5 min at 72 °C. For the primer pair BoMYBL2–1sub-F and BoMYBL2–1sub-R, used to identify DNA with a substitution of the whole *BoMYBL2–1* gene, the reaction conditions were 5 min at 94 °C, followed by 30 cycles of 30 s at 94 °C, 30 s at 65 °C, and 2 min at 72 °C, and a final extension of 5 min at 72 °C.

**Table 2:** Sequences of marker primers used to distinguish between green and purple cabbages

Use	Forward primer		Reverse primer	
	Name	Sequence	Name	Sequence
Wild-type * BoMYBL2–1* (947 bp) Variant *BoMYBL2–1* (1148 bp) Substituted *BoMYBL2–1* (2075 bp) *Actin 2* (144 bp)	BoMYBL2–1w-F	5’-CTACCAGTCTCTCCTTTGAAGAAGAC	BoMYBL2–1w-R	5’-GAGTTTTCCTTGATCTCACAGTACATTTCT
	BoMYBL2–1v-F	5’-TGTCCACTATCAACAAAAGATGATC	BoMYBL2–1v-R	5’-CAAACTCACCCGTGCAAATGTACAT
	BoMYBL2–1sub-F	5’-GTTCAAGCTCATTGTGAACGGA	BoMYBL2–1sub-R	5’-GGACCACCGTGAGAGAGAGA
	BoACT2-F	5’-TACGGTAACATCGTGCTCAGTG	BoACT2-R	5’-GATCCAGACACTGTACTTCCTC

### Presence of genes associated with anthocyanin biosynthesis

To examine whether additional genes were defective in purple cabbages, the presence of genes associated with anthocyanin biosynthesis was studied using genomic PCR. The genes selected were transcriptional activators and repressors (Additional file 3: Table S2). PCR conditions were 5 min at 94 °C, followed by 20–30 cycles of 30 s at 94 °C, 30 s at 60 °C, and 30 s at 72 °C, and a final extension time of 5 min at 72 °C.

### Promoter analysis

To confirm whether the substituted promoter found in purple cabbages retained promoter activity, the wild-type and substituted versions of the *BoMYBL2–1* promoter were fused to the *GUS* reporter gene. *Arabidopsis* plants were transformed with the reporter constructs, and examined for GUS activity and expression. The *MYBL2–1* promoter regions from green and purple *B. oleracea* (1926 bp upstream from the ATG start codon for green *B. oleracea* and 1804 bp upstream for purple *B. oleracea*) were amplified by PCR using the specific forward primers (5’-GATCAGGATCCAAGAACACATGAACTT for green cabbage and 5’-GATCAGGATCCAAGAACCAGTGTTC for purple cabbage) and the same reverse primer (5’-GTGAGCCATGGTACGAGAAGCA). These primers contain the *Bam*HI and *Nco*I restriction sites (underlined).

The amplified fragments were inserted into the T&A cloning vector (Real Biotech Co., Taiwan), and the presence of the *MYBL2–1* promoter sequence was confirmed by sequencing. The fragments were liberated by digestion with *Bam*HI and *Nco*I, and subcloned into the pCambina3301-GUS binary vector, digested with the same enzymes. The resulting constructs were transformed into *Arabidopsis* plants using the *Agrobacterium tumefaciens*-mediated floral dip procedure [58]. Transformed plants were selected using 0.1% BASTA herbicide, and their identity was confirmed by PCR analysis of genomic DNA. T_3_ homozygous lines containing a T-DNA insertion at a single locus, determined by a 3:1 ratio of segregation of basta-resistance: sensitivity, were selected for the assay of promoter activity.

*Arabidopsis thaliana* wild-type (Col-0) and transgenic plants were grown in a growth chamber under 16 h light/8 h dark photoperiods at 22 °C and a light intensity of 100 μmol m^− 2^ s^− 1^. For plate culture, seeds were surface-sterilized with 30% bleach containing 0.1% Triton X-100, stratified for 3 days at 4 °C, and plated onto solidified half-strength Murashige and Skoog (MS) medium, plus or minus 90 mM sucrose. Three plants were sampled for RT-PCR study of *GUS* expression, and another three plants were used to analyze GUS expression.

For GUS expression studies, plants were incubated in GUS staining solution (1 mM X-GlucA in 100 mM sodium phosphate, pH 7.0, containing 5 mM Na_2_EDTA, 0.5 mM potassium ferrocyanide, 0.5 mM potassium ferricyanide, and 0.1% Triton X-100) for 16 h at 37 °C. After staining, the plants were washed in 95% ethanol at room temperature until wild-type (Col-0) *Arabidopsis* appeared clear.

### Quantification of total anthocyanin content

Total anthocyanin content was determined in cabbage leaves using methanol containing 1% HCl [59]; three independent biological replicates from three individual plants were used in each experiment. The tissues were ground under liquid nitrogen, and the powder was resuspended in a 1.5 ml tube containing methanol (1% HCl) at room temperature and centrifuged at 14,000 rpm for 10 min at 4 °C. The absorbance of the supernatants was determined spectrophotometrically at 530 nm and 657 nm. Total anthocyanin content was quantified as

$$ \mathrm{Q}=\mathrm{Log}10\ \left(\mathrm{A}530\hbox{--} 0.25\times \mathrm{A}657\right)\times \mathrm{FW}\hbox{--} 1 $$

where Q = total anthocyanins; A530 = absorption at 530 nm; A657 = absorption at 657 nm; FW = fresh weight of tissues (g).

## Results

### Selection of *BoMYBL2–1* gene

**Fig. 1:**
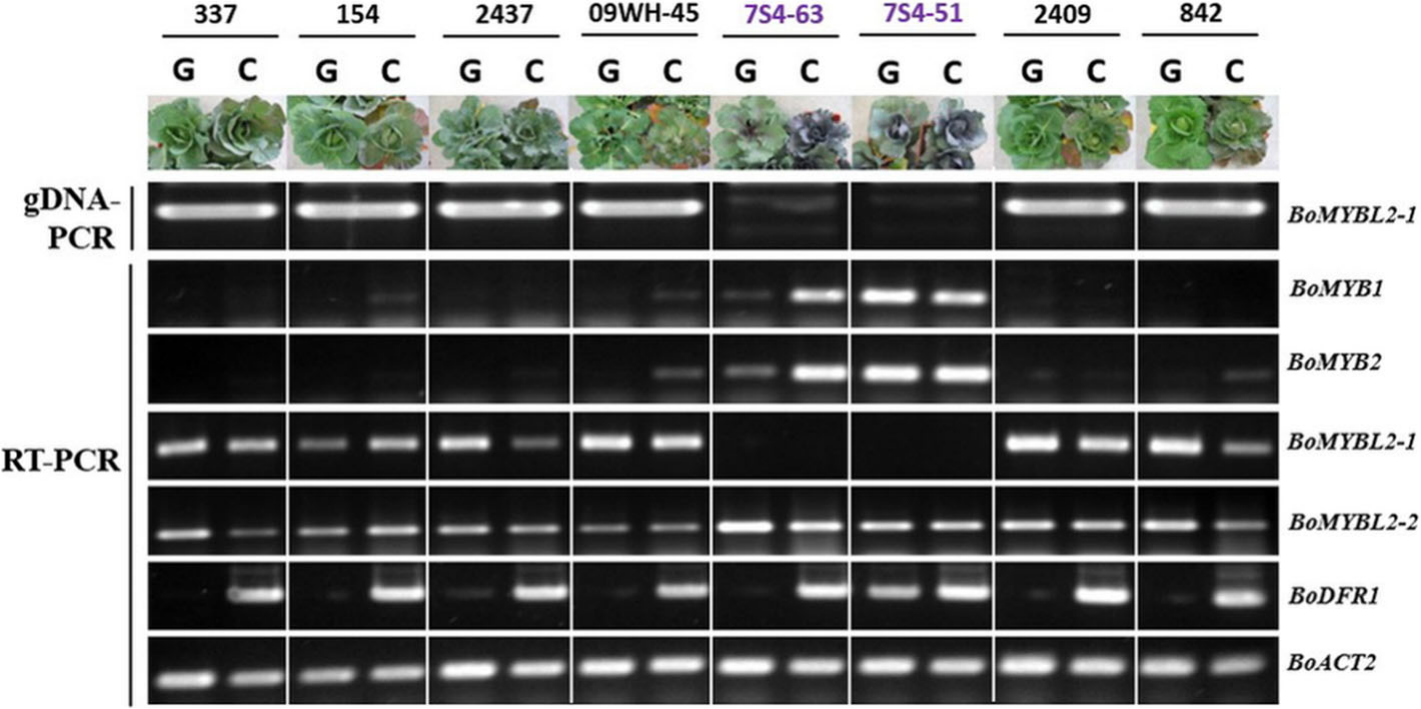
Analysis of anthocyanin biosynthesis-related genes from cabbage plants grown either in a greenhouse (G) or exposed to low temperature conditions outside (C). Plants were grown for 1 month between Oct. 13 and Nov. 12, 2014. Average temperatures ranged between 20 and 30 °C in the greenhouse and between 6 and 18 °C outside

To dissect the association of *BoMYBL2* with anthocyanin biosynthesis, expression of selected key genes in the pathway was examined in six inbred lines of green cabbage (337, 154, 2477, 09WH-45, 2409, and 842) and two inbred lines of purple cabbage (7S4–51 and 7S4–63). Two-month-old cabbage plants were grown for a month, either under greenhouse (G) conditions, which were non-inductive for anthocyanin accumulation, or outside (C), which was inductive for anthocyanin accumulation because it exposed the plants to low temperature (Fig. 1).

Expression of *BoMYB1* (*Bol042409* = *Bo3g081880*), *BoMYB2* (*Bol012528* = *Bo6g100940*), *BoDFR1* (*Bol035269* = *Bo9g058630*), *BoMYBL2–1* (*Bol01616*4 = *Bo6g112670*), and *BoMYBL2–2* (*Bol034966* = *Bo2g070770*) was analyzed. Two *BoMYBL2* genes (one on chromosome 6 and the other on chromosome 2) matched *Arabidopsis MYBL2* (*At1G71030*), and *BoMYBL2* designations were given according to the level of amino acid sequence identity with the *Arabidopsis* counterpart. As shown in Fig. 1, the expression of *BoDFR1* correlated with anthocyanin accumulation (Additional file 4: Figure S1). *BoMYB1* and *2* were highly expressed in purple cabbages, whereas *BoMYBL2–2* expression was detected in all samples. *BoMYBL2–1* expression was not detected in purple cabbages, and attempts to amplify the gene by PCR from the genomic DNA of purple cabbages failed, suggesting either a high level of sequence variation or deletion of *BoMYBL2–1*.

**Fig. 2:**
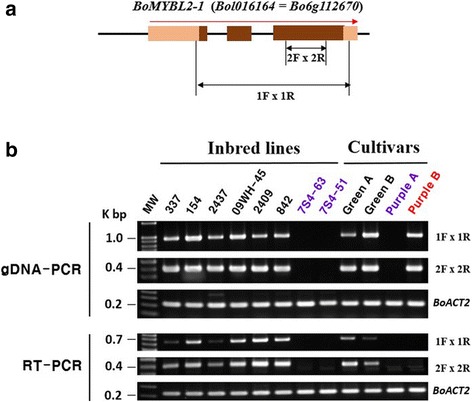
Presence and expression of *BoMYBL2–1* in various lines of cabbage. **a**: Genomic organization of *BoMYBL2–1* genes and positions of the primers used in PCR (1F, 2F, 2R, and 1R). **b**: Results of genomic DNA-PCR and RT-PCR

To investigate whether *BoMYBL2–1* had been deleted from the genome of purple cabbages, we examined the presence and expression of the gene in additional cultivars (Fig. 2). Both the *BoMYBL2–1* gene and its transcripts were detected in all the green cabbages tested. Two different types of result were obtained from two purple cabbages: (1) no amplification product from either genomic DNA or cDNA templates; and (2) amplification product from genomic DNA but not from cDNA. More specifically, the *BoMYBL2–1* transcript could not be detected in the purple B cultivar, although presence of the gene was confirmed from analysis of genomic DNA. These data suggest that two distinct mechanisms for the loss of *BoMYBL2–1* expression were responsible for the color of the two purple cabbages tested.

### Cloning and sequence analysis of *BoMYBL2–1* from various *B. oleracea* species

Genomic cloning of *BoMYBL2–1* was undertaken using six primer pairs (Additional file 2: Figure S2; Additional file 1: Table S1) and eight inbred lines of cabbages obtained from the Asia Seed Co. (Korea). An initial comparison of PCR results revealed that high levels of sequence variation were present around *BoMYBL2–1*, as observed in the distinct reference sequences previously obtained from two different varieties of *B. oleracea* [56, 57] (Additional file 1: Table S1). We therefore re-established a sequence assembly for kale (*B. oleracea* var. *sabellica*), broccoli (*B. oleracea* var. *italica*), and cauliflower (*B. oleracea* var. *botrytis*) cultivars using one allele from each variety (Additional file 5). Based on this new sequence information, sequencing and analysis were extended to inbred lines and cultivars of green cabbages, as well as purple cabbages (*B. oleracea* var. *capitata*) (Additional file 5). Nonetheless, no PCR product could be amplified from several lines of purple cabbages, including 7S4–51, 7S4–63, and B98. We used iPCR with primers designed around *Bol016163* to obtain sequence information for these plants.

**Fig. 3:**
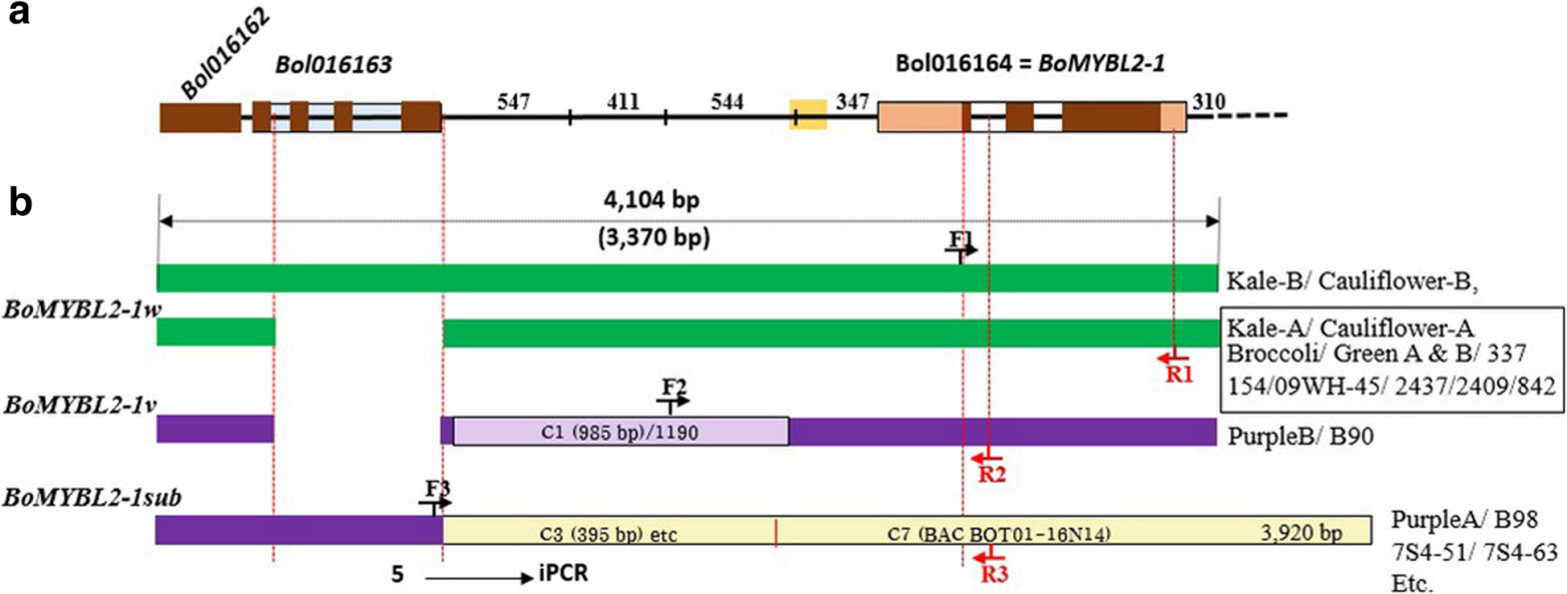
Schematic representation of *BoMYML2–1* genomic DNA from various *B. oleracea* plants showing the positions of the molecular markers. **a**: Schematic representation of the *BoMYBL2–1* genomic sequence based on the BRAD database (http://brassicadb.org/brad; Liu et al. 2014). The yellow block upstream of *BoMYBL2–1* represents a 159 bp repeat sequence. **b**: Schematic representation of *BoMYBL2–1* genomic DNA sequences identified in this study. iPCR indicates sequences obtained using inverse-PCR. *BoMYBL2–1w*, *BoMYBL2–1v*, and *BoMYBL2–1sub* represent the wild-type version, the *BoMYBL2–1* variant, and the *BoMYBL2–1* substitution, respectively. F1, F2, F3: forward primers 1, 2, 3; R1, R2, R3: reverse primers (see Table 2)

The simplified structure of the *BoMYBL2–1* genes and upstream nucleotide sequences from all of the lines used in our analysis (Additional file 5), which confirmed high levels of sequence variation in this region, are shown in Fig. 3. Kale and cauliflower were both heterozygous for *Bol016163* alleles, one with complete and the other with deleted versions, while broccoli appeared to have homozygous alleles. All heading-type cabbages and two purple cabbages (Purple B and B90) contained the deleted version of *Bol016163*; the other purple cabbages contained a complete version of *Bol016163*.

As shown in Fig. 3, all heading-type green cabbages carried *BoMYBL2–1* (1208 bp) plus approximately 1900 bp of upstream sequences. By contrast, two types of DNA sequence were observed in purple cabbages: *BoMYBL2–*1 with a substituted promoter containing 1190 bp of sequence, also found on chromosome 1, 347 bp upstream of the start codon (called *BoMYBL2–1* variant or *BoMYBL2–1v*), and a substitution (or deletion) type of the entire *BoMYBL2–*1 plus regulatory regions with putative chromosome 3 and chromosome 7 (called *BoMYBL2–1* substitution or *BoMYBL2–1sub*). Most purple cabbages contained *BoMYBL2–1sub*. To distinguish the wild-type or intact *BoMYBL2–1* sequence from *BoMYBL2–1v*, the wild-type version was designated *BoMYBL2–1w*. Interestingly, both *BoMYBL2–1w* and *BoMYBL2–1v* contained 159 bp repeat sequences upstream of the ATG start codon, which included seven ACCCGA repeats, 11 CGAA repeats, seven AAAT repeats, and so on. The MYB core motif GGATA [55] was detected in this repeat.

### Promoter analysis

**Fig. 4:**
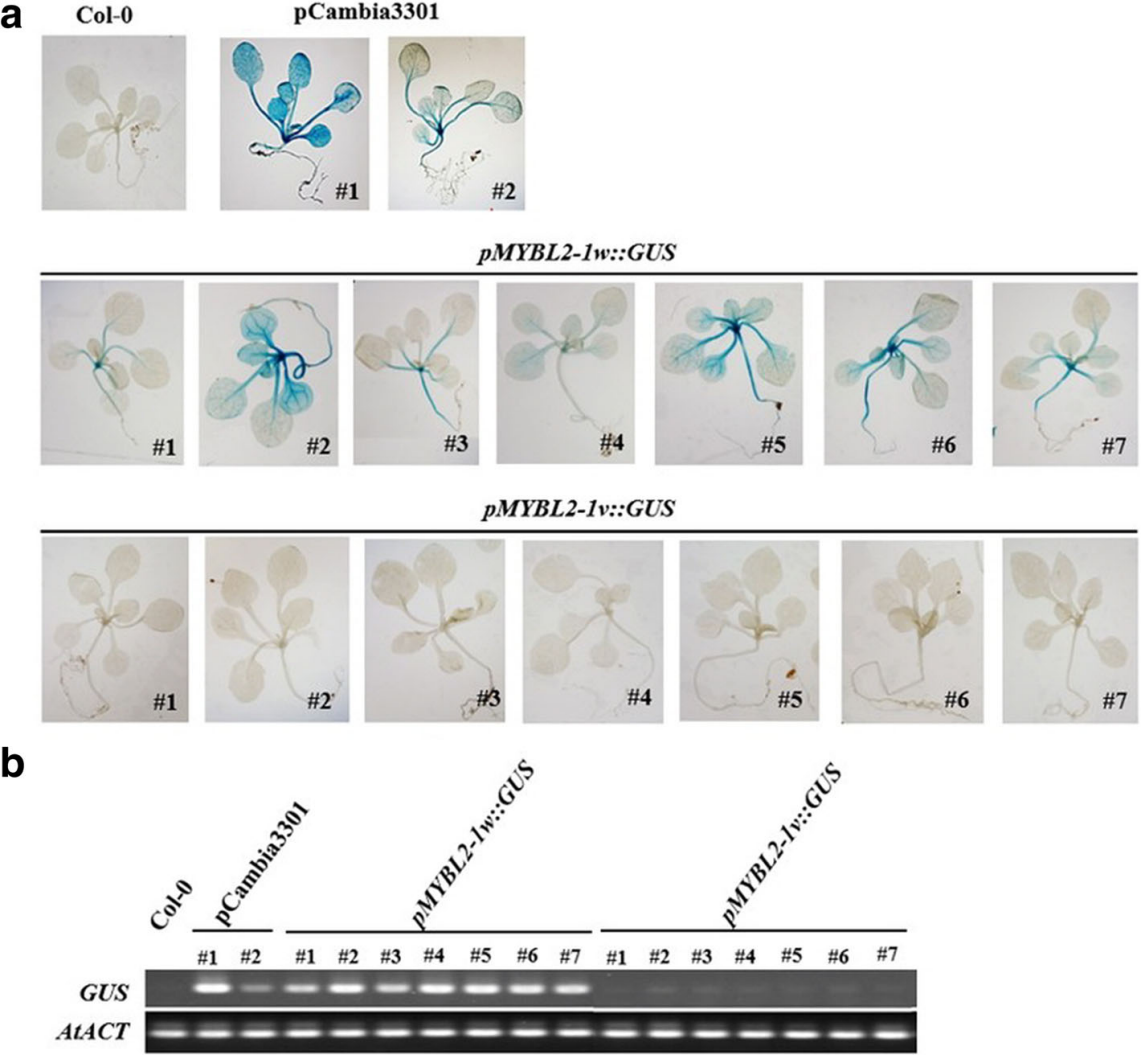
Expression analysis of *BoMYBL2–1* reporter constructs in transgenic *Arabidopsis*. *Arabidopsis* plants were transformed with reporter constructs containing *GUS* under the control of two different *BoMYBL2–1* promoters: *pMYBL2–1-w::GUS* and *pMYBL2–1v::GUS*. **a**: Histochemical analysis of 10-day-old transgenic *Arabidopsis* seedlings. Seedlings were stained for 18 h at 37 °C using X-Gluc (Sigma–Aldrich, St. Louis, MO, USA) and washed in 95% ethanol. Non-transgenic plants (Col-0) and pCambia3301 were used as negative and positive controls for GUS expression, respectively. **b**: Expression analysis of *GUS* transcripts in transgenic and control plants. *ACTIN* (*AtACT*) was used as a normalization control

**Fig. 5:**
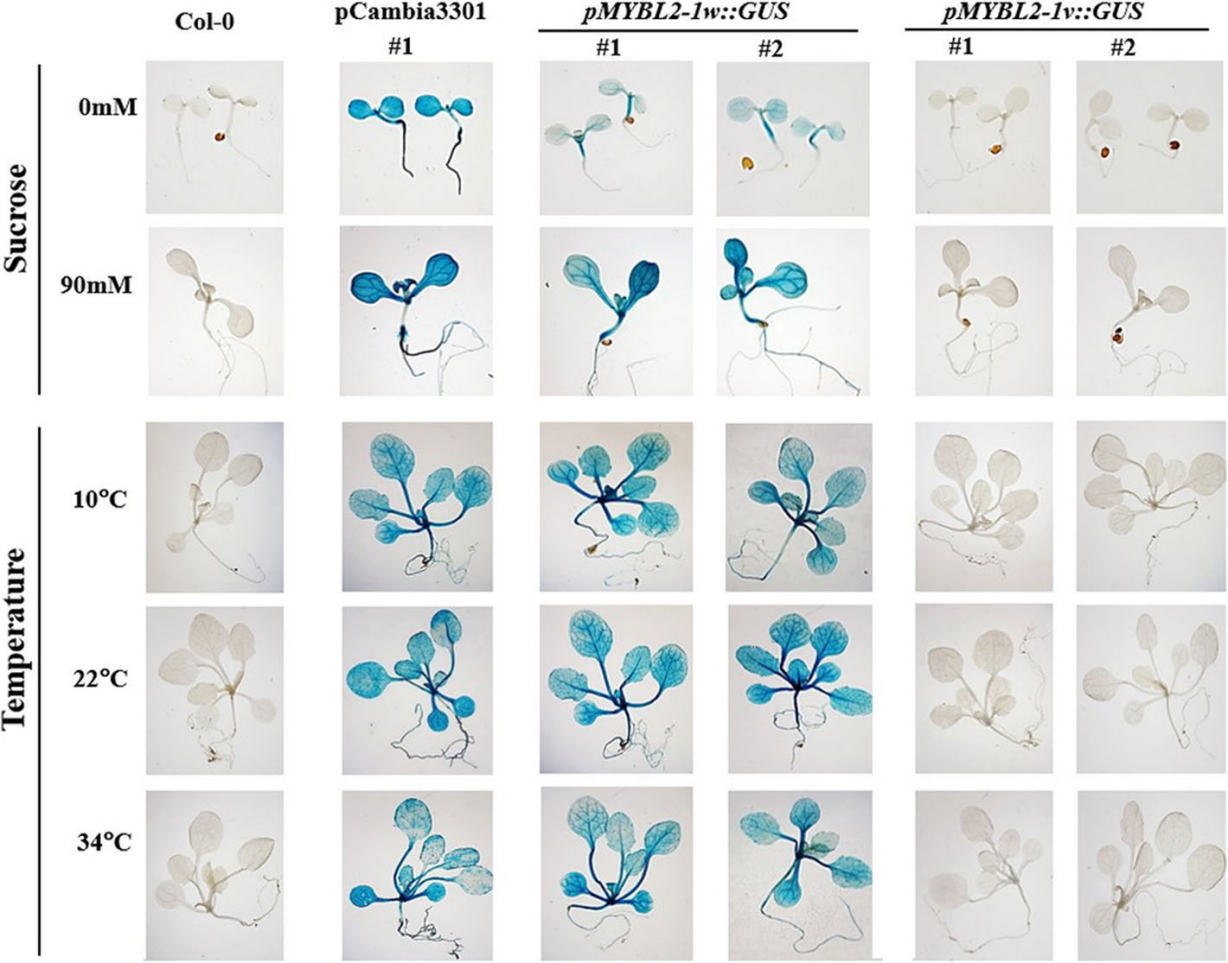
*GUS* expression in transgenic *Arabidopsis* under conditions of sucrose or temperature stress. For sucrose stress, Col-0 and mutant seedlings were grown in 0.5× MS medium without sucrose (0 mM) or with 90 mM sucrose. *Arabidopsis* seedlings were grown for 10 days after sowing at a light intensity of 100 μmol^− 2^ s^− 1^ under long-day conditions. For temperature stress, Col-0 and mutant plants were grown on soil for 12 days at 22 °C with light intensity of 100 μmol^− 2^ s^− 1^ under long-day conditions. They were transferred to incubators at either 10 °C (low temperature) or 34 °C (high temperature) with a light intensity of 100 μmol^− 2^ s^− 1^ and incubated for 19 h. GUS staining was as described in Fig. 4

To test whether the promoter region of *BoMYBL2–1v* had any transcriptional activity, 1926 bp from *BoMYBL2–1w* and 1804 bp from *BoMYBL2–1v* upstream of the ATG start codon were fused to the *GUS* reporter gene and the constructs were used to transform *Arabidopsis* plants. ß-glucuronidase (GUS) activity in *Arabidopsis* transgenic plants was evaluated using both histochemical staining and levels of expression of *GUS* transcript (Figs. 4 and 5). Two independent pCambia3301 transgenic plants were used as positive controls. Comparable levels of GUS staining (Fig. 4a) and *GUS* transcript expression (Fig. 4b) were shown by seven independent *pMYBL2–1w::GUS* transgenic plants. On the other hand, neither GUS staining nor *GUS* transcripts were detected in wild-type *Arabidopsis* (Col-0) or in seven independent *pMYBL2–1v::GUS* transgenic plants. These results indicated that the *pMYBL2–1v* promoter was non-functional under normal growth conditions.

**Fig. 6:**
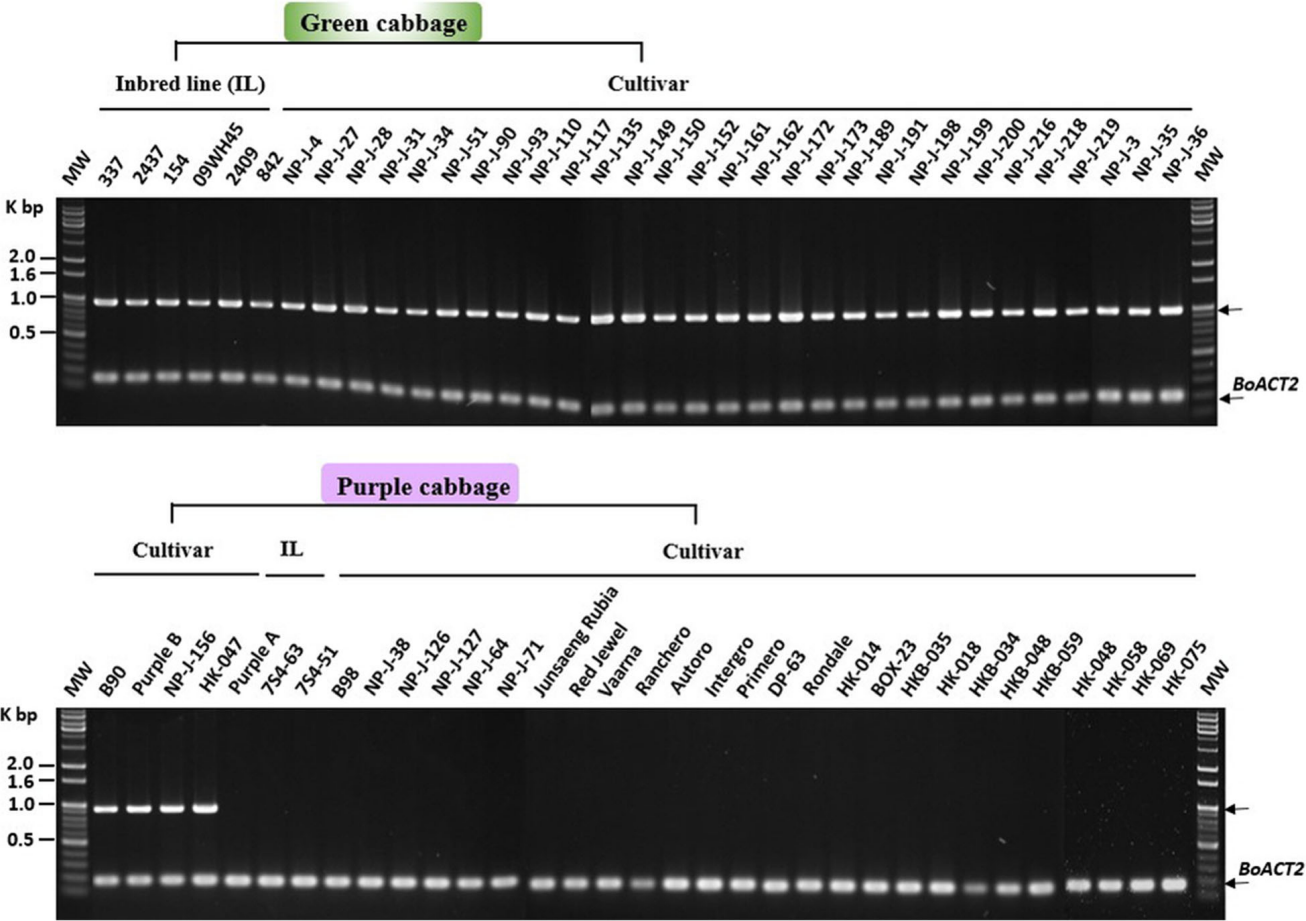
PCR analysis using the F1 and R1 primer pair to identify the presence of the complete coding region of *BoMYBL2–1*. Arrow indicates bands corresponding to *MYBL2–1*. Only four purple cabbages contain the coding region

Since anthocyanin biosynthesis is induced by various environmental factors, *GUS* expression in transgenic *Arabidopsis* plants was also tested under conditions of sucrose and temperature (low and high temperature) stress. As shown in Fig. 6, GUS staining was not detectible in *pMYBL2–1v::GUS* transgenic plants, even when the plants were subjected to the stress of 90 mM sucrose or exposure to high or low temperature. Taken together, these data strongly suggest that the promoter substitution found in *BoMYBL2–1v* resulted in the loss of promoter activity and *BoMYBL2–1* expression in some types of purple cabbages. In others, the change of color from green to purple may be explained by the complete deletion of the *BoMYBL2–1* coding sequence*.*

### Confirmation of a unique deletion of *BoMYBL2–1* in purple cabbages

To determine whether the color change to purple in *B. oleracea* var. *capitata* f *rubra* is attributable to the loss of other important genes regulating anthocyanin biosynthesis, we investigated the presence of regulatory genes, including *BoMYBL2–1*, associated with anthocyanin biosynthesis. *BoMYBD*, *BoSPL9*, *BoMYBH*, and *BoMYBL2–1* are negative regulators of anthocyanin biosynthesis, while *BoMYB1–4*, *BoMYB11*, and *BoMYB12* are transcriptional activators. *BoANL* (*Bo9g002480/Bol011495*) is a homeobox-leucine zipper gene equivalent to *ANTHOCYANINLESS 2* (*AT4G00730*).

As shown in Additional file 6: Figure S3, all these genes other than *BoMYBL2–1*, which was lost in several lines of purple cabbage, were present in various varieties of *B. oleracea*. This strongly suggested that, at least for some purple cabbages (*B. oleracea* var. *capitata* f, *rubra*), the deletion of *BoMYBL2–1* was responsible for the development of purple coloration.

Since the presence of a gene does not guarantee its expression, expression of key biosynthetic (*BoCHS1*, *BoCHS2*, *BoF3H*, *BoF3’H*, and *BoDFR1*) and regulatory genes affecting anthocyanin accumulation was examined in 20 different purple cabbage (Additional file 6: Figure S3). Transcript levels of most genes did not differ between these purple cabbages, but expression levels of several genes (*BoCHS1*, *BoF3H*, *BoDFR1*, and *BoMYB2*) were higher in those plants than in the green cabbage Daebakna. All the genes tested, other than *BoMYB4*, were expressed at relatively high levels, possibly as a result of defective BoMYBL2–1 activity.

### And validation of molecular markers to distinguish purple cabbage

Markers that are associated with a particular horticultural trait, such as purple coloration, are very important in developing new *B. oleracea* varieties with the desired trait from crosses of different subspecies. To develop molecular markers able to identify purple *B. oleracea* var. *capitata* carrying defective *BoMYBL2–1*, we designed three sets of primer pairs: (1) to amplify the *BoMYBL2–1* coding sequence; (2) to amplify the promoter-substituted variant *BoMYBL2–1* (*MYBL2v*); and (3) to amplify the DNA sequence that replaced the entire *BoMYBL2–1* gene (*BoMYBL2–1sub*) (Table 2; Fig. 3). The optimal PCR conditions for each pair are described above (Methods). The validity of these primer pairs was tested using the 35 green and 33 purple cabbages listed in Table 1.

**Fig. 7:**
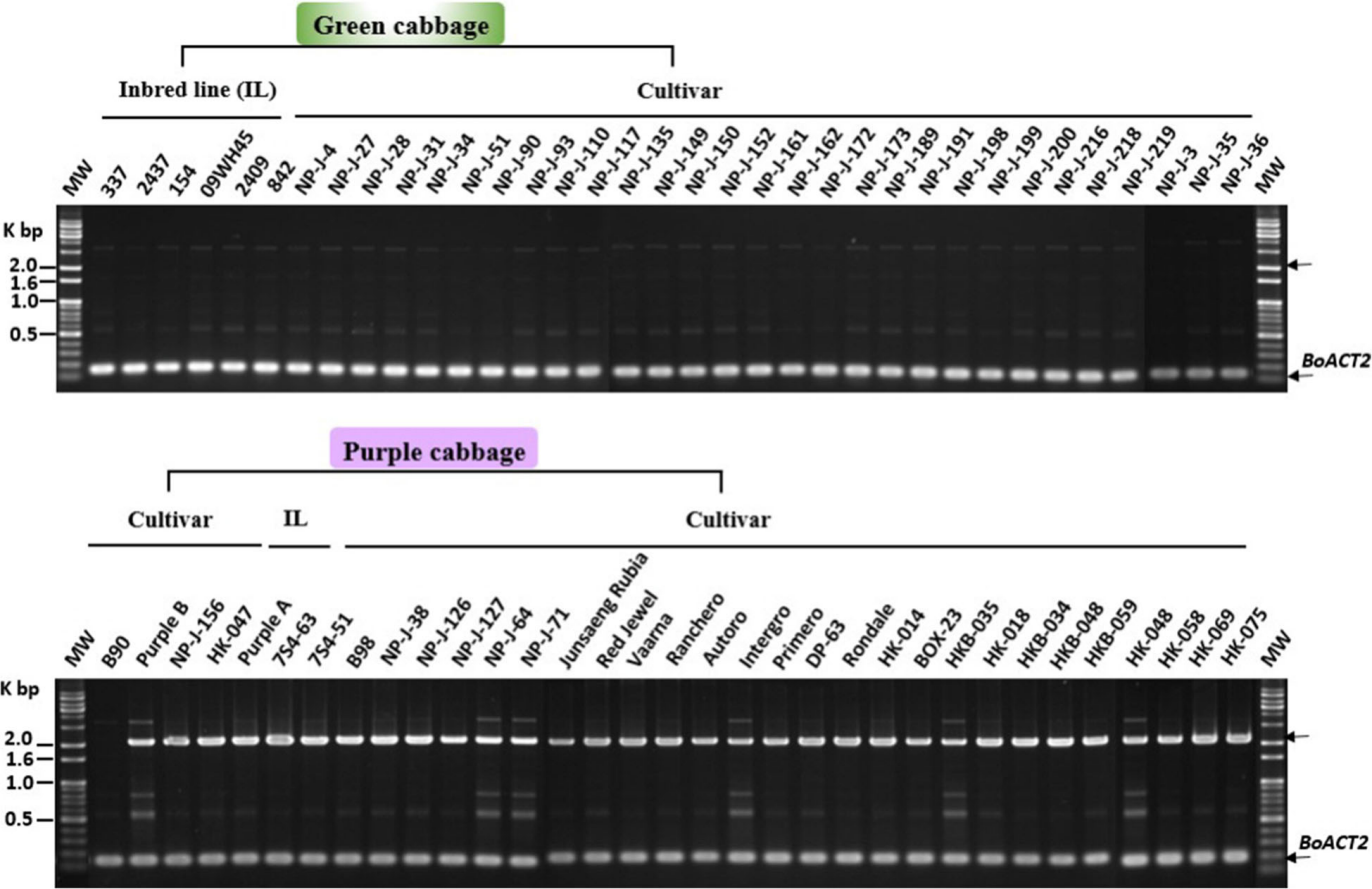
PCR analysis using the F3 and R3 primer pair to amplify the substituted region of *BoMYBL2–1* (upper arrow). All purple cabbages other than B90 (left hand side) contain the substituted DNA region

**Fig. 8:**
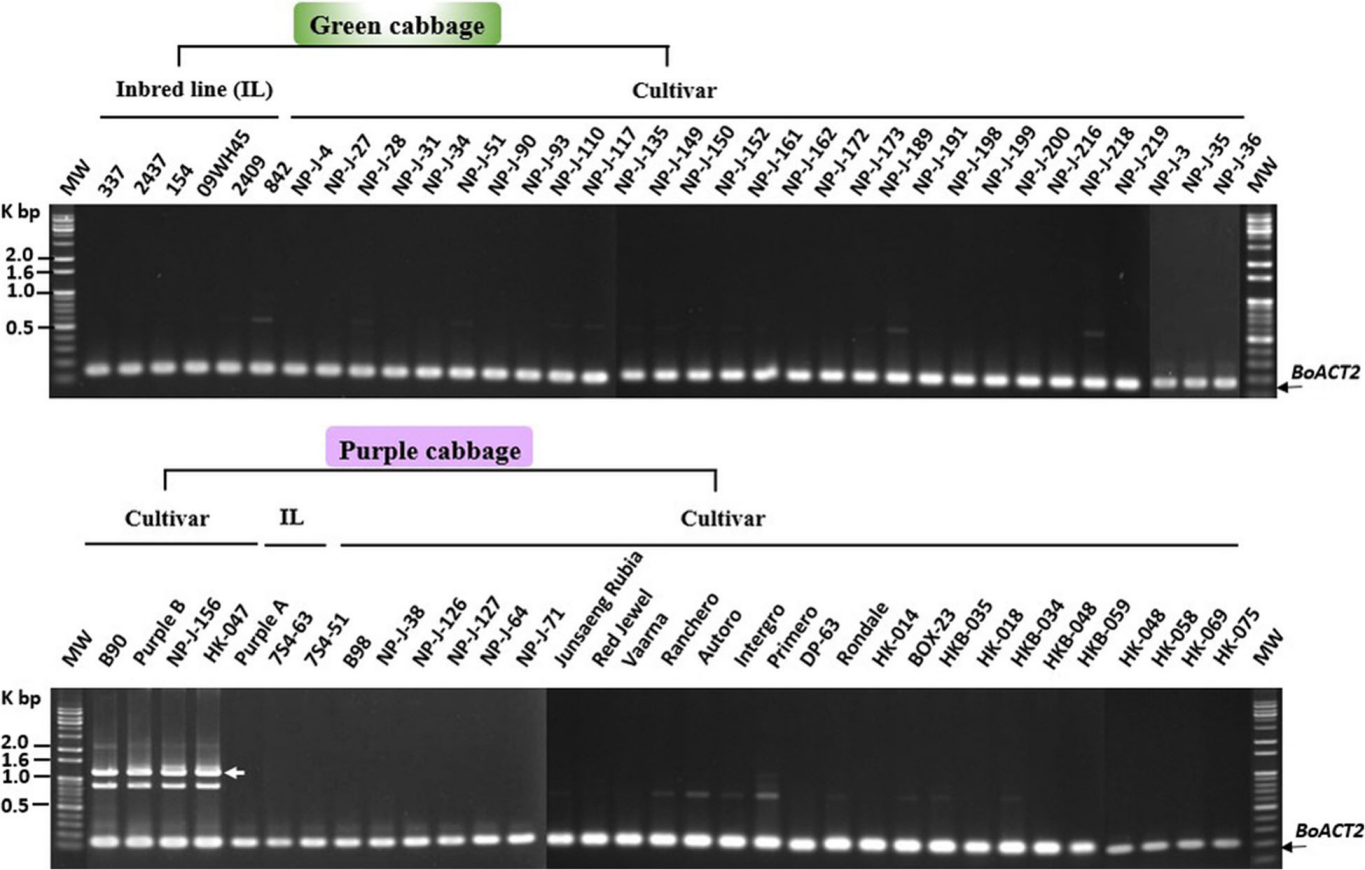
Results of PCR analysis using the F2 and R2 primer pair (BoMYBL2v-F and BoMYBL2v-R) to amplify *BoMYBL2–1*v, which contains the substituted region of the promoter. The white arrow indicates the position of the predicted PCR product. Three purple cabbages (Purple B, NP-J-156, and HK-047) were heterozygous (*BoMYBL2–1v/BoMYBL2–1sub*), but B90 was homozygous for *BoMYBL2–1v*. All other purple cabbages were homozygous for *BoMYBL2–1sub*

As shown in Fig. 6, the band corresponding to *BoMYBL2–1* coding sequence was amplified by the F1 and R1 primers (BoMYBL2w-F and BoMYBL2w-R) from all the green cabbages tested, as well as from four purple cabbages (B90, Purple B, NP-J-156, and-047). All of the purple cabbages tested, however, other than B90, contained DNA that could be amplified using the F3 and R3 primers (BoMYBL2sub-F and BoMYBL2sub-R) (Fig. 7), implying that these plants contained the substituted version of the entire *BoMYBL2–1* gene, including the promoter and coding regions. In addition, only four purple cabbages contained DNA that could be amplified using the F2 and R2 primers (BoMYBL2v-F and BoMYBL2v-R), which were specific for the promoter-substituted *BoMYBL2–1* variant (*BoMYBL2–1v*) (Fig. 8). Taken together, we concluded that purple cabbages contain either *BoMYBL2–1v* or *BoMYBL2–1sub* or both. Our data indicated that most of the purple cabbages in this study were homozygous for *BoMYBL2–1sub*, although B90 was homozygous for *BoMYBL2–1v*. Purple B, NP-J-0156, and HK-047 were heterozygous, carrying both the *BoMYBL2–1v* and *BoMYBL2–1sub* alleles.

## Discussion

Purple or red color in *Brassica* species due to anthocyanin accumulation is usually related to the activation of anthocyanin biosynthesis genes and/or their transcriptional activators. Purple or red color in *B. oleracea* is associated with an increase in expression of transcriptional activators, such as *BoMYB2* and *BoTT8* (*BobHLH*) [44–46], and *BoPAP1* and *BoPAP2* [47, 48]. To date, only two studies have reported natural mutations that lead to an increase in anthocyanin biosynthesis: mutation of the *BoMYB2* promoter to activate its expression in purple cauliflower [44], and deletion of the *DFR* gene in purple ornamental kale [55]. There is, however, no information available on the origin of red or purple cabbage (*Brassica oleracea* L. var. *capitate* f, *rubra*), a native of the Mediterranean region of Europe that is now grown as a fresh market vegetable all over the world [46]. The present study found that purple color in heading-type cabbages resulted from defective expression of *BoMYBL2–1* caused by two different mechanisms: either a substitution in the *BoMYBL2–1* promoter or a deletion of the entire gene. This is the first report showing that purple plants can be spontaneously generated by a defective repressor, rather than by alteration of activator expression by transposon insertion.

MYBL2, a small MYB protein that has two functional transcriptional repressor motifs, EAR and TLLLER motifs, interacts with TT8, a bHLH protein that is a component of the MBW complex, and represses expression of anthocyanin biosynthesis genes (LBGs) [24, 29, 33, 35–37]. MBW complexes control the flavonoid biosynthesis pathway at the transcriptional level developmentally and environmentally, mainly by activating expression of flavonoid LBGs [29]. MYBL2 and other R2R3-MYBs interact with bHLH proteins in a competitive manner; thus other R2R3-MYBs prevent the formation of the MBW complex and so negatively regulate anthocyanin production [24, 28, 35]. Repression or sequestering of MYBL2 activates anthocyanin biosynthesis in *Arabidopsis* [41–43].

The *B. oleracea* var. *capitata* genome contains two *MYBL2* genes, *BoMYBL2–1* and *BoMYBL2–2*; however, only *BoMYBL2–1* appears to be closely associated with anthocyanin production in cabbages, as a decrease in *BoMYBL2–1* expression was observed in some cultivars of purple cabbages (Fig. 1). These expression data, together with studies of *BoMYBL2–1* promoter activity in transgenic *Arabidopsis* (Figs. 5 and 6), supported the conclusion that the absence of *BoMYBL2–1* expression, as a result of either promoter substitution or whole gene deletion, was responsible for the production of purple color in cabbage. However, we cannot rule out the possibility that other negative regulators are also involved in the generation of other purple cabbages not included in the current study, as has been observed in poplar [60, 61].

**Fig. 9:**
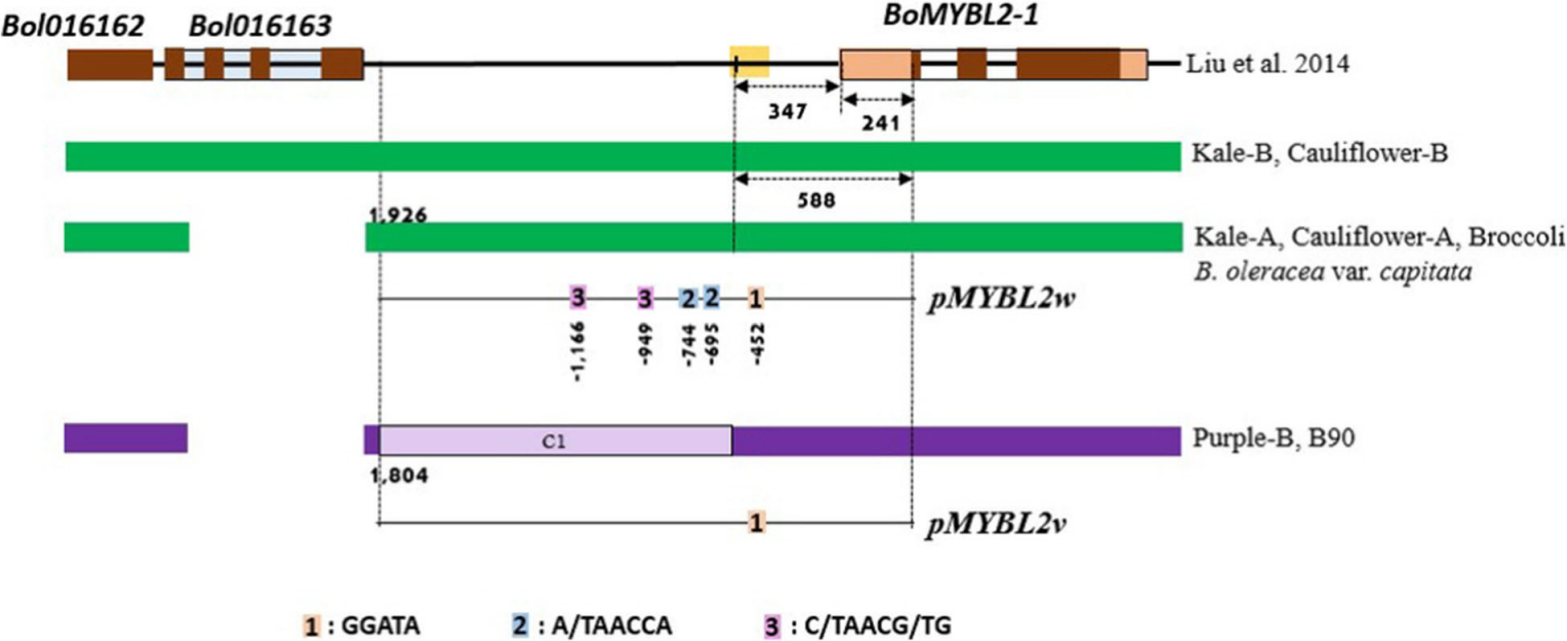
Schematic representation comparing the promoter sequences and *cis*-elements in *BoMYBL2w* and *BoMYBL2v*. The *BoMYBL2w* promoter harbors one MYB core motif GGATA (1; Baranowskij et al. 1994), two MYB recognition sequences (2; A/TAACCA), and two MYB recognition sequences (3; C/TAACG/TG; Abe et al. 2003). The upstream sequence of *BoMYBL2v* contains only one MYB core motif

The loss of promoter function in the substituted promoter of *BoMYBL2–1* present in several purple cabbage cultivars was supported by an analysis of GUS expression in transgenic *Arabidopsis* (Figs. 5 and 6). As shown in Fig. 9, the *BoMYBL2–1w* promoter harbors three types of MYB binding motifs: one MYB core motif (GGATA) [59], two MYB recognition sequences (A/TAACCA), and two MYB recognition sequences (C/TAACG/TG) [62]. By contrast, the upstream sequences of the *BoMYBL2–1v* coding sequence found in two purple cabbages contain only one MYB core motif, which is located in a 159 bp repeat sequence. This implies that one MYB core motif alone is not sufficient for the initiation of *BoMYBL2–1v* expression, at least in *B. oleracea* and *Arabidopsis*. It is not yet clear when this allele was first introduced to *B. oleracea* and whether the loss of *BoMYBL2–1* has any adaptive value.

We detected high levels of sequence variation around *BoMYBL2–1* and the surrounding region (Fig. 3; Additional file 7: Figure S5). This may be due to genome assembly errors in the ‘02–12’ reference genome of cabbage (http://brassicadb.org) [57] and the TO1000 sequence (http://plants.ensembl.org/Brassica_oleracea) [57]. Similar assembly errors in cabbage reference genomes have been identified and corrected for mapping of clubroot resistance [63] and yellow-green leaf trait [64]. We provide the sequences flanking *BoMYBL2–1* in chromosome 6 obtained in this study, which suggest that *Bol016163* is absent from *B. oleracea* var. *capitata* (Additional file 5; Additional file 7: Figure S5).

As shown in Additional file 6: Figure S3 and Additional file 8: Figure S4, it is clear that all purple cabbages (*B. oleracea* var. *capitata* f, *rubra*) retain most of the genes associated with anthocyanin biosynthesis and regulation. Most studies of the development of purple or red coloration in crops suggest that it results from activation of anthocyanin biosynthesis genes and/or regulatory genes, and is largely due to mutation, especially mutations within a promoter that confer constitutive expression [44, 49–55]. The present study demonstrates, however, that mutation within repressor genes provides another route to the establishment of anthocyanin-enriched plants in nature, and suggests that similar types of mutation may be present in crops with high anthocyanin content.

We developed a method for using PCR-based molecular markers that could quickly identify and distinguish purple cabbages that contain a substituted promoter or a complete deletion of *BoMYBL2–1* (Figs. 6, 7 and 8). These markers will facilitate the intraspecific and interspecific breeding of *B. oleracea* to produce anthocyanin-rich crops. Unfortunately, these markers do not predict the anthocyanin levels or profiles of different purple cabbages, even though a previous study reported genotype-dependent variations in anthocyanin profiles [22]. A more detailed understanding of anthocyanin biosynthesis in cabbages is required to develop additional markers to select purple-colored cabbages possessing more desirable anthocyanin profiles.

## Conclusion

*B. oleracea* var. *capitata* plants possess two *MYBL2* genes: *BoMYBL2–1* and *BoMYBL2–2*. Expression levels of *BoMYBL2–1* are inversely correlated to anthocyanin content, particularly in purple cabbage cultivars with defective expression of *BoMYBL2–1*. We found that most purple cabbages do not have an entire *BoMYBL2–1* gene, due to deletion of the coding sequence and the substitution of the regulatory region with other DNA sequences, while others contain a version of the *BoMYBL2–1* promoter that lacks transcription activity. We used sequence variations of *BoMYBL2–1* to develop and validate molecular markers that distinguish purple cabbage cultivars with defective *BoMYBL2–1*. These markers will be useful in the production of intraspecific and interspecific hybrids that contain high anthocyanin levels useful for functional foods and industrial use.

## Additional files

Additional file 1: **Table S1.** Primers used to clone *BoMYBL2.* (DOCX 19 kb)

Additional file 2: **Figure S2.** Schematic illustration of the strategy used to clone *BoMYBL2–1* from various *B. oleracea* species. A: Genomic region around the *BoMYBL2–1* based on information obtained from two different databases (upper: http://brassicadb.org/brad; Liu et al. 2014; lower: http://plants.ensembl.org/Brassica_oleracea/Info/Index; Parkin et al. 2014). B: Illustration showing the positions of the fragments, amplified using different combinations of primer sets, used to assemble the entire promoter and coding sequence of *BoMYBL2–1*. The yellow block represents a 159 bp repeat sequence. All primer sets are listed in Table 2. *BoLPR2* is *B. oleracea* multicopper oxidase LPR2; *BoPUB10* is *B. oleracea* U-box domain-containing protein 10. (DOCX 72 kb)

Additional file 3: **Table S2.** List of primer sequences used in RT-PCR and genomic PCR analyses of *BoMYBL2.* (DOCX 32 kb)

Additional file 4: **Figure S1.** Total anthocyanin content (A) of samples of the cabbages (B) shown in Fig. 1. (DOCX 109 kb)

Additional file 5: DNA sequences of BoMYBL2–1 plus the front region or corresponding region of the gene. Gene names represented plant names descirbed in Table 1. TO1000 indicates reference sequence (Parkins et al. 2014). (TXT 86 kb)

Additional file 6: **Figure S3.** Results of genomic DNA-PCR preformed to detect the presence or absence of genes regulating anthocyanin biosynthesis other than *BoMYBL2–1*. A: *B. oleracea* var. *capitata F. alba* or *rubra* varieties with contrasting characteristics in anthocyanin accumulation were selected for analysis; varieties with green and purple colors are indicated in green or purple. B: PCR analysis of other varieties of *B. oleracea*. The color of each variety is indicated above each lane. (DOCX 293 kb)

Additional file 7: **Figure S5.** Comparison of different *BoMYBL2–1* nucleotide sequences obtained from cabbages. Shaded regions indicate exon sequences. Sequences corresponding to *Bol016162* from *B. oleracea* var. *capitata* were omitted. (DOCX 31 kb)

Additional file 8: **Figure S4.** Expression of genes associated with anthocyanin biosynthesis in various purple cabbages. Daebakna is a green cabbage used as a reference. (DOCX 387 kb)

## Abbreviations

ANR: Athocyanidin reductase
bHLH: Basic helix-loop-helix
bHLH042: TT8
BIN2: BRASSOSTERROIS INSENSITIVE 2
BoMYBH: MYBD homolog
CHI: Chalcone isomerase
CHS: Chalcone synthase
CHS: Chalcone synthase (TT4)
DFR: Dihydorflavonol 4-reductase (TT3)
DFR: Dihydroflavonol 4-reductase
EBGs: Early biosynthesis genes
EGL3: Enhancer of glabra 3
EGL3: ENHANCER OF GLABRA3/AtMYC-2 MYB123 (TT2)
F3’H: Flavanone 3′-hydroxylase (TT7)
F3’H: Flavonoid 3′-hydroxylase
F3H: Flavanone 3-hydorxylase/TT6
F3H: Flavanone 3-hydroxylase
FLS: Flavono synthase
GL1: GLABROUS1
GL3: Glabra3
GL3: GLABROUS3
GST: Glutathione S-transferase
GUS: ß-glucuronidase
iPCR: Inverse PCR
LBGs: Late biosynthetic genes
LDOX: Leucoanthocyanidin dioxygenase
MBW: MYB-bHLH-WD40 complex
MYB12: PRODUCTION OF FLAVONOL GLYCOSIDES 1
MYBL2: MYB-like 2
MYN11: SQUAMOSA PROMOTER BINDING PROTEIN-LIKE9
PAP1: Production of anthocyanin pigment 1
PAP2: Production of anthocyanin pigment 1
qRT-PCR: quantitative reverse transcription polymerase chain reaction
SPL: SQUAMOSA PROMOTER BINDING PROTEIN-LIKE9
TT2: TRANSPARENT TESTA 2
TT8: Transparent testa 8
TTG1: Transparent testa glabra 1
UD3GT: UDP-glucose: flavonoid 3-*O*-glucosyltransferase,
UFGT: UDP-flavonoid glucosyl transferase

## References

[1] Chandler S, Tanaka Y (2007). Genetic modification in floriculture. Crit Rev Plant Sci.

[2] Chandler SF, Brugliera F (2011). Genetic modification in floriculture. Biotechnol Lett.

[3] Wessinger CA, Rausher MD (2012). Lessons from flower colour evolution on targets of selection. J Exp Bot.

[4] Bradshaw HD, Schemske DW (2013). Allele substitution at a flower colour locus produces a pollinator shift in monkeyflowers. Nature.

[5] Lorenc-Kukuła K, Jafra S, Oszmiański J, Szopa J (2005). Ectopic expression of anthocyanin 5-o-glucosyltransferase in potato tuber causes increased resistance to bacteria. J Agric Food Chem.

[6] Castellarin SD, Pfeiffer A, Sivilotti P, Degan M, Peterlunger E, DI Gaspero G (2007). Transcriptional regulation of anthocyanin biosynthesis in ripening fruits of grapevine under seasonal water deficit. Plant Cell Environ.

[7] Buer CS, Imin N, Djordjevic MA (2010). Flavonoids: new roles for old molecules. J Integr Plant Biol.

[8] Winkel-Shirley B (2002). Biosynthesis of flavonoids and effects of stress. Curr Opin Plant Biol.

[9] He J, Giusti MM (2010). Anthocyanins: natural colorants with health-promoting properties. Annu Rev Food Sci Technol.

[10] Bártiková H, Skálová L, Dršata J, Boušová I (2013). Interaction of anthocyanin with drug-metabolizing and antioxidant enzymes. Curr Med Chem.

[11] Nakabayashi R, Yonekura-Sakakibara K, Urano K, Suzuki M, Yamada Y, Nishizawa T, Matsuda F, Kojima M, Sakakibara H, Shinozaki K, Michael AJ, Tohge T, Yamazaki M, Saito K (2014). Enhancement of oxidative and drought tolerance in *Arabidopsis* by overaccumulation of antioxidant flavonoids. Plant J.

[12] Wang Y, Zhao L, Wang D, Huo Y, Ji B (2015). Anthocyanin-rich extracts from blackberry, wild blueberry, strawberry, and chokeberry: antioxidant activity and inhibitory effect on oleic acid-induced hepatic steatosis in vitro. J Sci Food Agric.

[13] Ghosh D, Konishi T (2007). Anthocyanins and anthocyanin-rich extracts: role in diabetes and eye function. Asia Pac J Clin Nutr.

[14] Shim SH, Kim JM, Choi CY, Kim CY, Park KH (2012). *Ginkgo biloba* extract and bilberry anthocyanins improve visual function in patients with normal tension glaucoma. J Med Food.

[15] Hannum SM (2004). Potential impact of strawberries on human health: a review of the science. Crit Rev Food Sci Nutr.

[16] de Pascual-Teresa S, Moreno DA, Garcia-Viguera C (2010). Flavanols and anthocyanins in cardiovascular health: a review of current evidence. Int J Mol Sci.

[17] Huang PC, Kuo WW, Shen CY, Chen YF, Lin YM, Ho TJ, Padma VV, Lo JF, Huang CY, Huang CY. Anthocyanin attenuates doxorubicin-induced cardiomyotoxicity via estrogen receptor-α/β and stabilizes HSF1 to inhibit the IGF-IIR apoptotic pathway. Int J Mol Sci. 2016;17(9):1588. 10.3390/ijms17091588 PMC5037853 27657062

[18] Li D, Zhang Y, Liu Y, Sun R, Xia M (2015). Purified anthocyanin supplementation reduces dyslipidemia, enhances antioxidant capacity, and prevents insulin resistance in diabetic patients. J Nutr.

[19] Cerletti C, de Curtis A, Bracone F, Digesù C, Morganti AG, Iacoviello L, de Gaetano G, Donati MB (2017). Dietary anthocyanins and health: data from FLORA and ATHENA EU projects. Br J Clin Pharmacol.

[20] Liu W, Xu J, Liu Y, Yu X, Tang X, Wang Z, Li X (2014). Anthocyanins potentiate the activity of trastuzumab in human epidermal growth factor receptor 2-positive breast cancer cells in vitro and in vivo. Mol Med Rep.

[21] Lin BW, Gong CC, Song HF, Cui YY (2017). Effect of anthocyanins on the prevention and treatment of cancer. Br J Pharmacol.

[22] Wiczkowski W, Topolska J, Honke J (2014). Anthocyanins profile and antioxidant capacity of red cabbages are influenced by geneotype and vegetation period. J Funct Foods.

[23] Lepiniec L, Debeaujon I, Routaboul JM, Baudry A, Pourcel L, Nesi N, Caboche M (2006). Genetics and biochemistry of seed flavonoids. Annu Rev Plant Biol.

[24] Dubos C, Gourrierec JL, Baudry A, Huep G, Lanet E, Debeaujon I, Routaboul JM, Alboresi A, Weisshaar B, Lepiniec L (2008). MYBL2 is a new regulator of flavonoid biosynthesis in *Arabidopsis thaliana*. Plant J.

[25] Zimmermann IM, Heim MA, Weisshaar B, Uhrig JF (2004). Comprehensive identification of *Arabidopsis thaliana* MYB transcription factors interacting with R/B-like BHLH proteins. Plant J.

[26] Mehrtens F, Kranz H, Bednarek P, Weisshaar B (2005). The *Arabidopsis* transcription factor MYB12 is a flavonol-specific regulator of phenylpropanoid biosynthesis. Plant Physiol.

[27] Stracke R, Ishihara H, Huep G, Barsch A, Mehrtens F, Niehaus K, Weisshaar B (2007). Differential regulation of closely related R2R3-MYB transcription factors controls flavonol accumulation in different parts of the *Arabidopsis thaliana* seedling. Plant J.

[28] Li S (2014). Transcriptional control of flavonoid biosynthesis: fine-tuning of the MYB-bHLH-WD40 (MBW) complex. Plant Signal Behav.

[29] Xu W, Dubos C, Lepiniec L (2015). Transcriptional control of flavonoid biosynthesis by MYB-bHLH-WDR complexes. Trend Plant Sci.

[30] Debeaujon I, Nesi N, Perez P, Devic M, Grandjean O, Caboche M, Lepiniec L (2003). Proanthocyanidin-accumulating cells in *Arabidopsis* testa: regulation of differentiation and role in seed development. Plant Cell.

[31] Baudry A, Heim MA, Dubreucq B, Caboche M, Weisshaar B, Lepiniec L (2004). TT2, TT8, and TTG1 synergistically specify the expression of *BANYULS* and proanthocyanindin biosynthesis in *Arabidopsis thaliana*. Plant J.

[32] Xie Y, Tan H, Ma Z, Huang J (2016). DELLA proteins promote anthocyanin biosynthesis via sequestering MYBL2 and JAZ suppressors of the MYB/bHLH/WD40 complex in *Arabidopsis thaliana*. Mol Plant.

[33] Yanhui C, Xiaoyuan Y, Kun H, Meihua L, Jigang L, Zhaofeng G, Zhiqiang L, Yunfei Z, Xiaoxiao W, Xiaoming Q, Yunping S, Li Z, Xiaohui D, Jingchu L, Xing-Wang D, Zhangliang C, Hongya G, Li-Jia Q (2006). The MYB transcription factor superfamily of *Arabidopsis*: expression analysis and phylogenetic comparison with the rice MYB family. Plant Mol Biol.

[34] Ohta M, Matsui K, Hiratsu K, Shinshi H, Ohme-Takagi M (2001). Repression domains of class II ERF transcriptional repressors share an essential motif for active repression. Plant Cell.

[35] Matsui K, Umemura Y, Ohme-Takagi M (2008). AtMYBL2, a protein with a single MYB domain, acts as a negative regulator of anthocyanin biosynthesis in *Arabidopsis*. Plant J.

[36] Kagale S, Rozwadowski K (2010). Small yet effective: the ethylene responsive element binding factor-associated amphiphilic repression (EAR) motif. Plant Signal Behav.

[37] Kagale S, Rozwadowski K (2011). EAR motif-mediated transcriptional repression in plants: an underlying mechanism for epigenetic regulation of gene expression. Epigenetics.

[38] Albert NW, Davies KM, Lewis DH, Zhang H, Montefiori M, Brendolise C, Boase MR, Ngo H, Jameson PE, Schwinn KE (2014). A conserved network of transcriptional activators and repressors regulates anthocyanin pigmentation in eudicots. Plant Cell.

[39] Ye H, Li L, Guo H, Yin Y (2012). MYBL2 is a substrate of GSK3-like kinase BIN2 and acts as a corepressor of BES1 in brassinosteroid signaling pathway in *Arabidopsis*. Proc Natl Acad Sci U S A.

[40] Zhang Y, Chen G, Dong T, Pan Y, Zhao Z, Tian S, Hu Z (2014). Anthocyanin accumulation and transcriptional regulation of anthocyanin biosynthesis in purple box choy (*Brassica rapa* var. *chinensis*). J Agric Food Chem.

[41] Nguyen NH, Jeong CY, Kang GH, Yoo SD, Hong SW, Lee H (2015). MYBD employed by HY5 increases anthocyanin accumulation *via* repression of *MYBL2* in *Arabidopsis*. Plant J.

[42] Wang Y, Wang Y, Song Z, Zhang H (2016). Repression of *MYBL2* by both microRNA858a and HY5 leads to the activation of anthocyanin biosynthetic pathway in *Arabidopsis*. Mol Plant.

[43] Xie Q, Hu Z, Zhang Y, Taian S, Wang Z, Zhao Z, Yang Y, Chen G (2014). Accumulation and molecular regulation of anthocyanin in purple tumorous stem mustard (*Brassica juncea* var. *tumida* Tsen et lee). J Agric Food Chem.

[44] Chiu LW, Zhou X, Burke S, Wu X, Prior RL, Li L (2010). The purple cauliflower arises from activation of a MYB transcription factor. Plant Physiol.

[45] Chiu LW, Li L (2012). Characterization of the regulatory network of BoMYB2 in controlling anthocyanin biosynthesis in purple cauliflower. Planta.

[46] Yuan Y, Chiu LW, Li L (2009). Transcriptional regulation of anthocyanin biosynthesis in red cabbage. Planta.

[47] Zhang B, Hu Z, Zhang Y, Li Y, Zhou S, Chen G (2012). A putative functional MYB transcription factor induced by low temperature regulates anthocyanin biosynthesis in purple kale (*Brassica oleracea* var. *acephala f. tricolor*). Plant Cell Rep.

[48] Zhang Y, Hu Z, Zhu M, Zhu Z, Wang Z, Tian S, Chen G (2015). Anthocyanin accumulation and molecular analysis of correlated genes in purple kohlrabi (*Brassica oleracea* var. *gongylodes* L.). J Agr Food Chem.

[49] Chagné D, Carlisle C, Blond C, Volz R, Whitworth C, Oraguzie N, Crowhurst R, Allan A, Espley R, Hellens R, Gardiner S (2007). Mapping a candidate gene (*MdMYB10*) for red flesh and foliage colour in apple. BMC Genomics.

[50] Espley RV, Brendolise C, Chagne D, Kutty-Amma S, Green S, Volz R, Putterill J, Schouten HJ, Gardiner SE, Hellens RP, Allan AC (2009). Multiple repeats of a promoter segment causes transcription factor autoregulation in red apples. Plant Cell.

[51] Kim S, Binzel ML, Yoo KS, Park S, Pike LM (2004). *Pink* (*P*), a new locus responsible for a pink trait in onions (*Allium cepa*) resulting from natural mutations of anthocyanidin synthase. Mol Gen Genomics.

[52] Kim S, Park JY, Yang TJ (2015). Characterization of three active transposable elements recently inserted in three independent *DFR-A* alleles and one high-copy DNA transposon isolated from the *Pink* allele of the ANS gene in onion (*Allium cepa* L.). Mol Gen Genomics.

[53] Tuteja JH, Clough SJ, Chan WC, Vodkin LO (2004). Tissue-specific gene silencing mediated by a naturally occurring *Chalcone synthase* gene cluster in *Glycine max*. Plant Cell.

[54] Tuteja JH, Zabala G, Varala K, Hudson M, Vodkin LO (2009). Endogenous, tissue-specific short interfering RNAs silence the *Chalcone synthase* gene family in *Glycine max* seed coats. Plant Cell.

[55] Liu XP, Gao BZ, Han FQ, Fang ZY, Yang LM, Zhuang M, Lv HH, Liu YM, Li ZS, Cai CC, Yu HL, Li ZY, Zhang YY (2017). Genetics and fine mapping of a purple leaf gene, *BoPr*, in ornamental kale (*Brassica oleracea* L. var. *acephala*). BMC Genomics.

[56] Parkin IA, Koh C, Tang H, Robinson SJ, Kagale S, Clarke WE, Town CD, Nixon J, Krishnakumar V, Bidwell SL, Denoeud F, Belcram H, Links MG, Just J, Clarke C, Bender T, Huebert T, Mason AS, Pires JC, Barker G, Moore J, Walley PG, Manoli S, Batley J, Edwards D, Nelson MN, Wang X, Paterson AH, King G, Bancroft I, Chalhoub B, Sharpe AG (2014). Transcriptome and methylome profiling reveals relics of genome dominance in the mesopolyploid *Brassica oleracea*. Genome Biol.

[57] Liu S, Liu Y, Yang X, Tong C, Edwards D, Parkin IA, Zhao M, Ma J, Yu J, Huang S, Wang X, Wang J, Lu K, Fang Z, Bancroft I, Yang TJ, Hu Q, Wang X, Yue Z, Li H, Yang L, Wu J, Zhou Q, Wang W, King GJ, Pires JC, Lu C, Wu Z, Sampath P, Wang Z, Guo H, Pan S, Yang L, Min J, Zhang D, Jin D, Li W, Belcram H, Tu J, Guan M, Qi C, Du D, Li J, Jiang L, Batley J, Sharpe AG, Park BS, Ruperao P, Cheng F, Waminal NE, Huang Y, Dong C, Wang L, Li J, Hu Z, Zhuang M, Huang Y, Huang J, Shi J, Mei D, Liu J, Lee TH, Wang J, Jin H, Li Z, Li X, Zhang J, Xiao L, Zhou Y, Liu Z, Liu X, Qin R, Tang X, Liu W, Wang Y, Zhang Y, Lee J, Kim HH, Denoeud F, Xu X, Liang X, Hua W, Wang X, Wang J, Chalhoub B, Paterson AH (2014). The *Brassica oleracea* genome reveals the asymmetrical evolution of polyploid genomes. Nat Commun.

[58] Clough SJ, Bent AF (1998). Floral dip: a simplified method for *Agrobacterium*-mediated transformation of *Arabidopsis thaliana*. Plant J.

[59] Baranowskij N, Frohberg C, Prat S, Willmitzer L (1994). A novel DNA binding protein with homology to Myb oncoproteins containing only one repeat can function as a transcriptional activator. EMBO J.

[60] Yoshida K, Ma D, Constabel CP (2015). The MYB182 protein down-regulates proanthocyanidin and anthocyanin biosynthesis in poplar by repressing both structural and regulatory flavonoid genes. Plant Physiol.

[61] Wan S, Li C, Ma X, Luo K (2017). PtrMYB57 contributes to the negative regulation of anthocyanin and proanthocyanidin biosynthesis in poplar. Plant Cell Rep.

[62] Abe H, Urao T, Ito T, Seki M, Shinozaki K, Yamaguchi-Shinozaki K (2003). *Arabidopsis* AtMYC2 (bHLH) and AtMYB2 (MYB) function as transcriptional activators in abscisic acid signaling. Plant Cell.

[63] Lee J, Izzah NK, Choi BS, Joh HJ, Lee SC, Perumal S, Seo J, Ahn K, Jo EJ, Choi GJ, Nou IS, Yu Y, Tang TJ (2016). Genotyping-by-sequencing map permits identification of clubroot resistance QTLs and revision of the reference genome assembly in cabbage (*Brassica oleracea* L.). DNA Res.

[64] Liu XP, Yang C, Han FQ, Fang ZY, Yang LM, Zhuang M, Lv HH, Liu YM, Li ZS, Zhang YY (2016). Genetics and fine mapping of a yellow-green leaf gene (*ygl-1*) in cabbage (*Brassica oleracea* var. *capitata* L.). Mol Breed.

