# Supplementary material for: Purple Brassica oleracea var. capitata F. rubra is due to the loss of BoMYBL2–1 expression
Source: BMC Plant Biol. 2018 May 8;18:82. doi: 10.1186/s12870-018-1290-9 (PMC5941660; doi:10.1186/s12870-018-1290-9)
Supplement: Supplementary file 1 — Table S1. Primers used to clone BoMYBL2. (DOCX 19 kb) [file 12870_2018_1290_MOESM1_ESM.docx]

**Additional file 1: Table S1.** Primers used to clone *BoMYBL2*

| **# on Fig. 1B** | **Forward primer** | | **Reverse primer** | | | | **Product**  **(bp)** |
| --- | --- | --- | --- | --- | --- | --- | --- |
|  | **Name** | **Sequence** | | **Name** | | **Sequence** |  |
| **1** | F1 | 5'-CGTTATGTGAAGACCGCAAGGTAGA | | | R1 | 5'-CCCTTTGTCTAGCTCACCAGCA | 541 |
| **2** | F1 | .. | | | R2 | 5'-TGCTCTCCAAGTCCTTTCTTGCTC | 1268  (541) |
| **3** | F2 | 5'-ATGGCTGTTAAGAGCGGGAAAGTTGA | | | R3 | 5'-GAACAAGAAACGTTACCGGAAGGC | 2950  (2223) |
| **4** | F2 | .. | | | R4 | 5'-CTTGGCACGGTGCAGCATTCCT | 3121  (2394) |
| **5** | F3 | 5'-GCCTTCCGGTAACGTTTCTTGTTC | | | R5 | 5'-GTGAGTTAGTTCATTACTGGCGTGGA | 1178 |
| **6** | F1 |  | | | R5 |  | 4669  (4128) |
