# Supplementary material for: Purple Brassica oleracea var. capitata F. rubra is due to the loss of BoMYBL2–1 expression
Source: BMC Plant Biol. 2018 May 8;18:82. doi: 10.1186/s12870-018-1290-9 (PMC5941660; doi:10.1186/s12870-018-1290-9)
Supplement: Supplementary file 2 — Figure S2. Schematic illustration of the strategy used to clone BoMYBL2–1 from various B. oleracea species. A: Genomic region around the BoMYBL2–1 based on information obtained from two different databases (upper: http://brassicadb.org/brad; Liu et al. 2014; lower: http://plants.ensembl.org/Brassica_oleracea/Info/Index; Parkin et al. 2014). B: Illustration showing the positions of the fragments, amplified using different combinations of primer sets, used to assemble the entire promoter and coding sequence of BoMYBL2–1. The yellow block represents a 159 bp repeat sequence. All primer sets are listed in Table 2. BoLPR2 is B. oleracea multicopper oxidase LPR2; BoPUB10 is B. oleracea U-box domain-containing protein 10. (DOCX 72 kb) [file 12870_2018_1290_MOESM2_ESM.docx]

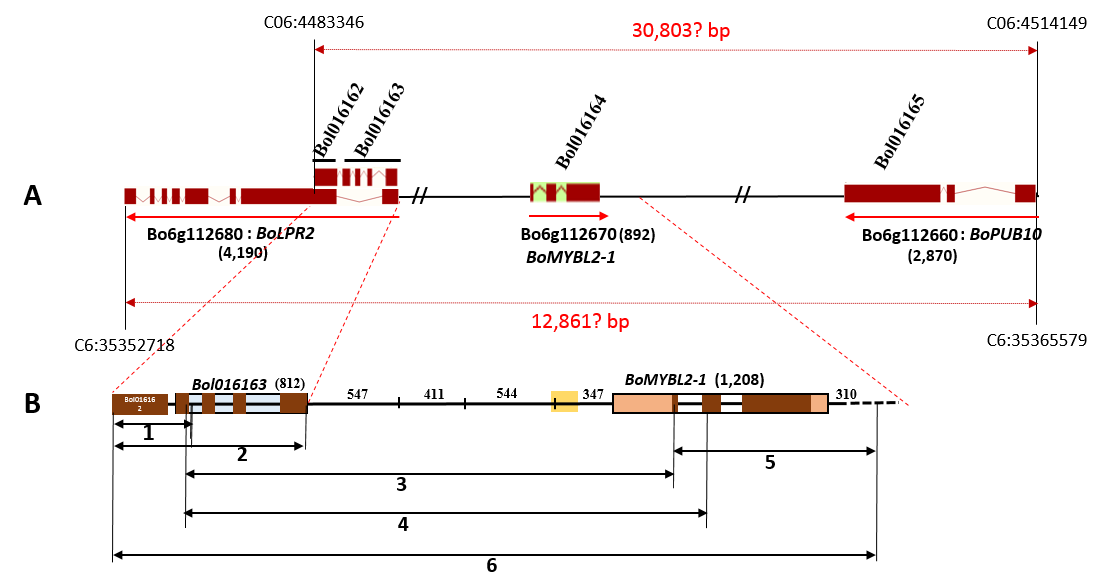


**Additional file 2: Figure S2.** Schematic illustration of the strategy used to clone *BoMYBL2-1* from various *B. oleracea* species. **A**: Genomic region around the *BoMYBL2-1* based on information obtained from two different databases (upper: <http://brassicadb.org/brad>; Liu et al. 2014; lower: <http://plants.ensembl.org/Brassica_oleracea/Info/Index>; Parkin et al. 2014). **B**: Illustration showing the positions of the fragments, amplified using different combinations of primer sets, used to assemble the entire promoter and coding sequence of *BoMYBL2-1*. The yellow block represents a 159 bp repeat sequence. All primer sets are listed in Table 2. *BoLPR2* is *B. oleracea* multicopper oxidase LPR2; *BoPUB10* is *B. oleracea* U-box domain-containing protein 10.
