# Supplementary material for: Purple Brassica oleracea var. capitata F. rubra is due to the loss of BoMYBL2–1 expression
Source: BMC Plant Biol. 2018 May 8;18:82. doi: 10.1186/s12870-018-1290-9 (PMC5941660; doi:10.1186/s12870-018-1290-9)
Supplement: Supplementary file 3 — Table S2. List of primer sequences used in RT-PCR and genomic PCR analyses of BoMYBL2. (DOCX 32 kb) [file 12870_2018_1290_MOESM3_ESM.docx]

**Additional file 3: Table S2.** List of primer sequences used in RT-PCR and genomic PCR analyses of *BoMYBL2.*

| Use | Forward primer | | Reverse primer | |
| --- | --- | --- | --- | --- |
|  | Name | Sequence | Name | Sequence |
| RT-PCR | BoMYB1-F | 5’-GGAAACAGGTGGTCTTTGCTGGTA | BoMYB1-R | 5’-ACTCCAAGGCATGGAGGAACAAC |
|  | BoMYB2-F | .. | BoMYB2-R | 5’-CGTTGAGTCCAAGGCATAGAGGAACAAT |
|  | BoMYBL2-1-1F (=1F) | 5‘-CTACCAGTCTCTCCTTTGAAGAAGAC | BoMYBL2-1-1R (=1R) | 5‘-GAGTTTTCCTTGATCTCACAGTACATTTCT |
|  | BoMYBL2-1-2F (=2F) | 5’-GAAGATTGCCTGGACGAACCGAC | BoMYBL2-1-2R (=2R) | 5‘-TGACTCGTTCGGCCATGGTTAGAGGA |
|  | BoMYBL2-2F | 5’-GTGCCGAGAGATGTAGAGTGGGA | BoMYBL2-2R | 5’-TCCGGCACTACTACTAGCACTACTA |
|  | BoDFR1-F | 5’-TATTACCGCGCTCTCTCCTATC | BpDFR1-R | 5’-ATCCCTGGTTCGGTCTTCTTA |
|  | BoACT2-F | 5’-TGCCAATCTACGAGGGTTTC | BoACT2-R | 5’-TCTCGATGGAAGAGCTGGTT |
| gDNA-PCR | BoMYBL2g-1F | 5’-CTACCAGTCTCTCCTTTGAAGAAGAC | BoMYBL2g-1R | 5’-GAGTTTTCCTTGATCTCACAGTACATTTCT |
| RT-PCR/  gDNA-PCR | BoMYB1-F | 5‘-CAATGGCTGCAGCCATCTCC | BoMYB1-R | 5‘-CCTTTTGGAACGAGCCCGTCA |
|  | BoMYB2-F | 5‘-TGAAGCCAAGTATCAAGAGAGGAAAACTC | BoMYB2-R | 5‘-TCATCTAGCAAACTTTCCCACCACC |
|  | BoMYB3-F | 5‘-GATCTTCTTCTTCGCCTTCATAAGCTTC | BoMYB3-R | 5‘-GATCTTCTGCTCCGCCTTCATAAACT |
|  | BoMYB4-F | 5‘-CCTTCCTTGTTCTTCTACTACACCAGC | BoMYB4-R | 5‘-ACTAACAAGCTCAGATTTATCGTCGTCT |
|  | BoMYBD-F2 | 5‘-CTCAGAAGTATTTCCTCCGCCGTAC | BoMYBD-R | 5‘-TCAAGCAACTCCCATTATACTATCTCCAT |
|  | BoSPL9-F | 5‘-TTGGTCCGATGACGGTTACAATGG | BoSPL9-R | 5‘-TCAGAGAGACCAGTTGTTATGGTGTGAA |
|  | BoMYBH-1-F2 | 5‘-CTGAACCAACCAAGAAAGAGGAGACTCA | BoMYBH-1-R | 5‘-TTAGACCGCATGTACCACGTTGGT |
|  | BoMYBH-2-F2 | 5‘-ACCCATTTCCATTCCCAGTATGGTCT | BoMYBH-2-R | 5‘-CTAAGCATTCGGGTGAAATGGTGATTGTC |
|  | BoMYB11-F | 5‘-AGACTCTGGACAAGCCAAAAGGTCT | BoMYB11-R | 5‘-CATGATCCAGTGGTTCTCCAGAGTTCT |
|  | BoMYB12-1F | 5‘-GTGTTTACATCGAGTGTTGGTGATGGG | BoMYB12-1R | 5‘-CCACGACACCACTGGATCAGAAG |
|  | BoMYB12-2F | 5‘-ATGCATGCAACGTGCTCACTTCA | BoMYB12-2R | 5‘-CCTCTTTCTCACCGCAAAGGCTATTC |
|  | BoCHS1-F | 5’-ACCCGGATACCTCTGCTGGA | BoCHS1-R | 5’-GGAGTTCCAGTCGCTTATCCCTAAG |
|  | BoCHS2-F | 5’-GGATCCTGATGCATCCGTAGGG | BoCHS2-R | 5’-GGAGTTCCAGTCACTTATCCCTAGC |
|  | BoF3H-F | 5‘-CAGATCGTTGAGGCTTGTGA | BoF3H-R | 5‘-TCAGTGTGACGCTTGAGTCC |
|  | BoF3’H-F | 5‘-CTCGCCGGAGTATTCAACAT | BoF3’-H-R | 5‘-ACCCTCACCGTCAAAATCAG |
|  | BoDFR-F | 5‘-TATTACCGCGCTCTCTCCTATC | BoDFR-r | 5‘-ATCCCTGGTTCGGTCTTCTTA |

**Table 5.** Primers related to genes involved in anthocyanin biosynthesis. These primer pairs were used for both genomic PCR and RT-PCR analyses

| **Gene** | **Forward primer** | | **Reverse primer** | |
| --- | --- | --- | --- | --- |
|  | **Name** | **Sequence** | **Name** | **Sequence** |
| ***BoPAP***  **(*Production of anthocyanin pigment*)** | BoMYB1-F | 5‘-CAATGGCTGCAGCCATCTCC | BoMYB1-R | 5‘-CCTTTTGGAACGAGCCCGTCA |
|  | BoMYB2-F | 5‘-TGAAGCCAAGTATCAAGAGAGGAAAACTC | BoMYB2-R | 5‘-TCATCTAGCAAACTTTCCCACCACC |
|  | BoMYB3-F | 5‘-GATCTTCTTCTTCGCCTTCATAAGCTTC | BoMYB3-R | 5‘-GATCTTCTGCTCCGCCTTCATAAACT |
|  | BoMYB4-F | 5‘-CCTTCCTTGTTCTTCTACTACACCAGC | BoMYB4-R | 5‘-ACTAACAAGCTCAGATTTATCGTCGTCT |
| ***BoMYBD*** | BoMYBD-F2 | 5‘-CTCAGAAGTATTTCCTCCGCCGTAC | BoMYBD-R | 5‘-TCAAGCAACTCCCATTATACTATCTCCAT |
| ***BoSPL9* (*Squamosa promoter-binding protein-like 9*)** | BoSPL9-F | 5‘-TTGGTCCGATGACGGTTACAATGG | BoSPL9-R | 5‘-TCAGAGAGACCAGTTGTTATGGTGTGAA |
| ***BoMYBH* (*MYBD homolog*)** | BoMYBH-1-F2 | 5‘-CTGAACCAACCAAGAAAGAGGAGACTCA | BoMYBH-1-R | 5‘-TTAGACCGCATGTACCACGTTGGT |
|  | BoMYBH-2-F2 | 5‘-ACCCATTTCCATTCCCAGTATGGTCT | BoMYBH-2-R | 5‘-CTAAGCATTCGGGTGAAATGGTGATTGTC |
| ***BoMYB11*** | BoMYB11-F | 5‘-AGACTCTGGACAAGCCAAAAGGTCT | BoMYB11-R | 5‘-CATGATCCAGTGGTTCTCCAGAGTTCT |
| ***BoMYB12*** | BoMYB12-1F | 5‘-GTGTTTACATCGAGTGTTGGTGATGGG | BoMYB12-1R | 5‘-CCACGACACCACTGGATCAGAAG |
|  | BoMYB12-2F | 5‘-ATGCATGCAACGTGCTCACTTCA | BoMYB12-2R | 5‘-CCTCTTTCTCACCGCAAAGGCTATTC |
| *Chalcone synthase 1* | BoCHS1-F | 5‘-ACCCGGATACCTCTGCTGGA | BoCHS1-R | 5‘-GGAGTTCCAGTCGCTTATCCCTAAG |
| *Chalcone synthase 2* | BoCHS2-F | 5‘-GGATCCTGATGCATCCGTAGGG | BoCHS2-R | 5‘-GGAGTTCCAGTCACTTATCCCTAGC |
|  | BoF3H-F | 5‘-CAGATCGTTGAGGCTTGTGA | BoF3H-R | 5‘-TCAGTGTGACGCTTGAGTCC |
|  | BoF3’H-F | 5‘-CTCGCCGGAGTATTCAACAT | BoF3’-H-R | 5‘-ACCCTCACCGTCAAAATCAG |
|  | BoDFR-F | 5‘-TATTACCGCGCTCTCTCCTATC | BoDFR-r | 5‘-ATCCCTGGTTCGGTCTTCTTA |
|  | BoACT2-F |  |  |  |

CHS: Chalcone synthase (TT4), F3H: Flavanone 3-hydroxylase/TT6, F3’H: Flavanone 3‘-hydroxylase (TT7), DFR: Dihydroflavonol 4-reductase (TT3).

SPL: SQUAMOSA PROMOTER-BINDING PROTEIN-LIKE 9, MYN11: SQUAMOSA PROMOTER-BINDING PROTEIN-LIKE 9, MYB12: PRODUCTION OF FLAVONOL GLYCOSIDES 1, ***BoMYBH* (*MYBD homolog*)**.

bHLH042 (TT8), GLABROUS3 (GL3), EGL3: ENHANCER OF GLABRA3/AtMYC-2, MYB123 (TT2), GL1: GLABROUS1.

BoPAP (BoMYB1-4): (Production of anthocyanin pigment), BoMYBH (MYBD homolog).
