# Supplementary material for: Purple Brassica oleracea var. capitata F. rubra is due to the loss of BoMYBL2–1 expression
Source: BMC Plant Biol. 2018 May 8;18:82. doi: 10.1186/s12870-018-1290-9 (PMC5941660; doi:10.1186/s12870-018-1290-9)
Supplement: Supplementary file 4 — Figure S1. Total anthocyanin content (A) of samples of the cabbages (B) shown in Fig. 1. (DOCX 109 kb) [file 12870_2018_1290_MOESM4_ESM.docx]

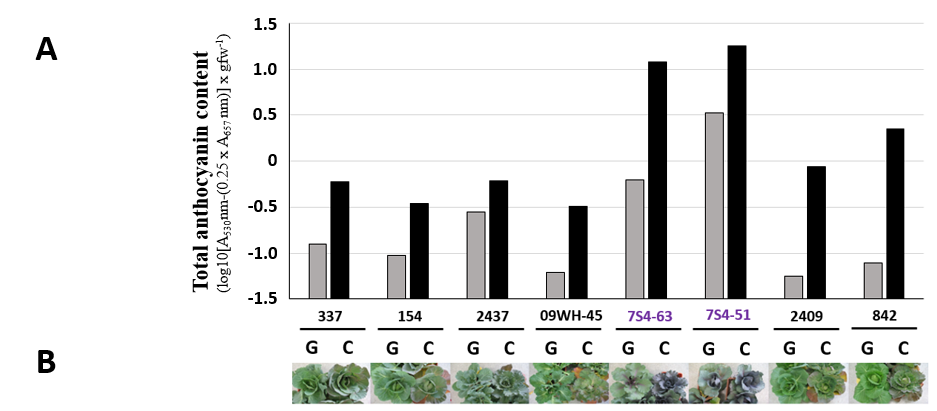


**Additional file 4: Figure S1.** Total anthocyanin content (A) of samples of the cabbages (B) shown in Figure 1.
