# Supplementary material for: Purple Brassica oleracea var. capitata F. rubra is due to the loss of BoMYBL2–1 expression
Source: BMC Plant Biol. 2018 May 8;18:82. doi: 10.1186/s12870-018-1290-9 (PMC5941660; doi:10.1186/s12870-018-1290-9)
Supplement: Supplementary file 6 — Figure S3. Results of genomic DNA-PCR preformed to detect the presence or absence of genes regulating anthocyanin biosynthesis other than BoMYBL2–1. A: B. oleracea var. capitata F. alba or rubra varieties with contrasting characteristics in anthocyanin accumulation were selected for analysis; varieties with green and purple colors are indicated in green or purple. B: PCR analysis of other varieties of B. oleracea. The color of each variety is indicated above each lane. (DOCX 293 kb) [file 12870_2018_1290_MOESM6_ESM.docx]

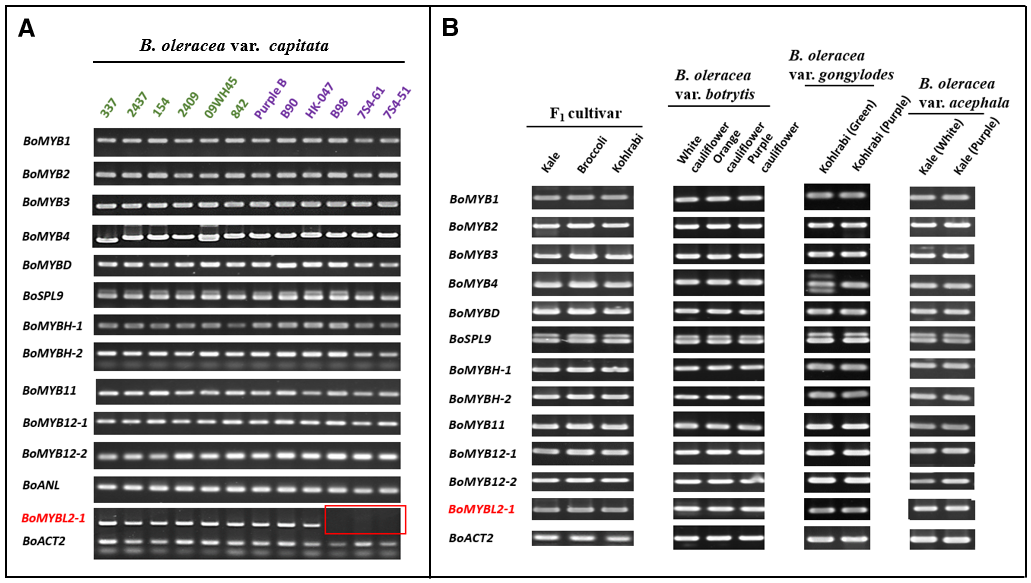


**Additional file 6: Figure S3.** Results of genomic DNA-PCR preformed to detect the presence or absence of genes regulating anthocyanin biosynthesis other than *BoMYBL2-1*. **A**: *B. oleracea* var. *capitata* f. *alba* or *rubra* varieties with contrasting characteristics in anthocyanin accumulation were selected for analysis; varieties with green and purple colors are indicated in green or purple. **B**: PCR analysis of other varieties of *B. oleracea*. The color of each variety is indicated above each lane.
