# Supplementary material for: Purple Brassica oleracea var. capitata F. rubra is due to the loss of BoMYBL2–1 expression
Source: BMC Plant Biol. 2018 May 8;18:82. doi: 10.1186/s12870-018-1290-9 (PMC5941660; doi:10.1186/s12870-018-1290-9)
Supplement: Supplementary file 7 — Figure S5. Comparison of different BoMYBL2–1 nucleotide sequences obtained from cabbages. Shaded regions indicate exon sequences. Sequences corresponding to Bol016162 from B. oleracea var. capitata were omitted. (DOCX 31 kb) [file 12870_2018_1290_MOESM7_ESM.docx]

| ***Bol016162***  TO1000 ATGTTGAAGAACAACAACTGCATACCTGATAACGCTCTTCTCAGCACTCTCATCCACTGGCTTTGCAAAGACGGGAGAGTTACAGAAGCGAGGAAGTTGTTGGACGAGTTTGAGAAAGGCTCCGTCCCTAGTCTCTTGACGTACAACACG  Kale_B ATGTTGAAGAACAA---CTGCATGCCTGATAACGCTCTTCTCAGCACTCTCATCCACTGGCTTTGCAGAGACGGGAGAGTTACAGAAGCGAGGAAGTTGTTGGACGAGTTTGAGAAAGGCTCGATCCCTAGTCTCTTGACGTACAACACG  Cauliflower_B ATGTTGAAGAACAA---CTGCATGCCTGATAACGCTCTTCTCAGCACTCTCATCCACTGGCTTTGCAGAGACGGGAGAGTTACAGAAGCGAGGAAGTTGTTGGACGAGTTTGAGAAAGGCTCGATCCCTAGTCTCTTGACGTACAACACG  154_1 ------------------------------------------------------------------------------------------------------------------------------------------------------  GreenB ------------------------------------------------------------------------------------------------------------------------------------------------------  154_2 ------------------------------------------------------------------------------------------------------------------------------------------------------  Broccoli ------------------------------------------------------------------------------------------------------------------------------------------------------  GreenA ------------------------------------------------------------------------------------------------------------------------------------------------------  2409_1 ------------------------------------------------------------------------------------------------------------------------------------------------------  2409_2 ------------------------------------------------------------------------------------------------------------------------------------------------------  09WH45 ------------------------------------------------------------------------------------------------------------------------------------------------------  842 ------------------------------------------------------------------------------------------------------------------------------------------------------  Kale_A ------------------------------------------------------------------------------------------------------------------------------------------------------  Cauliflower_A ------------------------------------------------------------------------------------------------------------------------------------------------------  B90_1 ------------------------------------------------------------------------------------------------------------------------------------------------------  B90_2 ------------------------------------------------------------------------------------------------------------------------------------------------------  PurpleB ------------------------------------------------------------------------------------------------------------------------------------------------------  TO1000 TTGATCTCTGGGATGTGCGAGAAGGGAGAGCTGATGGAGGCGGGCCGGTTATGGGACGACATGGTTGAGAGGAAGTGTAAGCCGAATGCTTTCACGTATAACGTTCTGATCGAAGGCCTGTGCAAGATGGGGAATGTTAAGGAAGGTGTC  Kale_B TTGATCTCTGGGATGTGCGAGAAGGGAGAGCTGACGGAGGCGGGTCGGTTATGGGACGACATGGTTGAGAGGAAGTGTAAGCCGAATGCTTTCACGTATAACGTTCTGATCGAAGGCCTGTGCAAGATGGGGAATGTTAAGGAAGGTGTC  Cauliflower_B TTGATCTCTGGGATGTGCGAGAAGGGAGAGCTGACGGAGGCGGGTCGGTTATGGGACGACATGGTTGAGAGGAAGTGTAAGCCGAATGCTTTCACGTATAACGTTCTGATCGAAGGCCTGTGCAAGATGGGGAATGTTAAGGAAGGTGTC  154_1 ------------------------------------------------------------------------------------------------------------------------------------------------------  GreenB ------------------------------------------------------------------------------------------------------------------------------------------------------  154_2 ------------------------------------------------------------------------------------------------------------------------------------------------------  Broccoli ------------------------------------------------------------------------------------------------------------------------------------------------------  GreenA ------------------------------------------------------------------------------------------------------------------------------------------------------  2409_1 ------------------------------------------------------------------------------------------------------------------------------------------------------  2409_2 ------------------------------------------------------------------------------------------------------------------------------------------------------  09WH45 ------------------------------------------------------------------------------------------------------------------------------------------------------  842 ------------------------------------------------------------------------------------------------------------------------------------------------------  Kale_A ------------------------------------------------------------------------------------------------------------------------------------------------------  Cauliflower_A ------------------------------------------------------------------------------------------------------------------------------------------------------  B90_1 ------------------------------------------------------------------------------------------------------------------------------------------------------  B90_2 ------------------------------------------------------------------------------------------------------------------------------------------------------  PurpleB ------------------------------------------------------------------------------------------------------------------------------------------------------  ***Bol016163***  TO1000 TTAAAGAGATGCTGGAGAATGGTTGTTTTCCGAATAAAACGAGGTTCTTGATATTGTTCGAGGGGTTGCAGGAGTCAGGGAAAGAAGAGGATGGGGTTAAGATTGTGTCGATGGCTGTTAAGAGCGGGAAAGTTGATAGAGAATGTTGGG  Kale_B TTAAAGAGATGCTGGAGAATGGTTGTTTTCCGAATAAAACGAGGTTCTTGATATTGTTCGAGGGGTTGCAGGAGTCAGGGAAAGAAGAGG--------------------ATGGCTGTTAAGAGCGGGAAAGTTGATAGAGAATGTTGGG  Cauliflower_B TTAAAGAGATGCTGGAGAATGGTTGTTTTCCGAATAAAACGAGGTTCTTGATATTGTTCGAGGGGTTGCAGGAGTCAGGGAAAGAAGAGG--------------------ATGGCTGTTAAGAGCGGGAAAGTTGATAGAGAATGTTGGG  154_1 ------------------------------------------------------------------------------------------------------------------------------------------------------  GreenB ------------------------------------------------------------------------------------------------------------------------------------------------------  154_2 ------------------------------------------------------------------------------------------------------------------------------------------------------  Broccoli ------------------------------------------------------------------------------------------------------------------------------------------------------  GreenA ------------------------------------------------------------------------------------------------------------------------------------------------------  2409_1 ------------------------------------------------------------------------------------------------------------------------------------------------------  2409_2 ------------------------------------------------------------------------------------------------------------------------------------------------------  09WH45 ------------------------------------------------------------------------------------------------------------------------------------------------------  842 ------------------------------------------------------------------------------------------------------------------------------------------------------  Kale_A ------------------------------------------------------------------------------------------------------------------------------------------------------  Cauliflower_A ------------------------------------------------------------------------------------------------------------------------------------------------------  B90_1 ------------------------------------------------------------------------------------------------------------------------------------------------------  B90_2 ------------------------------------------------------------------------------------------------------------------------------------------------------  PurpleB ------------------------------------------------------------------------------------------------------------------------------------------------------    TO1000 AGCTTTTCTTGAGGAAATTTGCTGGTGAGCTAGACAAAGGGGAGGTGGTTCTTAAAGAATTGTTGCATGAAGTTTCTGTCTCTTGAAGAAGCAAGGTGGTTCTTAAAAAAATAATGTTATAAGGGCAATGATCTAGATTCAAGGGATTTG  Kale_B AGCTTTTCTTGAGGAAATTTGCTGGTGAGCTAGACAAAGGGGAGGTGGTTCTTAAAGAATTGTTGCATGAAGTTTCTGTCTCTTGAAGAAGCAAGGTGGTTCTTAAAAAAATAATGTTATAAGGGCAATGATCTAGATTCAAGGGATTTG  Cauliflower_B AGCTTTTCTTGAGGAAATTTGCTGGTGAGCTAGACAAAGGGGAGGTGGTTCTTAAAGAATTGTTGCATGAAGTTTCTGTCTCTTGAAGAAGCAAGGTGGTTCTTAAAAAAATAATGTTATAAGGGCAATGATCTAGATTCAAGGGATTTG  154_1 ------------------------------------------------------------------------------------------------------------------------------------------------------  GreenB ------------------------------------------------------------------------------------------------------------------------------------------------------  154_2 ------------------------------------------------------------------------------------------------------------------------------------------------------  Broccoli ------------------------------------------------------------------------------------------------------------------------------------------------------  GreenA ------------------------------------------------------------------------------------------------------------------------------------------------------  2409_1 ------------------------------------------------------------------------------------------------------------------------------------------------------  2409_2 ------------------------------------------------------------------------------------------------------------------------------------------------------  09WH45 ------------------------------------------------------------------------------------------------------------------------------------------------------  842 ------------------------------------------------------------------------------------------------------------------------------------------------------  Kale_A ------------------------------------------------------------------------------------------------------------------------------------------------------  Cauliflower_A ------------------------------------------------------------------------------------------------------------------------------------------------------  B90_1 ------------------------------------------------------------------------------------------------------------------------------------------------------  B90_2 ------------------------------------------------------------------------------------------------------------------------------------------------------  PurpleB ------------------------------------------------------------------------------------------------------------------------------------------------------    TO1000 GTAGCACATAGCTTTATCAAAGGTTGGTTGGGTTTTGATCTGCTCCAAGGTAAGTACAACCCTGTGATCTCATAGCTCAGGGGAAAAAGGAAGAAGATGGCGTTTGTGACCACTGCGGAAGTCTGTGACGCAGCTGATTCGGAGTGGTGA  Kale_B GTAGCACATAGCTTTATCAAAGGTTGGTTGGGTTTTGATCTGCTCCAAGGTAAGTACAACCCTGTGATCTCATAGCTCAGGGGAAAAAGGAAGAAGATGGCGTTTGTGACCACTGCGGAAGTCTGTGACGCAGCTGATTCGGAGTGGTGA  Cauliflower_B GTAGCACATAGCTTTATCAAAGGTTGGTTGGGTTTTGATCTGCTCCAAGGTAAGTACAACCCTGTGATCTCATAGCTCAGGGGAAAAAGGAAGAAGATGGCGTTTGTGACCACTGCGGAAGTCTGTGACGCAGCTGATTCGGAGTGGTGA  154_1 ------------------------------------------------------------------------------------------------------------------------------------------------------  GreenB ------------------------------------------------------------------------------------------------------------------------------------------------------  154_2 ------------------------------------------------------------------------------------------------------------------------------------------------------  Broccoli -------------------------------------------------------------------------------------------------------------------------------------------------ATGTT  GreenA ------------------------------------------------------------------------------------------------------------------------------------------------------  2409_1 ------------------------------------------------------------------------------------------------------------------------------------------------------  2409_2 ------------------------------------------------------------------------------------------------------------------------------------------------------  09WH45 ------------------------------------------------------------------------------------------------------------------------------------------------------  842 ------------------------------------------------------------------------------------------------------------------------------------------------------  Kale_A -------------------------------------------------------------------------------------------------------------------------------------------------ATGTT  Cauliflower_A -------------------------------------------------------------------------------------------------------------------------------------------------ATGTT  B90_1 ------------------------------------------------------------------------------------------------------------------------------------------------------  B90_2 ------------------------------------------------------------------------------------------------------------------------------------------------------  PurpleB ------------------------------------------------------------------------------------------------------------------------------------------------------    TO1000 GACAGATATTCTCAGGTCCTGTTGTCACTGTCAAAGTGTTCGAAGACAATGGCTTGATCCGTCAGTTCCTCGAGGAGAAAAGGTAACTGAATCTCATTCTTTAAAAACCTAAACTTGATTTGTAACTTTCACTACTTGCACAGAGATAAA  Kale_B GACAGATATTCTCAGGTCCTGTTGTCACTGTCAAAGTGTTCGAAGACAATGGCTTGATCCGTCAGTTCCTCGAGGAGAAAAGGTAACTGAATCTCATTCTTTAAAAACCTAAACTTGATTTGTAACTTTCACTACTTGCACAGAGATAAA  Cauliflower_B GACAGATATTCTCAGGTCCTGTTGTCACTGTCAAAGTGTTCGAAGACAATGGCTTGATCCGTCAGTTCCTCGAGGAGAAAAGGTAACTGAATCTCATTCTTTAAAAACCTAAACTTGATTTGTAACTTTCACTACTTGCACAGAGATAAA  154_1 ------------------------------------------------------------------------------------------------------------------------------------------------------  GreenB ------------------------------------------------------------------------------------------------------------------------------------------------------  154_2 ------------------------------------------------------------------------------------------------------------------------------------------------------  Broccoli GAAGAACAACTGCATGCCTGATAACGCTCTTCTCAGCACTCTCATCCACTGGCTTTGCAGAGACGGGAGAGTTACAGAAGCGAGGAAGTTGTTGGACGAGTTTGAGAAAGGCTCGATCCCTAGTCTCTTGACGTACAACACGTTGAT---  GreenA ------------------------------------------------------------------------------------------------------------------------------------------------------  2409_1 ------------------------------------------------------------------------------------------------------------------------------------------------------  2409_2 ------------------------------------------------------------------------------------------------------------------------------------------------------  09WH45 ------------------------------------------------------------------------------------------------------------------------------------------------------  842 ------------------------------------------------------------------------------------------------------------------------------------------------------  Kale_A GAAGAACAACTGCATGCCTGATAACGCTCTTCTCAGCACTCTCATCCACTGGCTTTGCAGAGACGGGAGAGTTACAGAAGCGAGGAAGTTGTTGGACGAGTTTGAGAAAGGCTCGATCCCTAGTCTCTTGACGTACAACACGTTGATCTC  Cauliflower_A GAAGAACAACTGCATGCCTGATAACGCTCTTCTCAGCACTCTCATCCACTGGCTTTGCAGAGACGGGAGAGTTACAGAAGCGAGGAAGTTGTTGGACGAGTTTGAGAAAGGCTCGATCCCTAGTCTCTTGACGTACAACACGTTGATCTC  B90_1 ------------------------------------------------------------------------------------------------------------------------------------------------------  B90_2 ------------------------------------------------------------------------------------------------------------------------------------------------------  PurpleB ------------------------------------------------------------------------------------------------------------------------------------------------------    TO1000 GATAATGACTATGACATGATTTCTTATTACACAAGCACATAGTTGTTACTTTGCTTAGAGTGTAATCTATCACGTTGCAAACGGGTGATAGTTGTTATTTTGCTTAGTTTTCAAATGTTTTGGATGATGTTATTAAGGAAACGGGAGAGT  Kale_B GATAATGACTATGACATGATTTCTTATTACACAAGCACATAGTTGTTACTTTGCTTAGAGTGTAATCTATCACGTTGCAAACGGGTGATAGTTGTTATTTTGCTTAGTTTTCAAATGTTTTGGATGATGTTATTAAGGAAACGGGAGAGT  Cauliflower_B GATAATGACTATGACATGATTTCTTATTACACAAGCACATAGTTGTTACTTTGCTTAGAGTGTAATCTATCACGTTGCAAACGGGTGATAGTTGTTATTTTGCTTAGTTTTCAAATGTTTTGGATGATGTTATTAAGGAAACGGGAGAGT  154_1 ------------------------------------------------------------------------------------------------------------------------------------------------------  GreenB ------------------------------------------------------------------------------------------------------------------------------------------------------  154_2 ------------------------------------------------------------------------------------------------------------------------------------------------------  Broccoli -----------AGAAGGGAGAGCTGACGGAGGCGGGTCGGTTATGGGACGACATGGTTGAGAGGAAGTGTAAGCCGAATGCTTTCACGTATAACGTTCTGATCGAAGGCCTGTGCAAGATGGGGAATGTTAAGGAAGGTGTCTTAAAGAG  GreenA ------------------------------------------------------------------------------------------------------------------------------------------------------  2409_1 ------------------------------------------------------------------------------------------------------------------------------------------------------  2409_2 ------------------------------------------------------------------------------------------------------------------------------------------------------  09WH45 ------------------------------------------------------------------------------------------------------------------------------------------------------  842 ------------------------------------------------------------------------------------------------------------------------------------------------------  Kale_A TGGGATGTGCGAGAAGGGAGAGCTGACGGAGGCGGGTCGGTTATGGGACGACATGGTTGAGAGGAAGTGTAAGCCGAATGCTTTCACGTATAACGTTCTGATCGAAGGCCTGTGCAAGATGGGGAATGTTAAGGAAGGTGTCTTAAAGAG  Cauliflower_A TGGGATGTGCGAGAAGGGAGAGCTGACGGAGGCGGGTCGGTTATGGGACGACATGGTTGAGAGGAAGTGTAAGCCGAATGCTTTCACGTATAACGTTCTGATCGAAGGCCTGTGCAAGATGGGGAATGTTAAGGAAGGTGTCTTAAAGAG  B90_1 -------------------------------------------------------------------------------------------ACGAAAGAAGGGGAATGTTAAGGAAGGTGTCTTAAAGAGATGCTGGAGAATGGTTGTTT  B90_2 -------------------------------------------------------------------------------------------ACGAAAGAAGGGGAATGTTAAGGAAGGTGTCTTAAAGAGATGCTGGAGAATGGTTGTTT  PurpleB ------------------------------------------------------------------------------------------------------------------------------------------------------    TO1000 ACTTGTGGAGGGAGCCAACGATGTGCAATACTCGGAGGCAACCCCGTGGTTCAAGCTCATTGTGAACGGATGCATCAGAGACGTTGATGAGATCAACGGTTGTGATATTGGAGTGAGAGCTTTGGCCTCTCATCCAATAAAGGCGAGCAA  Kale_B ACTTGTGGAGGGAGCCAACGATGTGCAATACTCGGAGGCAACCCCGTGGTTCAAGCTCATTGTGAACGGATGCATCAGAGACGTTGATGAGATCAACGGTTGTGATATTGGAGTGAGAGCTTTGGCCTCTCATCCAATAAAGGCGAGCAA  Cauliflower_B ACTTGTGGAGGGAGCCAACGATGTGCAATACTCGGAGGCAACCCCGTGGTTCAAGCTCATTGTGAACGGATGCATCAGAGACGTTGATGAGATCAACGGTTGTGATATTGGAGTGAGAGCTTTGGCCTCTCATCCAATAAAGGCGAGCAA  154_1 ----------------------------------------------------------------------------------ATGGCTGTTAAGAGCGGGAAAGTTGATAGAG--AATGTTGGGAGCTTTTCTTGAGGAAATTTGCTGGT  GreenB ----------------------------------------------------------------------------------ATGGCTGTTAAGAGCGGGAAAGTTGATAGAG--AATGTTGGGAGCTTTTCTTGAGGAAATTTGCTGGT  154_2 ----------------------------------------------------------------------------------ATGGCTGTTAAGAGCGGGAAAGTTGATAGAG--AATGTTGGGAGCTTTTCTTGAGGAAATTTGCTGGT  Broccoli ATGCTGGAGAATGGTTGTTTTCCGAATAAAACGAGGTTCTTGATATTGTTCGAGGGGTTGCAGGAGTCAGGGAAAGAAGAGGATGGCTGTTAAGAGCGGGAAAGTTGATAGAG--AATGTTGGGAGCTTTTCTTGAGGAAATTTGCTGGT  GreenA ----------------------------------------------------------------------------------ATGGCTGTTAAGAGCGGGAAAGTTGATAGAG--AATGTTGGGAGCTTTTCTTGAGGAAATTTGCTGGT  2409_1 ----------------------------------------------------------------------------------ATGGCTGTTAAGAGCGGGAAAGTTGATAGAG--AATGTTGGGAGCTTTTCTTGAGGAAATTTGCTGGT  2409_2 ----------------------------------------------------------------------------------ATGGCTGTTAAGAGCGGGAAAGTTGATAGAG--AATGTTGGGAGCTTTTCTTGAGGAAATTTGCTGGT  09WH45 ----------------------------------------------------------------------------------ATGGCTGTTAAGAGCGGGAAAGTTGATAGAG--AATGTTGGGAGCTTTTCTTGAGGAAATTTGCTGGT  842 ----------------------------------------------------------------------------------ATGGCTGTTAAGAGCGGGAAAGTTGATAGAG--AATGTTGGGAGCTTTTCTTGAGGAAATTTGCTGGT  Kale_A ATGCTGGAGAATGGTTGTTTTCCGAATAAAACGAGGTTCTTGATATTGTTCGAGGGGTTGCAGGAGTCAGGGAAAGAAGAGGATGGCTGTTAAGAGCGGGAAAGTTGATAGAG--AATGTTGGGAGCTTTTCTTGAGGAAATTTGCTGGT  Cauliflower_A ATGCTGGAGAATGGTTGTTTTCCGAATAAAACGAGGTTCTTGATATTGTTCGAGGGGTTGCAGGAGTCAGGGAAAGAAGAGGATGGCTGTTAAGAGCGGGAAAGTTGATAGAG--AATGTTGGGAGCTTTTCTTGAGGAAATTTGCTGGT  B90_1 TCCGAATAAAACGAGGTTCTTGATATTGTTCGAGGGGTTGCAGGAGTCAGGGAAAGAAGAGGATGGGGTTAAGATTGTGTCGATGGCTGTTAAGAGCGGGAAAGTTGATAGAG--AATGTTGGGAGCTTTTCTTGAGGAAATTTGCTGGT  B90_2 TCCGAATAAAACGAGGTTCTTGATATTGTTCGAGGGGTTGCAGGAGTCAGGGAAAGAAGAGGATGGGGTTAAGATTGTGTCGATGGCTGTTAAGAGCGGGAAAGTTGATAGAG--AATGTTGGGAGCTTTTCTTGAGGAAATTTGCTGGT  PurpleB ----------------------------------------------------------------------------------ATGGCTGTTAAGAGCGGGAAAGTTGATAGAG--AATGTTGGGAGCTTTTCTTGAGGAAATTTGCTGGT  * * ** * * *** * * * *** * *** * ** * * * *** *  TO1000 GAAAGGACTTGGAGAGCAATGAGTTCCAGTTAAT-ATTGCAGGGACTCGGATTTGTGATGGTGAATGGGTTTATGCTGATACTGATGGCATTCTCGTCTCTCAGACTGAGTTATCCGTTTAATACAAAAAAGCTTGTCTTCTTCCTTTTG  Kale_B GAAAGGACTTGGAGAGCAATGAGTTCCAGTTAAT-ATTGCAGGGACTCGGATTTGTGATGGTGAATGGGTTTATGCTGATACTGATGGCATTCTCGTCTCTCAGACTGAGTTATCCGTTTAATACAAAAAAGCTTGTCTTCTTCCTTTTG  Cauliflower_B GAAAGGACTTGGAGAGCAATGAGTTCCAGTTAAT-ATTGCAGGGACTCGGATTTGTGATGGTGAATGGGTTTATGCTGATACTGATGGCATTCTCGTCTCTCAGACTGAGTTATCCGTTTAATACAAAAAAGCTTGTCTTCTTCCTTTTG  154_1 GAGCTAGACAAAGGGGAGGTGGTTCTTAAAGAATTGTTGCAGGGACTCGGATTTGTGATGGTGAATGGGTTTATGCTGATACTGATGGCATTCTCGTCTCTCAGACTGAGTTATCCGTTTAATACAAAAAAGCTTGTCTTCTTCCTTTTG  GreenB GAGCTAGACAAAGGGGAGGTGGTTCTTAAAGAATTGTTGCAGGGACTCGGATTTGTGATGGTGAATGGGTTTATGCTGATACTGATGGCATTCTCGTCTCTCAGACTGAGTTATCCGTTTAATACAAAAAAGCTTGTCTTCTTCCTTTTG  154_2 GAGCTAGACAAAGGGGAGGTGGTTCTTAAAGAATTGTTGCAGGGACTCGGATTTGTGATGGTGAATGGGTTTATGCTGATACTGATGGCATTCTCGTCTCTCAGACTGAGTTATCCGTTTAATACAAAAAAGCTTGTCTTCTTCCTTTTG  Broccoli GAGCTAGACAAAGGGGAGGTGGTTCTTAAAGAATTGTTGCAGGGACTCGGATTTGTGATGGTGAATGGGTTTATGCTGATACTGATGGCATTCTCGTCTCTCAGACTGAGTTATCCGTTTAATACAAAAAAGCTTGTCTTCTTCCTTTTG  GreenA GAGCTAGACAAAGGGGAGGTGGTTCTTAAAGAATTGTTGCAGGGACTCGGATTTGTGATGGTGAATGGGTTTATGCTGATACTGATGGCATTCTCGTCTCTCAGACTGAGTTATCCGTTTAATACAAAAAAGCTTGTCTTCTTCCTTTTG  2409_1 GAGCTAGACAAAGGGGAGGTGGTTCTTAAAGAATTGTTGCAGGGACTCGGATTTGTGATGGTGAATGGGTTTATGCTGATACTGATGGCATTCTCGTCTCTCAGACTGAGTTATCCGTTTAATACAAAAAAGCTTGTCTTCTTCCTTTTG  2409_2 GAGCTAGACAAAGGGGAGGTGGTTCTTAAAGAATTGTTGCAGGGACTCGGATTTGTGATGGTGAATGGGTTTATGCTGATACTGATGGCATTCTCGTCTCTCAGACTGAGTTATCCGTTTAATACAAAAAAGCTTGTCTTCTTCCTTTTG  09WH45 GAGCTAGACAAAGGGGAGGTGGTTCTTAAAGAATTGTTGCAGGGACTCGGATTTGTGATGGTGAATGGGTTTATGCTGATACTGATGGCATTCTCGTCTCTCAGACTGAGTTATCCGTTTAATACAAAAAAGCTTGTCTTCTTCCTTTTG  842 GAGCTAGACAAAGGGGAGGTGGTTCTTAAAGAATTGTTGCAGGGACTCGGATTTGTGATGGTGAATGGGTTTATGCTGATACTGATGGCATTCTCGTCTCTCAGACTGAGTTATCCGTTTAATACAAAAAAGCTTGTCTTCTTCCTTTTG  Kale_A GAGCTAGACAAAGGGGAGGTGGTTCTTAAAGAATTGTTGCAGGGACTCGGATTTGTGATGGTGAATGGGTTTATGCTGATACTGATGGCATTCTCGTCTCTCAGACTGAGTTATCCGTTTAATACAAAAAAGCTTGTCTTCTTCCTTTTG  Cauliflower_A GAGCTAGACAAAGGGGAGGTGGTTCTTAAAGAATTGTTGCAGGGACTCGGATTTGTGATGGTGAATGGGTTTATGCTGATACTGATGGCATTCTCGTCTCTCAGACTGAGTTATCCGTTTAATACAAAAAAGCTTGTCTTCTTCCTTTTG  B90_1 GAGCTAGACAAAGGGGAGGTGGTTCTTAAAGAATTGTTGCAGGGACTCGGATTTGTGATGGTGAATGGGTTTATGCTGATACTGATGGCATTCTCGTCTCTCAGACTGAGTTATCCGTTTAATACAAAAAAGCTTGTCTTCTTCCTTTTG  B90_2 GAGCTAGACAAAGGGGAGGTGGTTCTTAAAGAATTGTTGCAGGGACTCGGATTTGTGATGGTGAATGGGTTTATGCTGATACTGATGGCATTCTCGTCTCTCAGACTGAGTTATCCGTTTAATACAAAAAAGCTTGTCTTCTTCCTTTTG  PurpleB GAGCTAGACAAAGGGGAGGTGGTTCTTAAAGAATTGTTGCAGGGACTCGGATTTGTGATGGTGAATGGGTTTATGCTGATACTGATGGCATTCTCGTCTCTCAGACTGAGTTATCCGTTTAATACAAAAAAGCTTGTCTTCTTCCTTTTG  ** * * ** * * *** ******************************************************************************************************************  TO1000 TTGTATTTGATCAGTTTCTAAGAACACATGAACTTGGTTCCCCCGATGATGTTTACATGAT-GCTACTGCTCTTTATCTGTTTTAGACCGGTTATGTTACAGTTTATACAATTTCCGGTTTATTACAGAGCTGAGAACACACCAAAAAAA  Kale_B TTGTATTTGATCAGTTTCTAAGAACACATGAACTTGGTTCCCCCGATGATGTTTACATGAT-GCTACTGCTCTTTATCTGTTTTAGACCGGTTATGTTACAGTTTATACAATTTCCGGTTTATTACAGAGCTGAGAACACACCAAAAAAA  Cauliflower_B TTGTATTTGATCAGTTTCTAAGAACACATGAACTTGGTTCCCCCGATGATGTTTACATGAT-GCTACTGCTCTTTATCTGTTTTAGACCGGTTATGTTACAGTTTATACAATTTCCGGTTTATTACAGAGCTGAGAACACACCAAAAAAA  154_1 TTGTATTTGATCAGTTTCTAAGAACACCTGAACTTGGTTCCCCCGATGATGTTTACATGAT-GCTACTGCTCTTTATCTGTTTTAGACCGGTTATGTTACAGTTTTTACAATTTCCGGTTTATTACAGAGCTGAGAACACAC--AAAAAG  GreenB TTGTATTTGATCAGTTTCTAAGAACACCTGAACTTGGTTCCCCCGATGATGTTTACATGAT-GCTACTGCTCTTTATCTGTTTTAGACCGGTTATGTTACAGTTTTTACAATTTCCGGTTTATTACAGAGCTGAGAACACAC--AAAAAG  154_2 TTGTATTTGATCAGTTTCTAAGAACACCTGAACTTGGTTCCCCCGATGATGTTTACATGAT-GCTACTGCTCTTTATCTGTTTTAGACCGGTTATGTTACAGTTTTTACAATTTCCGGTTTATTACAGAGCTGAGAACACAC--AAAAAG  Broccoli TTGTATTTGATCAGTTTCTAAGAACACCTGAACTTGGTTCCCCCGATGATGTTTACATGAT-GCTACTGCTCTTTATCTGTTTTAGACCGGTTATGTTACAGTTTTTACAATTTCCGGTTTATTACAGAGCTGAGAACACAC--AAAAAG  GreenA TTGTATTTGATCAGTTTCTAAGAACACCTGAACTTGGTTCCCCCGATGATGTTTACATGAT-GCTACTGCTCTTTATCTGTTTTAGACCGGTTATGTTACAGTTTTTACAATTTCCGGTTTATTACAGAGCTGAGAACACAC--AAAAAG  2409_1 TTGTATTTGATCAGTTTCTAAGAACACATGAACTTGGTTCCCCCGATGATGTTTACATGAT-GCTACTGCTCTTTATCTGTTTTAGACCGGTTATGTTACAGTTTTTACAATTTCCGGTTTATTACAGAGCTGAGAACACAC--AAAAAA  2409_2 TTGTATTTGATCAGTTTCTAAGAACACATGAACTTGGTTCCCCCGATGATGTTTACATGAT-GCTACTGCTCTTTATCTGTTTTAGACCGGTTATGTTACAGTTTTTACAATTTCCGGTTTATTACAGAGCTGAGAACACAC--AAAAAA  09WH45 TTGTATTTGATCAGTTTCTAAGAACACATGAACTTGGTTCCCCCGATGATGTTTACATGAT-GCTACTGCTCTTTATCTGTTTTAGACCGGTTATGTTACAGTTTTTACAATTTCCGGTTTATTACAGAGCTGAGAACACAC--AAAAAA  842 TTGTATTTGATCAGTTTCTAAGAACACATGAACTTGGTTCCCCCGATGATGTTTACATGAT-GCTACTGCTCTTTATCTGTTTTAGACCGGTTATGTTACAGTTTTTACAATTTCCGGTTTATTACAGAGCTGAGAACACAC--AAAAAA  Kale_A TTGTATTTGATCAGTTTCTAAGAACACCTGAACTTGGTTCCCCCGATGATGTTTACATGAT-GCTACTGCTCTTTATCTGTTTTAGACCGGTTATGTTACAGTTTTTACAATTTCCGGTTTATTACAGAGCTGAGAACACAC--AAAAAG  Cauliflower_A TTGTATTTGATCAGTTTCTAAGAACACCTGAACTTGGTTCCCCCGATGATGTTTACATGAT-GCTACTGCTCTTTATCTGTTTTAGACCGGTTATGTTACAGTTTTTACAATTTCCGGTTTATTACAGAGCTGAGAACACAC--AAAAAG  B90_1 TTGTATTTGATCAGTTTCTAAGAACCAGTG--------TTCAAAAATACGGCTTACGCGGCCGCCGATACGGTCACTATACGCTATGC-GGCTATGTAACG---TACGAAATTCATCGTATACGGCATTATATGCGGTTTGTT-AAAAAA  B90_2 TTGTATTTGATCAGTTTCTAAGAACCAGTG--------TTCAAAAATACGGCTTACGCGGCCGCCGATACGGTCACTATACGCTATGC-GGCTATGTAACG---TACGAAATTCATCGTATACGGCATTATATGCGGTTTGTT-AAAAAA  PurpleB TTGTATTTGATCAGTTTCTAAGAACCAGTG--------TTCAAAAATACGGCTTACGCGGCCGCCGATACGGTCACTATACGCTATGC-GGCTATGTAACG---TACGAAATTCATCGTATACGGCATTATATGCGGTTTGTT-AAAAAA  ************************* ** * * ** * **** * ** * * * * * ** * ** ***** ** * **** ** ** ** *****  TO1000 AAAAACAAGAATCTAATTACTAATTAATTTACTATGTAACTGGGCTAATAACGATGGGCTTTGACGGGTCCTATAATA-CATTTAATACGGTTGATATTGGGTAGGTGGTAGCTGCCTAGCTGGTAAGTGAGAGAGGAGAAGAAGAGGTG  Kale_B AAAAACAAGAATCTAATTACTAATTAATTTACTATGTAACTGGGCTAATAACGATGGGCTTTGACGGGTCCTATAATA-CATTTAATACGGTTGATATTGGGTAGGTGGTAGCTGCCTAGCTGGTAAGTGAGAGAGGAGAAGAAGAGGTG  Cauliflower_B AAAAACAAGAATCTAATTACTAATTAATTTACTATGTAACTGGGCTAATAACGATGGGCTTTGACGGGTCCTATAATA-CATTTAATACGGTTGATATTGGGTAGGTGGTAGCTGCCTAGCTGGTAAGTGAGAGAGGAGAAGAAGAGGTG  154_1 TAAAACAAGAATCTAATTACTAATTAATTTACTATGTAACTGGGCTAATAACGATGGGCTTTGACGGGTCCTATAATA-CATTTAATACGGTTGATATTGGGTAGGTGGTAGCTG--------GTAAGTGAGAGAGGAGAAGAAGAGGTG  GreenB TAAAACAAGAATCTAATTACTAATTAATTTACTATGTAACTGGGCTAATAACGATGGGCTTTGACGGGTCCTATAATA-CATTTAATACGGTTGATATTGGGTAGGTGGTAGCTG--------GTAAGTGAGAGAGGAGAAGAAGAGGTG  154_2 TAAAACAAGAATCTAATTACTAATTAATTTACTATGTAACTGGGCTAATAACGATGGGCTTTGACGGGTCCTATAATA-CATTTAATACGGTTGATATTGGGTAGGTGGTAGCTG--------GTAAGTGAGAGAGGAGAAGAAGAGGTG  Broccoli TAAAACAAGAATCTAATTACTAATTAATTTACTATGTAACTGGGCTAATAACGATGGGCTTTGACGGGTCCTATAATA-CATTTAATACGGTTGATATTGGGTAGGTGGTAGCTG--------GTAAGTGAGAGAGGAGAAGAAGAGGTG  GreenA TAAAACAAGAATCTAATTACTAATTAATTTACTATGTAACTGGGCTAATAACGATGGGCTTTGACGGGTCCTATAATA-CATTTAATACGGTTGATATTGGGTAGGTGGTAGCTG--------GTAAGTGAGAGAGGAGAAGAAGAGGTG  2409_1 AAAA-CAACAATCTAATTACTAATTAATTTACTATGTAACTGGGCTAATAACGATGGGCTTTGACGGGTCCTATAATA-CATTTAATACGGTTGATATTGGGTAGGTGGTAGCTG--------GTAAGTGAGAGAGGAGAAGAAGAGGTG  2409_2 AAAA-CAACAATCTAATTACTAATTAATTTACTATGTAACTGGGCTAATAACGATGGGCTTTGACGGGTCCTATAATA-CATTTAATACGGTTGATATTGGGTAGGTGGTAGCTG--------GTAAGTGAGAGAGGAGAAGAAGAGGTG  09WH45 AAA--CAACAATCTAATTACTAATTAATTTACTATGTAACTGGGCTAATAACGATGGGCTTTGACGGGTCCTATAATA-CATTTAATACGGTTGATATTGGGTAGGTGGTAGCTG--------GTAAGTGAGAGAGGAGAAGAAGAGGTG  842 AAAAACAACAATCTAATTACTAATTAATTTACTATGTAACTGGGCTAATAACGATGGGCTTTGACGGGTCCTATAATA-CATTTAATACGGTTGATATTGGGTAGGTGGTAGCTG--------GTAAGTGAGAGAGGAGAAGAAGAGGTG  Kale_A TAAAACAAGAATCTAATTACTAATTAATTTACTATGTAACTGGGCTAATAACGATGGGCTTTGACGGGTCCTATAATA-CATTTAATACGGTTGATATTGGGTAGGTGGTAGCTG--------GTAAGTGAGAGAGGAGAAGAAGAGGTG  Cauliflower_A TAAAACAAGAATCTAATTACTAATTAATTTACTATGTAACTGGGCTAATAACGATGGGCTTTGACGGGTCCTATAATA-CATTTAATACGGTTGATATTGGGTAGGTGGTAGCTG--------GTAAGTGAGAGAGGAGAAGAAGAGGTG  B90_1 CGGGCGTACATACGGTTTACGCGGACGTTTA-TACGGTGGTGGCGTCTAGGCGTTAGACATTGTTTTGTTTGAAAAGAATCTTTTCAAAAGATAAAGTTACGTAATTAAATGAAGTTGA----ATTAAAAAAAGTAAAAACTGAAAAACA  B90_2 CGGGCGTACATACGGTTTACGCGGACGTTTA-TACGGTGGTGGCGTCTAGGCGTTAGACATTGTTTTGTTTGAAAAGAATCTTTTCAAAAGATAAAGTTACGTAATTAAATGAAGTTGA----ATTAAAAAAAGTAAAAACTGAAAAACA  PurpleB CGGGCGTACATACGGTTTACGCGGACGTTTA-TACGGTGGTGGCGTCTAGGCGTTAGACATTGTTTTGTTTGAAAAGAATCTTTTCAAAAGATAAAGTTACGTAATTAAATGAAGTTGA----ATTAAAAAAAGTAAAAACTGAAAAACA  * * * **** **** ** * *** * ** * * * *** ** * ** * *** * * * * ** *** * * * * * * ** * * * *  TO1000 AATGATTAAACAAAATCCCTCGTACTCTAACCACCAGTCAATTCAACTGAATGCTCATGTTTC-TTCGATCTTATCTGTTTCCTGCACC-CACA--CTTTGTAGTTTGTCCCTTTC-TTTCAATTTATCGTGTCTCTCTCAATAATTTGG  Kale_B AATGATTAAACAAAATCCCTCGTACTCTAACCACCAGTCAATTCAACTGAATGCTCATGTTTC-TTCGATCTTATCTGTTTCCTGCACC-CACA--CTTTGTAGTTTGTCCCTTTC-TTTCAATTTATCGTGTCTCTCTCAATAATTTGG  Cauliflower_B AATGATTAAACAAAATCCCTCGTACTCTAACCACCAGTCAATTCAACTGAATGCTCATGTTTC-TTCGATCTTATCTGTTTCCTGCACC-CACA--CTTTGTAGTTTGTCCCTTTC-TTTCAATTTATCGTGTCTCTCTCAATAATTTGG  154_1 AATGATTAAACAAAATCCCTCGTACTCTAACCACCAGTCAATTCAACTGAATGCTCATGTTTC-TTCGATCTTATCTGTTTCCTGCACC-CACA--CTTTGTAGTTTGCCCCTTTC-TTTCAATTTATCGCGTCTCTCTCAATAATTTGG  GreenB AATGATTAAACAAAATCCCTCGTACTCTAACCACCAGTCAATTCAACTGAATGCTCATGTTTC-TTCGATCTTATCTGTTTCCTGCACC-CACA--CTTTGTAGTTTGCCCCTTTC-TTTCAATTTATCGCGTCTCTCTCAATAATTTGG  154_2 AATGATTAAACAAAATCCCTCGTACTCTAACCACCAGTCAATTCAACTGAATGCTCATGTTTC-TTCGATCTTATCTGTTTCCTGCACC-CACA--CTTTGTAGTTTGCCCCTTTC-TTTCAATTTATCGCGTCTCTCTCAATAATTTGG  Broccoli AATGATTAAACAAAATCCCTCGTACTCTAACCACCAGTCAATTCAACTGAATGCTCATGTTTC-TTCGATCTTATCTGTTTCCTGCACC-CACA--CTTTGTAGTTTGCCCCTTTC-TTTCAATTTATCGCGTCTCTCTCAATAATTTGG  GreenA AATGATTAAACAAAATCCCTCGTACTCTAACCACCAGTCAATTCAACTGAATGCTCATGTTTC-TTCGATCTTATCTGTTTCCTGCACC-CACA--CTTTGTAGTTTGCCCCTTTC-TTTCAATTTATCGCGTCTCTCTCAATAATTTGG  2409_1 AATGATTAAACAAAATCCCTCGTACTCTAACCACCAGTCAATTCAACTGAACGCTCATGTTTC-TTCGATCTTATCTGTTTCCTGCACC-CACA--CTTTGTAGTTTGTCCCTTTC-TTTCAATTTATCGCGTCTCTCTCAATAATTTGG  2409_2 AATGATTAAACAAAATCCCTCGTACTCTAACCACCAGTCAATTCAACTGAACGCTCATGTTTC-TTCGATCTTATCTGTTTCCTGCACC-CACA--CTTTGTAGTTTGTCCCTTTC-TTTCAATTTATCGCGTCTCTCTCAATAATTTGG  09WH45 AATGATTAAACAAAATCCCTCGTACTCTAACCACCAGTCAATTCAACTGAACGCTCATGTTTC-TTCGATCTTATCTGTTTCCTGCACC-CACA--CTTTGTAGTTTGTCCCTTTC-TTTCAATTTATCGCGTCTCTCTCAATAATTTGG  842 AATGATTAAACAAAATCCCTCGTACTCTAACCACCAGTCAATTCAACTGAACGCTCATGTTTC-TTCGATCTTATCTGTTTCCTGCACC-CACA--CTTTGTAGTTTGTCCCTTTC-TTTCAATTTATCGCGTCTCTCTCAATAATTTGG  Kale_A AATGATTAAACAAAATCCCTCGTACTCTAACCACCAGTCAATTCAACTGAATGCTCATGTTTC-TTCGATCTTATCTGTTTCCTGCACC-CACA--CTTTGTAGTTTGCCCCTTTC-TTTCAATTTATCGCGTCTCTCTCAATAATTTGG  Cauliflower_A AATGATTAAACAAAATCCCTCGTACTCTAACCACCAGTCAATTCAACTGAATGCTCATGTTTC-TTCGATCTTATCTGTTTCCTGCACC-CACA--CTTTGTAGTTTGCCCCTTTC-TTTCAATTTATCGCGTCTCTCTCAATAATTTGG  B90_1 TAAAACTACGTAGTTTAGCTAAAGTTTTAA--GTTGTTAACCCTAATTAGCAATTCACGTTCAATTCATTGTCTTCTAAAGTCTATACCACAGAGCCCTGCTAGGCTGCCACCTTCACTTTGCCTCTTTATCGCTTTCTTATCTACCAAA  B90_2 TAAAACTACGTAGTTTAGCTAAAGTTTTAA--GTTGTTAACCCTAATTAGCAATTCACGTTCAATTCATTGTCTTCTAAAGTCTATACCACAGAGCCCTGCTAGGCTGCCACCTTCACTTTGCCTCTTTATCGCTTTCTTATCTACCAAA  PurpleB TAAAACTACGTAGTTTAGCTAAAGTTTTAA--GTTGTTAACCCTAATTAGCAATTCACGTTCAATTCATTGTCTTCTAAAGTCTATACCACAGAGCCCTGCTAGGCTGCCACCTTCACTTTGCCTCTTTATCGCTTTCTTATCTACCAAA  * * ** * * ** * *** * * ** * *** *** *** * * *** ** *** ** * * * *** ** * * *** ** * * ** *** * *  TO1000 TATTTCT-CTTCAACTTCCGGATAATAGTCAGATATCTATATACCCAATTAATTTTGAGCCACTTACAAAATATCTTCTTATATTCATTATTTTATTTGCTGAAATATCTTTTTTTTTCCTATTTGTTGCTGAAATAATTGGACAAAATA  Kale_B TATTTCT-CTTCAACTTCCGGATAATAGTCAGATATCTATATACCCAATTAATTTTGAGCCACTTACAAAATATCTTCTTATATTCATTATTTTATTTGCTGAAATATCTTTTTTTTTCCTATTTGTTGCTGAAATAATTGGACAAAATA  Cauliflower_B TATTTCT-CTTCAACTTCCGGATAATAGTCAGATATCTATATACCCAATTAATTTTGAGCCACTTACAAAATATCTTCTTATATTCATTATTTTATTTGCTGAAATATCTTTTTTTTTCCTATTTGTTGCTGAAATAATTGGACAAAATA  154_1 TATTTCT-CTTCAACTTCCGGATATTAGTCAGATATCTATATACCCAATTAATTTTGAGCCACTTACAAAATATCTTCTTATATTCATTATTTTATTTGCTGAAATATCTTTTTTTTTCCTATTTGTTGCTGAAATAATTGGACAAAATA  GreenB TATTTCT-CTTCAACTTCCGGATATTAGTCAGATATCTATATACCCAATTAATTTTGAGCCACTTACAAAATATCTTCTTATATTCATTATTTTATTTGCTGAAATATCTTTTTTTTTCCTATTTGTTGCTGAAATAATTGGACAAAATA  154_2 TATTTCT-CTTCAACTTCCGGATATTAGTCAGATATCTATATACCCAATTAATTTTGAGCCACTTACAAAATATCTTCTTATATTCATTATTTTATTTGCTGAAATATCTTTTTTTTTCCTATTTGTTGCTGAAATAATTGGACAAAATA  Broccoli TATTTCT-CTTCAACTTCCGGATATTAGTCAGATATCTATATACCCAATTAATTTTGAGCCACTTACAAAATATCTTCTTATATTCATTATTTTATTTGCTGAAATATCTTTTTTTTTCCTATTTGTTGCTGAAATAATTGGACAAAATA  GreenA TATTTCT-CTTCAACTTCCGGATATTAGTCAGATATCTATATACCCAATTAATTTTGAGCCACTTACAAAATATCTTCTTATATTCATTATTTTATTTGCTGAAATATCTTTTTTTTTCCTATTTGTTGCTGAAATAATTGGACAAAATA  2409_1 TATTTCT-CTTCAACTTCCGGATATTAGTCAGATATCTATATACCCAATTAATTTTGAGCCACTTACAAAATATCTTCTTATATTCATTATTTTATTTGCTGAAATATCTTTTTTTTTCCTATTTGTTGCTGAAATAATTGGACAAAATA  2409_2 TATTTCT-CTTCAACTTCCGGATATTAGTCAGATATCTATATACCCAATTAATTTTGAGCCACTTACAAAATATCTTCTTATATTCATTATTTTATTTGCTGAAATATCTTTTTTTTTCCTATTTGTTGCTGAAATAATTGGACAAAATA  09WH45 TATTTCT-CTTCAACTTCCGGATATTAGTCAGATATCTATATACCCAATTAATTTTGAGCCACTTACAAAATATCTTCTTATATTCATTATTTTATTTGCTGAAATATCTTTTTTTTTCCTATTTGTTGCTGAAATAATTGGACAAAATA  842 TATTTCT-CTTCAACTTCCGGATATTAGTCAGATATCTATATACCCAATTAATTTTGAGCCACTTACAAAATATCTTCTTATATTCATTATTTTATTTGCTGAAATATCTTTTTTTTTCCTATTTGTTGCTGAAATAATTGGACAAAATA  Kale_A TATTTCT-CTTCAACTTCCGGATATTAGTCAGATATCTATATACCCAATTAATTTTGAGCCACTTACAAAATATCTTCTTATATTCATTATTTTATTTGCTGAAATATCTTTTTTTTTCCTATTTGTTGCTGAAATAATTGGACAAAATA  Cauliflower_A TATTTCT-CTTCAACTTCCGGATATTAGTCAGATATCTATATACCCAATTAATTTTGAGCCACTTACAAAATATCTTCTTATATTCATTATTTTATTTGCTGAAATATCTTTTTTTTTCCTATTTGTTGCTGAAATAATTGGACAAAATA  B90_1 GGTTTTTGTTTTGATTTATGTTCTCTTCTTTTTTGTTTCTCTGCACA--TGTTTTTATTTTATTT-TGAAGT-TTGCAATAGTTCTAAAGTACGATCAGTTTTTCTTACTAGTTACTAAAGATTTGTTTTTGACTT--TTTTCCAAAAAC  B90_2 GGTTTTTGTTTTGATTTATGTTCTCTTCTTTTTTGTTTCTCTGCACA--TGTTTTTATTTTATTT-TGAAGT-TTGCAATAGTTCTAAAGTACGATCAGTTTTTCTTACTAGTTACTAAAGATTTGTTTTTGACTT--TTTTCCAAAAAC  PurpleB GGTTTTTGTTTTGATTTATGTTCTCTTCTTTTTTGTTTCTCTGCACA--TGTTTTTATTTTATTT-TGAAGT-TTGCAATAGTTCTAAAGTACGATCAGTTTTTCTTACTAGTTACTAAAGATTTGTTTTTGACTT--TTTTCCAAAAAC  *** * ** * ** * * * * * * * * * ** * **** * ** ** * * ** * * * ** * * * ** ** * ******* *** * ** *****    TO1000 TGTACGAGACTAATGAACTGATTTTGTAATTCCGGATATATTTCTACCAATTGTATTTATTTTGCCAGTTCACAATTATTTTTTGTTATATAATAAATTTTTATTCACTGGGTATAATTTTTTTATCAATTCATAATTTTAACAGATATT  Kale_B TGTACGAGACTAATGAACTGATTTTGTAATTCCGGATATATTTCTACCAATTGTATTTATTTTGCCAGTTCACAATTATTTTTTGTTATATAATAAATTTTTATTCACTGGGTATAATTTTTTTATCAATTCATAATTTTAACAGATATT  Cauliflower_B TGTACGAGACTAATGAACTGATTTTGTAATTCCGGATATATTTCTACCAATTGTATTTATTTTGCCAGTTCACAATTATTTTTTGTTATATAATAAATTTTTATTCACTGGGTATAATTTTTTTATCAATTCATAATTTTAACAGATATT  154_1 TGTACGAGACTAATGAACTGATTTTGTAATTCCGGATATATTTCTACCAATTGTATTTATTTTGCCAGTTCACAATTATTTTTTGTTATATAATAAATTTTTATTCACTGGGTATAATTTTTTTATCAATTCATAATTTTAACAGATATT  GreenB TGTACGAGACTAATGAACTGATTTTGTAATTCCGGATATATTTCTACCAATTGTATTTATTTTGCCAGTTCACAATTATTTTTTGTTATATAATAAATTTTTATTCACTGGGTATAATTTTTTTATCAATTCATAATTTTAACAGATATT  154_2 TGTACGAGACTAATGAACTGATTTTGTAATTCCGGATATATTTCTACCAATTGTATTTATTTTGCCAGTTCACAATTATTTTTTGTTATATAATAAATTTTTATTCACTGGGTATAATTTTTTTATCAATTCATAATTTTAACAGATATT  Broccoli TGTACGAGACTAATGAACTGATTTTGTAATTCCGGATATATTTCTACCAATTGTATTTATTTTGCCAGTTCACAATTATTTTTTGTTATATAATAAATTTTTATTCACTGGGTATAATTTTTTTATCAATTCATAATTTTAACAGATATT  GreenA TGTACGAGACTAATGAACTGATTTTGTAATTCCGGATATATTTCTACCAATTGTATTTATTTTGCCAGTTCACAATTATTTTTTGTTATATAATAAATTTTTATTCACTGGGTATAATTTTTTTATCAATTCATAATTTTAACAGATATT  2409_1 TGTACGAGACTAATGAACTGATTTTGTAATTCCGGATATATTTCTACCAATTGTATTTATTTTGCCAGTTCACAATTATTTTTTGTTATATAATAAATTTTTATTCACTGGGTATAATTTTTTTATCAATTCATAATTTTAACAGATATT  2409_2 TGTACGAGACTAATGAACTGATTTTGTAATTCCGGATATATTTCTACCAATTGTATTTATTTTGCCAGTTCACAATTATTTTTTGTTATATAATAAATTTTTATTCACTGGGTATAATTTTTTTATCAATTCATAATTTTAACAGATATT  09WH45 TGTACGAGACTAATGAACTGATTTTGTAATTCCGGATATATTTCTACCAATTGTATTTATTTTGCCAGTTCACAATTATTTTTTGTTATATAATAAATTTTTATTCACTGGGTATAATTTTTTTATCAATTCATAATTTTAACAGATATT  842 TGTACGAGACTAATGAACTGATTTTGTAATTCCGGATATATTTCTACCAATTGTATTTATTTTGCCAGTTCACAATTATTTTTTGTTATATAATAAATTTTTATTCACTGGGTATAATTTTTTTATCAATTCATAATTTTAACAGATATT  Kale_A TGTACGAGACTAATGAACTGATTTTGTAATTCCGGATATATTTCTACCAATTGTATTTATTTTGCCAGTTCACAATTATTTTTTGTTATATAATAAATTTTTATTCACTGGGTATAATTTTTTTATCAATTCATAATTTTAACAGATATT  Cauliflower_A TGTACGAGACTAATGAACTGATTTTGTAATTCCGGATATATTTCTACCAATTGTATTTATTTTGCCAGTTCACAATTATTTTTTGTTATATAATAAATTTTTATTCACTGGGTATAATTTTTTTATCAATTCATAATTTTAACAGATATT  B90_1 TGCAGAAAATCATTCAAGAGATTTTGCCATTTTGG-TATGGCTGGATCA-----------TCTGCAAATTCA-----AATATGAACCAAACAGGAACTTCAGAAACACCAGCTTTGA----------AA---AGAAATTCAAAGGATGTT  B90_2 TGCAGAAAATCATTCAAGAGATTTTGCCATTTTGG-TATGGCTGGATCA-----------TCTGCAAATTCA-----AATATGAACCAAACAGGAACTTCAGAAACACCAGCTTTGA----------AA---AGAAATTCAAAGGATGTT  PurpleB TGCAGAAAATCATTCAAGAGATTTTGCCATTTTGG-TATGGCTGGATCA-----------TCTGCAAATTCA-----AATATGAACCAAACAGGAACTTCAGAAACACCAGCTTTGA----------AA---A-AAATTCAAAGGATGTT  ** * * * * * ** ******* *** ** *** * * ** * *** * **** * * * * * * ** ** * *** * * * * ** * ** ** ** *** **  TO1000 TTGTATTAATATTTATTGTTAT-TAATAATTTTTTTTTT--CAAAACGGATAACTGATTGAAT--AAATTTATATTGGTGTAAAGTTCCTAGTAGGGGTGGGCACTTTACCCAATATCCGAAGCGGCATCCGAATCCGATCCGAAAAATT  Kale_B TTGTATTAATATTTATTGTTAT-TAATAATTTTTTTTTT--CAAAACGGATAACTGATTGAAT--AAATTTATATTGGTGTAAAGTTCCTAGTAGGGGTGGGCACTTTACCCAATATCCGAAGCGGCATCCGAATCCGATCCGAAAAATT  Cauliflower_B TTGTATTAATATTTATTGTTAT-TAATAATTTTTTTTTT--CAAAACGGATAACTGATTGAAT--AAATTTATATTGGTGTAAAGTTCCTAGTAGGGGTGGGCACTTTACCCAATATCCGAAGCGGCATCCGAATCCGATCCGAAAAATT  154_1 TTGTATTAATATTTATTGTTAT-TAAAATTTTTTTTTTT--CAAAACGGATAACTGATTGAAT--AAATTTATATTGGTGTAAAGTTCCTAGTAGGGGTGGGCACTTTACCCGATATCCGAAGCGGAATCCGAATCCGATCCGAAAAATT  GreenB TTGTATTAATATTTATTGTTAT-TAATATTTTTTTTTTT--CAAAACGGATAACTGATTGAAT--AAATTTATATTGGTGTAAAGTTCCTAGTAGGGGTGGGCACTTTACCCGATATCCGAAGCGGAATCCGAATCCGATCCGAAAAATT  154_2 TTGTATTAATATTTATTGTTAT-TAAAATTTTTTTTTTT--CAAAACGGATAACTGATTGAAT--AAATTTATATTGGTGTAAAGTTCCTAGTAGGGGTGGGCACTTTACCCGATATCCGAAGCGGAATCCGAATCCGATCCGAAAAATT  Broccoli TTGTATTAATATTTATTGTTAT-TAATATTTTTTTTTTT--CAAAACGGATAACTGATTGAAT--AAATTTATATTGGTGTAAAGTTCCTAGTAGGGGTGGGCACTTTACCCGATATCCGAAGCGGAATCCGAATCCGATCCGAAAAATT  GreenA TTGTATTAATATTTATTGTTAT-TAATATTTTTTTTTTT--CAAAACGGATAACTGATTGAAT--AAATTTATATTGGTGTAAAGTTCCTAGTAGGGGTGGGCACTTTACCCGATATCCGAAGCGGAATCCGAATCCGATCCGAAAAATT  2409_1 TTGTATTAATATTTATTGTTAT-TAATATTTTTTTTTTTT-CAAAACGGATAACTGATTGAAT--AAATTTATATTGGTGTAAAGTTCCTAGTAGGGGTGGGCACTTTACCCGATATCCGAAGCGGCATCCGAATCCGATCCGAAAAATT  2409_2 TTGTATTAATATTTATTGTTAT-TAATATTTTTTTTTTTT-CAAAACGGATAACTGATTGAAT--AAATTTATATTGGTGTAAAGTTCCTAGTAGGGGTGGGCACTTTACCCGATATCCGAAGCGGCATCCGAATCCGATCCGAAAAATT  09WH45 TTGTATTAATATTTATTGTTAT-TAATATTTTTTTTTTTT-CAAAACGGATAACTGATTGAAT--AAATTTATATTGGTGTAAAGTTCCTAGTAGGGGTGGGCACTTTACCCGATATCCGAAGCGGCATCCGAATCCGATCCGAAAAATT  842 TTGTATTAATATTTATTGTTAT-TAATATTTTTTTTTTTT-CAAAACGGATAACTGATTGAAT--AAATTTATATTGGTGTAAAGTTCCTAGTAGGGGTGGGCACTTTACCCGATATCCGAAGCGGCATCCGAATCCGATCCGAAAAATT  Kale_A TTGTATTAATATTTATTGTTAT-TAATATTTTTTTTTTT--CAAAACGGATAACTGATTGAAT--AAATTTATATTGGTGTAAAGTTCCTAGTAGGGGTGGGCACTTTACCCGATATCCGAAGCGGAATCCGAATCCGATCCGAAAAATT  Cauliflower_A TTGTATTAATATTTATTGTTAT-TAATATTTTTTTTTTT--CAAAACGGATAACTGATTGAAT--AAATTTATATTGGTGTAAAGTTCCTAGTAGGGGTGGGCACTTTACCCGATATCCGAAGCGGAATCCGAATCCGATCCGAAAAATT  B90_1 GCTTGGGAATATGGGATGTTGTGTAACCCAAGTAATCCAGATAAGGTGAAGTGTAAACTATGTGGAAAATTATTTTCTGGTGGTGCATTTAGGATTAAAGAGCATATTGCC-AAGATTCCAGGAAATGTTTCTGCTTGTCC------ACT  B90_2 GCTTGGGAATATGGGATGTTGTGTAACCCAAGTAATCCAGATAAGGTGAAGTGTAAACTATGTGGAAAATTATTTTCTGGTGGTGCATTTAGGATTAAAGAGCATATTGCC-AAGATTCCAGGAAATGTTTCTGCTTGTCC------ACT  PurpleB GCTTGGGAATATGGGATGTTGTGTAACCCAAGTAATCCAGATAAGGTGAAGTGTAAACTATGTGGAAAATTATTTTCTGGTGGTGCATTTAGGATTAAAGAGCATATTGCC-AAGATTCCAGGAAATGTTTCTGCTTGTCC------ACT  * ***** **** * *** * * ** * * * * * *** **** ** ** * *** * * *** ** ** * ** * * * * * * * *  TO1000 CGAACCGAAATCCGAACCGAAGTAGCAGAATATCCGAACGGGTATTAAATTAGGAGAGATTGGATATCCGAACCCGAACGGGTAATATCCAAACCCGAATGGATATCCAAAAATAACCGAACATACGTATAATTAACCTTATATTTCTAG  Kale_B CGAACCGAAATCCGAACCGAAGTAGCAGAATATCCGAACGGGTATTAAATTAGGAGAGATTGGATATCCGAACCCGAACGGGTAATATCCAAACCCGAATGGATATCCAAAAATAACCGAACATACGTATAATTAACCTTATATTTCTAG  Cauliflower_B CGAACCGAAATCCGAACCGAAGTAGCAGAATATCCGAACGGGTATTAAATTAGGAGAGATTGGATATCCGAACCCGAACGGGTAATATCCAAACCCGAATGGATATCCAAAAATAACCGAACATACGTATAATTAACCTTATATTTCTAG  154_1 CGAACCGAAATCCGAACCGAAGTAGCAGAATATCCGAACGGGTATTAAATTAGGAGAGATTGGATATCCGAACCCGAACGGGTAATATCCAAACCCGAATGGATATCCGAAAATAACTGAACATACGTATAATTAACTTTATATTTCTAG  GreenB CGAACCGAAATCCGAACCGAAGTAGCAGAATATCCGAACGGGTATTAAATTAGGAGAGATTGGATATCCGAACCCGAACGGGTAATATCCAAACCCGAATGGATATCCGAAAATAACTGAACATACGTATAATTAACTTTATATTTCTAG  154_2 CGAACCGAAATCCGAACCGAAGTAGCAGAATATCCGAACGGGTATTAAATTAGGAGAGATTGGATATCCGAACCCGAACGGGTAATATCCAAACCCGAATGGATATCCGAAAATAACTGAACATACGTATAATTAACTTTATATTTCTAG  Broccoli CGAACCGAAATCCGAACCGAAGTAGCAGAATATCCGAACGGGTATTAAATTAGGAGAGATTGGATATCCGAACCCGAACGGGTAATATCCAAACCCGAATGGATATCCGAAAATAACTGAACATACGTATAATTAACTTTATATTTCTAG  GreenA CGAACCGAAATCCGAACCGAAGTAGCAGAATATCCGAACGGGTATTAAATTAGGAGAGATTGGATATCCGAACCCGAACGGGTAATATCCAAACCCGAATGGATATCCGAAAATAACTGAACATACGTATAATTAACTTTATATTTCTAG  2409_1 CGAACCGAAATCCGAACCGAAGTAGCAGAATATCCGAACGGCTATTAAATTAGGAGAGATTGGATATCCGAACCCGAACGGGTAATATCCAAACCCGAATGGATATCCGAAAATAACCGAACATACGTATAATTAACTTTATATTTCTAG  2409_2 CGAACCGAAATCCGAACCGAAGTAGCAGAATATCCGAACGGCTATTAAATTAGGAGAGATTGGATATCCGAACCCGAACGGGTAATATCCAAACCCGAATGGATATCCGAAAATAACCGAACATACGTATAATTAACTTTATATTTCTAG  09WH45 CGAACCGAAATCCGAACCGAAGTAGCAGAATATCCGAACGGCTATTAAATTAGGAGAGATTGGATATCCGAACCCGAACGGGTAATATCCAAACCCGAATGGATATCCGAAAATAACCGAACATACGTATAATTAACTTTATATTTCTAG  842 CGAACCGAAATCCGAACCGAAGTAGCAGAATATCCGAACGGCTATTAAATTAGGAGAGATTGGATATCCGAACCCGAACGGGTAATATCCAAACCCGAATGGATATCCGAAAATAACCGAACATACGTATAATTAACTTTATATTTCTAG  Kale_A CGAACCGAAATCCGAACCGAAGTAGCAGAATATCCGAACGGGTATTAAATTAGGAGAGATTGGATATCCGAACCCGAACGGGTAATATCCAAACCCGAATGGATATCCGAAAATAACTGAACATACGTATAATTAACTTTATATTTCTAG  Cauliflower_A CGAACCGAAATCCGAACCGAAGTAGCAGAATATCCGAACGGGTATTAAATTAGGAGAGATTGGATATCCGAACCCGAACGGGTAATATCCAAACCCGAATGGATATCCGAAAATAACTGAACATACGTATAATTAACTTTATATTTCTAG  B90_1 ATCAACAAAAGATGATCAAGAAAAGTGCAA------AAATGCTATTGATGAAGCTAAGAATGGCAAAGAGAATTTTGTCATTGACTACT--AGTTCCTCGGGATGT---GAAAGAGCTTGGAGTTCTTTTGAAGGGGTAATTTTTATTAG  B90_2 ATCAACAAAAGATGATCAAGAAAAGTGCAA------AAATGCTATTGATGAAGCTAAGAATGGCAAAGAGAATTTTGTCATTGACTACT--AGTTCCTCGGGATGT---GAAAGAGCTTGGAGTTCTTTTGAAGGGGTAATTTTTATTAG  PurpleB ATCAACAAAAGATGATCAAGAAAAGTGCAA------AAATGCTATTGATGAAGCTAAGAATGGCAAAGAGAATTTTGTCATTGACTACT--AGTTCCTCGGGATGT---GAAAGAGCTTGGAGTTCTTTTGAAGGGGTAATTTTTATTAG  * * *** ** * * ** ** ** * **** * ** *** *** * *** * * ** * * **** * *** * * * * * * * * ** ***  TO1000 TTTACATCTCTCATTTATATATAATATTTATATTGATACTACACATATTTTAAGTTCATATGATATACATACAATTACGGAAAAAATGATTTGCTACTCACTTAAAATGCATGTCAAGCTTTTTATTTCAAGAATTAACAAAAACTTACA  Kale_B TTTACATCTCTCATTTATATATAATATTTATATTGATACTACACATATTTTAAGTTCATATGATATACATACAATTACGGAAAAAATGATTTGCTACTCACTTAAAATGCATGTCAAGCTTTTTATTTCAAGAATTAACAAAAACTTACA  Cauliflower_B TTTACATCTCTCATTTATATATAATATTTATATTGATACTACACATATTTTAAGTTCATATGATATACATACAATTACGGAAAAAATGATTTGCTACTCACTTAAAATGCATGTCAAGCTTTTTATTTCAAGAATTAACAAAAACTTACA  154_1 TTTACATCTCTCATTTATATAGAATATTTATATTGATACTACACATATTTTAAGTTCATATGATATACATACAATTACGGAAAAAATGATTTGCTACTCACTTAAAATGCATTTCAAGCTTTTTATTTCAAGAATTAACAAAAACTTACA  GreenB TTTACATCTCTCATTTATATAGAATATTTATATTGATACTACACATATTTTAAGTTCATATGATATACATACAATTACGGAAAAAATGATTTGCTACTCACTTAAAATGCATTTCAAGCTTTTTATTTCAAGAATTAACAAAAACTTACA  154_2 TTTACATCTCTCATTTATATAGAATATTTATATTGATACTACACATATTTTAAGTTCATATGATATACATACAATTACGGAAAAAATGATTTGCTACTCACTTAAAATGCATTTCAAGCTTTTTATTTCAAGAATTAACAAAAACTTACA  Broccoli TTTACATCTCTCATTTATATAGAATATTTATATTGATACTACACATATTTTAAGTTCATATGATATACATACAATTACGGAAAAAATGATTTGCTACTCACTTAAAATGCATTTCAAGCTTTTTATTTCAAGAATTAACAAAAACTTACA  GreenA TTTACATCTCTCATTTATATAGAATATTTATATTGATACTACACATATTTTAAGTTCATATGATATACATACAATTACGGAAAAAATGATTTGCTACTCACTTAAAATGCATTTCAAGCTTTTTATTTCAAGAATTAACAAAAACTTACA  2409_1 TTTACATCTCTCATTTATATAGAATATTTATATTGATACTACACATATTTTAAGTTCATATGATATACATACAATTACGGAAAAAATGATTTGCTACTCACTTAAAATGCATGTCAAACTTTTTATTTCAAGAATTAACAAAAACTTACA  2409_2 TTTACATCTCTCATTTATATAGAATATTTATATTGATACTACACATATTTTAAGTTCATATGATATACATACAATTACGGAAAAAATGATTTGCTACTCACTTAAAATGCATGTCAAACTTTTTATTTCAAGAATTAACAAAAACTTACA  09WH45 TTTACATCTCTCATTTATATAGAATATTTATATTGATACTACACATATTTTAAGTTCATATGATATACATACAATTACGGAAAAAATGATTTGCTACTCACTTAAAATGCATGTCAAACTTTTTATTTCAAGAATTAACAAAAACTTACA  842 TTTACATCTCTCATTTATATAGAATATTTATATTGATACTACACATATTTTAAGTTCATATGATATACATACAATTACGGAAAAAATGATTTGCTACTCACTTAAAATGCATGTCAAACTTTTTATTTCAAGAATTAACAAAAACTTACA  Kale_A TTTACATCTCTCATTTATATAGAATATTTATATTGATACTACACATATTTTAAGTTCATATGATATACATACAATTACGGAAAAAATGATTTGCTACTCACTTAAAATGCATTTCAAGCTTTTTATTTCAAGAATTAACAAAAACTTACA  Cauliflower_A TTTACATCTCTCATTTATATAGAATATTTATATTGATACTACACATATTTTAAGTTCATATGATATACATACAATTACGGAAAAAATGATTTGCTACTCACTTAAAATGCATTTCAAGCTTTTTATTTCAAGAATTAACAAAAACTTACA  B90_1 TCTATGATTCT---------AAAATCTTTTCATATGTATTG---ATGATTTTTGTTTAAAAAATGCAGATACA-TTAT-----------TTTAGTACTAATAT------TATTTTA----TTTCATATCAATCATTAATAATATATATCA  B90_2 TCTATGATTCT---------AAAATCTTTTCATATGTATTG---ATGATTTTTGTTTAAAAAATGCAGATACA-TTAT-----------TTTAGTACTAATAT------TATTTTA----TTTCATATCAATCATTAATAATATATATCA  PurpleB TCTATGATTCT---------AAAATCTTTTCATATGTATTG---ATGATTTTTGTTTAAAAAATGCAGATACA-TTAT-----------TTTAGTACTAATAT------TATTTTA----TTTCATATCAATCATTAATAATATATATCA  * ** *** * *** *** ** ** * ** *** *** * * ** * ***** *** *** **** * * ** * * *** ** **** ***** ** * * **  TO1000 CCAAAAATTTAAAAACAATAACCAAATTAATGTCTTTTTAGTTTCAAAATGTTATGTCGAAATCTATTAACCATTCAATCTATTAAAAATAAAAAAATTAGTTAAGTGAAAGTTATATTTTTAAATACAAGAAATTTGAGAAATGAAAAT  Kale_B CCAAAAATTTAAAAACAATAACCAAATTAATGTCTTTTTAGTTTCAAAATGTTATGTCGAAATCTATTAACCATTCAATCTATTAAAAATAAAAAAATTAGTTAAGTGAAAGTTATATTTTTAAATACAAGAAATTTGAGAAATGAAAAT  Cauliflower_B CCAAAAATTTAAAAACAATAACCAAATTAATGTCTTTTTAGTTTCAAAATGTTATGTCGAAATCTATTAACCATTCAATCTATTAAAAATAAAAAAATTAGTTAAGTGAAAGTTATATTTTTAAATACAAGAAATTTGAGAAATGAAAAT  154_1 TCAAAAATTTAAAAACAATAACCAAATTAATGTCTTTTTAGTTTCAAAATGTTATGTCGAAATCTATTAACCATTCAATCTATTAAAAATAAAAAAATTAGTTAAGTGAAAGTTATATTTTTAAATACAAGAAATTTGAGAAATGAAAAT  GreenB TCAAAAATTTAAAAACAATAACCAAATTAATGTCTTTTTAGTTTCAAAATGTTATGTCGAAATCTATTAACCATTCAATCTATTAAAAATAAAAAAATTAGTTAAGTGAAAGTTATATTTTTAAATACAAGAAATTTGAGAAATGAAAAT  154_2 TCAAAAATTTAAAAACAATAACCAAATTAATGTCTTTTTAGTTTCAAAATGTTATGTCGAAATCTATTAACCATTCAATCTATTAAAAATAAAAAAATTAGTTAAGTGAAAGTTATATTTTTAAATACAAGAAATTTGAGAAATGAAAAT  Broccoli TCAAAAATTTAAAAACAATAACCAAATTAATGTCTTTTTAGTTTCAAAATGTTATGTCGAAATCTATTAACCATTCAATCTATTAAAAATAAAAAAATTAGTTAAGTGAAAGTTATATTTTTAAATACAAGAAATTTGAGAAATGAAAAT  GreenA TCAAAAATTTAAAAACAATAACCAAATTAATGTCTTTTTAGTTTCAAAATGTTATGTCGAAATCTATTAACCATTCAATCTATTAAAAATAAAAAAATTAGTTAAGTGAAAGTTATATTTTTAAATACAAGAAATTTGAGAAATGAAAAT  2409_1 TCAAAAATTTAAAAACAATAACCAAATTAATGTCTTTTTAGTTTCAAAATGTTATGTCGAAATCTATTAACCATTCAATCTATTAAAAATAAAAAAATTAGTTAAGTGAAAGTTATATTTTTAAATACAAGAAATTTGAGAAATGAAAAT  2409_2 TCAAAAATTTAAAAACAATAACCAAATTAATGTCTTTTTAGTTTCAAAATGTTATGTCGAAATCTATTAACCATTCAATCTATTAAAAATAAAAAAATTAGTTAAGTGAAAGTTATATTTTTAAATACAAGAAATTTGAGAAATGAAAAT  09WH45 TCAAAAATTTAAAAACAATAACCAAATTAATGTCTTTTTAGTTTCAAAATGTTATGTCGAAATCTATTAACCATTCAATCTATTAAAAATAAAAAAATTAGTTAAGTGAAAGTTATATTTTTAAATACAAGAAATTTGAGAAATGAAAAT  842 TCAAAAATTTAAAAACAATAACCAAATTAATGTCTTTTTAGTTTCAAAATGTTATGTCGAAATCTATTAACCATTCAATCTATTAAAAATAAAAAAATTAGTTAAGTGAAAGTTATATTTTTAAATACAAGAAATTTGAGAAATGAAAAT  Kale_A TCAAAAATTTAAAAACAATAACCAAATTAATGTCTTTTTAGTTTCAAAATGTTATGTCGAAATCTATTAACCATTCAATCTATTAAAAATAAAAAAATTAGTTAAGTGAAAGTTATATTTTTAAATACAAGAAATTTGAGAAATGAAAAT  Cauliflower_A TCAAAAATTTAAAAACAATAACCAAATTAATGTCTTTTTAGTTTCAAAATGTTATGTCGAAATCTATTAACCATTCAATCTATTAAAAATAAAAAAATTAGTTAAGTGAAAGTTATATTTTTAAATACAAGAAATTTGAGAAATGAAAAT  B90_1 TTAATCATTAATATATA-TATATATATATATATATAT------CCAG-----TCCGTTTAGGCC------CCGTTTAGGCGTCCGCGTATACGGCTAGGCGCTAGGCG----CTACGTCACCGCACA----------GAGAGCGCATAGC  B90_2 TTAATCATTAATATATA-TATATATATATATATATATATATATCCAG-----TCCGTTTAGGCC------CCGTTTAGGCGTCCGCGTATACGGCTAGGCGCTAGGCG----CTACGTCACCGCACA----------GAGAGCGCATAGC  PurpleB TTAATCATTAATATATA-TATATATATATATATATATATAT--CCAG-----TCCGTTTAGGCC------CCGTTTAGGCGTCCGCGTATACGGCTAGGCGCTAGGCG----CTACGTCACCGCACA----------GAGAGCGCATAGC  ** *** * * * * ** * ** ** * * * ** * ** * * ** ** * * *** * * ** * * ** * * * **** * *      TO1000 TTAATTTTGTTTTTTCAAAATCTAAATATTCGAACTCGATCCGAAATAACCGAACCCGAACTAAAAATATCCGAACCCGACCCGAAGTACAGAAATACCCGAACGGGTTCTACACCTCTATAACTGAAATATCCAAAAATCTGAAATACC  Kale_B TTAATTTTGTTTTTTCAAAATCTAAATATTCGAACTCGATCCGAAATAACCGAACCCGAACTAAAAATATCCGAACCCGACCCGAAGTACAGAAATACCCGAACGGGTTCTACACCTCTATAACTGAAATATCCAAAAATCTGAAATACC  Cauliflower_B TTAATTTTGTTTTTTCAAAATCTAAATATTCGAACTCGATCCGAAATAACCGAACCCGAACTAAAAATATCCGAACCCGACCCGAAGTACAGAAATACCCGAACGGGTTCTACACCTCTATAACTGAAATATCCAAAAATCTGAAATACC  154_1 TTAATTTTGTTTTTTCAAAATCTAAATATCCGAACTCGATCCGAAATAACCGAACCCGAACTAAAAATATCCGAACCCGACCCGAAGTACAGAAATACCCGAACGGGTTCTACACCTCTATAACTGAAATATCCGAAAATCTGAAATACC  GreenB TTAATTTTGTTTTTTCAAAATCTAAATATCCGAACTCGATCCGAAATAACCGAACCCGAACTAAAAATATCCGAACCCGACCCGAAGTACAGAAATACCCGAACGGGTTCTACACCTCTATAACTGAAATATCCGAAAATCTGAAATACC  154_2 TTAATTTTGTTTTTTCAAAATCTAAATATCCGAACTCGATCCGAAATAACCGAACCCGAACTAAAAATATCCGAACCCGACCCGAAGTACAGAAATACCCGAACGGGTTCTACACCTCTATAACTGAAATATCCGAAAATCTGAAATACC  Broccoli TTAATTTTGTTTTTTCAAAATCTAAATATCCGAACTCGATCCGAAATAACCGAACCCGAACTAAAAATATCCGAACCCGACCCGAAGTACAGAAATACCCGAACGGGTTCTACACCTCTATAACTGAAATATCCGAAAATCTGAAATACC  GreenA TTAATTTTGTTTTTTCAAAATCTAAATATCCGAACTCGATCCGAAATAACCGAACCCGAACTAAAAATATCCGAACCCGACCCGAAGTACAGAAATACCCGAACGGGTTCTACACCTCTATAACTGAAATATCCGAAAATCTGAAATACC  2409_1 TTAATTTTGTTTTTTCAAAATCTAAATATCCGAACTCGATCCGAAATAACCGAACCCGAACTAAAAATATCCGAACCCGACCCGAAGTACAGAAATACCCGAACGGGTTCTACACCTCTATAACTGAAATATCCGAAAATCTGAAATACC  2409_2 TTAATTTTGTTTTTTCAAAATCTAAATATCCGAACTCGATCCGAAATAACCGAACCCGAACTAAAAATATCCGAACCCGACCCGAAGTACAGAAATACCCGAACGGGTTCTACACCTCTATAACTGAAATATCCGAAAATCTGAAATACC  09WH45 TTAATTTTGTTTTTTCAAAATCTAAATATCCGAACTCGATCCGAAATAACCGAACCCGAACTAAAAATATCCGAACCCGACCCGAAGTACAGAAATACCCGAACGGGTTCTACACCTCTATAACTGAAATATCCGAAAATCTGAAATACC  842 TTAATTTTGTTTTTTCAAAATCTAAATATCCGAACTCGATCCGAAATAACCGAACCCGAACTAAAAATATCCGAACCCGACCCGAAGTACAGAAATACCCGAACGGGTTCTACACCTCTATAACTGAAATATCCGAAAATCTGAAATACC  Kale_A TTAATTTTGTTTTTTCAAAATCTAAATATCCGAACTCGATCCGAAATAACCGAACCCGAACTAAAAATATCCGAACCCGACCCGAAGTACAGAAATACCCGAACGGGTTCTACACCTCTATAACTGAAATATCCGAAAATCTGAAATACC  Cauliflower_A TTAATTTTGTTTTTTCAAAATCTAAATATCCGAACTCGATCCGAAATAACCGAACCCGAACTAAAAATATCCGAACCCGACCCGAAGTACAGAAATACCCGAACGGGTTCTACACCTCTATAACTGAAATATCCGAAAATCTGAAATACC  B90_1 GTA---------TTTTAGAA---AAATATCCGAACTCGATCCGAAATAACCGAACCCGAACTAAAAATATCCGAACCCGACCCGAAGTACAGAAATACCCGAACGGGTTCTACACCTCTATAACTGAAATATCCGAAAATCTGAAATACC  B90_2 GTA---------TTTTAGAA---AAATATCCGAACTCGATCCGAAATAACCGAACCCGAACTAAAAATATCCGAACCCGACCCGAAGTACAGAAATACCCGAACGGGTTCTACACCTCTATAACTGAAATATCCGAAAATCTGAAATACC  PurpleB GTA---------TTTTAGAA---AAATATCCGAACTCGATCCGAAATAACCGAACCCGAACTAAAAATATCCGAACCCGACCCGAAGTACAGAAATACCCGAACGGGTTCTACACCTCTATAACTGAAATATCCGAAAATCTGAAATACC  ** *** * ** ****** ******************************************************************************************************** ***************  **159 bp Repeat**  TO1000 CGACCCGAACCCGAACGGATAGCCGAACGCCCACCCCTAGTTCCTATTTCACAAAGTTATCTGTCTGGGTAAAGATCTTTTTTCTACACTATCAAAACCATATATAGTTTGCCAGAAAGTATGTACCTGTTAAAAATATGGATTAGAGAG  Kale_B CGACCCGAACCCGAACGGATAGCCGAACGCCCACCCCTAGTTCCTATTTCACAAAGTTATCTGTCTGGGTAAAGATCTTTTTTCTACACTATCAAAACCATATATAGTTTGCCAGAAAGTATGTACCTGTTAAAAATATGGATTAGAGAG  Cauliflower_B CGACCCGAACCCGAACGGATAGCCGAACGCCCACCCCTAGTTCCTATTTCACAAAGTTATCTGTCTGGGTAAAGATCTTTTTTCTACACTATCAAAACCATATATAGTTTGCCAGAAAGTATGTACCTGTTAAAAATATGGATTAGAGAG  154_1 CGACCCGAACCCGAACGGATAGCCGAACGCCCACCCCTAGTTCCTATTTCACAAAGTTATCTGTCTGGGTAAAGATCTTTTTTCTACACTATCAAAACCATATATAGTTTGCCAGAAAGTATGTACCTGTTAAAAATATGGATTAGAGAG  GreenB CGACCCGAACCCGAACGGATAGCCGAACGCCCACCCCTAGTTCCTATTTCACAAACTTTTTT-------------TTTTTTTTTTTGATTAAAAAAA----AAAAAGTTTGCCAGAAAGTATGTACCTGTTAAAAATATGGATTAGAGAG  154_2 CGACCCGAACCCGAACGGATAGCCGAACGCCCACCCCTAGTTCCTATTTCACAAAGTTATCTGTCTGGGTAAAGATCTTTTTTCTACACTATCAAAACCATATATAGTTTGCCAGAAAGTATGTACCTGTTAAAAATATGGATTAGAGAG  Broccoli CGACCCGAACCCGAACGGATAGCCGAACGCCCACCCCTAGTTCCTATTTCACAAAGTTATCTGTCTGGGTAAAGATCTTTTTTCTACACTATCAAAACCATATATAGTTTGCCAGAAAGTATGTACCTGTTAAAAATATGGATTAGAGAG  GreenA CGACCCGAACCCGAACGGATAGCCGAACGCCCACCCCTAGTTCCTATTTCACAAAGTTATCTGTCTGGGTAAAGATCTTTTTTCTACACTATCAAAACCATATATAGTTTGCCAGAAAGTATGTACCTGTTAAAAATATGGATTAGAGAG  2409_1 CGACCCGAACCCGAACGGATAGCCGAACGCCCACCCCTAGTTCCTATTTCACAAAGTTATCTGTCTGGGTAAAGATCTTTTTTCTACACTATCAAAACCATATATAGTTTGCCAGAAAGTATGTACCTGTTAAAAATATGGATTAGAGAG  2409_2 CGACCCGAACCCGAACGGATAGCCGAACGCCCACCCCTAGTTCCTATTTCACAAAGTTATCTGTCTGGGTAAAGATCTTTTTTCTACACTATCAAAACCATATATAGTTTGCCAGAAAGTATGTACCTGTTAAAAATATGGATTAGAGAG  09WH45 CGACCCGAACCCGAACGGATAGCCGAACGCCCACCCCTAGTTCCTATTTCACAAAGTTATCTGTCTGGGTAAAGATCTTTTTTCTACACTATCAAAACCATATATAGTTTGCCAGAAAGTATGTACCTGTTAAAAATATGGATTAGAGAG  842 CGACCCGAACCCGAACGGATAGCCGAACGCCCACCCCTAGTTCCTATTTCACAAAGTTATCTGTCTGGGTAAAGATCTTTTTTCTACACTATCAAAACCATATATAGTTTGCCAGAAAGTATGTACCTGTTAAAAATATGGATTAGAGAG  Kale_A CGACCCGAACCCGAACGGATAGCCGAACGCCCACCCCTAGTTCCTATTTCACAAAGTTATCTGTCTGGGTAAAGATCTTTTTTCTACACTATCAAAACCATATATAGTTTGCCAGAAAGTATGTACCTGTTAAAAATATGGATTAGAGAG  Cauliflower_A CGACCCGAACCCGAACGGATAGCCGAACGCCCACCCCTAGTTCCTATTTCACAAAGTTATCTGTCTGGGTAAAGATCTTTTTTCTACACTATCAAAACCATATATAGTTTGCCAGAAAGTATGTACCTGTTAAAAATATGGATTAGAGAG  B90_1 CGACCCGAACCCGAACGGATAGCCGAACGCCCACCCCTAGTTCCTATTTCACAAAGTTATCTGTCTGGGTAAAGATCTTTTTTCTACACTATCAAAACCATATATAGTTTGCCAGAAAGTATGTACCTGTTAAAAATATGGATTAGAGAG  B90_2 CGACCCGAACCCGAACGGATAGCCGAACGCCCACCCCTAGTTCCTATTTCACAAAGTTATCTGTCTGGGTAAAGATCTTTTTTCTACACTATCAAAACCATATATAGTTTGCCAGAAAGTATGTACCTGTTAAAAATATGGATTAGAGAG  PurpleB CGACCCGAACCCGAACGGATAGCCGAACGCCCACCCCTAGTTCCTATTTCACAAAGTTATCTGTCTGGGTAAAGATCTTTTTTCTACACTATCAAAACCATATATAGTTTGCCAGAAAGTATGTACCTGTTAAAAATATGGATTAGAGAG  ******************************************************* ** * * * ****** * * ** **** * * *********************************************  TO1000 AGGTTTGGATTGGAATGATGTAGGTAAGGCATATTAAATGCAAGCTCTTTCCACATTTATGATTCCACGGGTCAGTGGAAGCATCACCGTGGTGGTTAT---GTCTATTAGAATTGAATAAATGTGGTAAATGTGTTCTACTTCTTCTTC  Kale_B AGGTTTGGATTGGAATGATGTAGGTAAGGCATATTAAATGCAAGCTCTTTCCACATTTATGATTCCACGGGTCAGTGGAAGCATCACCGTGGTGGTTAT---GTCTATTAGAATTGAATAAATGTGGTAAATGTGTTCTACTTCTTCTTC  Cauliflower_B AGGTTTGGATTGGAATGATGTAGGTAAGGCATATTAAATGCAAGCTCTTTCCACATTTATGATTCCACGGGTCAGTGGAAGCATCACCGTGGTGGTTAT---GTCTATTAGAATTGAATAAATGTGGTAAATGTGTTCTACTTCTTCTTC  154_1 AGGTTTGGATTGGAATGATGTAGGTAAGGCATATTAAATGCAAGCTCTTTCCACATTTATGATTCCACGGGTCAGTGGAAGCATCACCGTGGTGGTTATTATGTCTATTAGAATTGAATAAATGTGGTAAATGTGTTCTACTTCTTCTTC  GreenB AGGTTTGGATTGGAATGATGTAGGTAAGGCATATTAAATGCAAGCTCTTTCCACATTTATGATTCCACGGGTCAGTGGAAGCATCACCGTGGTGGTTATTATGTCTATTAGAATTGAATAAATGTGGTAAATGTGTTCTACTTCTTCTTC  154_2 AGGTTTGGATTGGAATGATGTAGGTAAGGCATATTAAATGCAAGCTCTTTCCACATTTATGATTCCACGGGTCAGTGGAAGCATCACCGTGGTGGTTAT---GTCTATTAGAATTGAATAAGTGTGGTAAATGTGTTCTGCTTCTTCTTC  Broccoli AGGTTTGGATTGGAATGATGTAGGTAAGGCATATTAAATGCAAGCTCTTTCCACATTTATGATTCCACGGGTCAGTGGAAGCATCACCGTGGTGGTTAT---GTCTATTAGAATTGAATAAGTGTGGTAAATGTGTTCTGCTTCTTCTTC  GreenA AGGTTTGGATTGGAATGATGTAGGTAAGGCATATTAAATGCAAGCTCTTTCCACATTTATGATTCCACGGGTCAGTGGAAGCATCACCGTGGTGGTTAT---GTCTATTAGAATTGAATAAGTGTGGTAAATGTGTTCTGCTTCTTCTTC  2409_1 AGGTTTGGATTGGAATGATGTAGGTAAGGCATATTAAATGCAAGCTCTTTCCACATTTATGATTCCACGGGTCAGTGGAAGCATCACCGTGGTGGTTAT---GTCTATTAGAATTGAATAAGTGTGGTAAATGTGTTCTGCTTCTTCTTC  2409_2 AGGTTTGGATTGGAATGATGTAGGTAAGGCATATTAAATGCAAGCTCTTTCCACATTTATGATTCCACGGGTCAGTGGAAGCATCACCGTGGTGGTTAT---GTCTATTAGAATTGAATAAGTGTGGTAAATGTGTTCTGCTTCTTCTTC  09WH45 AGGTTTGGATTGGAATGATGTAGGTAAGGCATATTAAATGCAAGCTCTTTCCACATTTATGATTCCACGGGTCAGTGGAAGCATCACCGTGGTGGTTAT---GTCTATTAGAATTGAATAAGTGTGGTAAATGTGTTCTGCTTCTTCTTC  842 AGGTTTGGATTGGAATGATGTAGGTAAGGCATATTAAATGCAAGCTCTTTCCACATTTATGATTCCACGGGTCAGTGGAAGCATCACCGTGGTGGTTAT---GTCTATTAGAATTGAATAAGTGTGGTAAATGTGTTCTGCTTCTTCTTC  Kale_A AGGTTTGGATTGGAATGATGTAGGTAAGGCATATTAAATGCAAGCTCTTTCCACATTTATGATTCCACGGGTCAGTGGAAGCATCACCGTGGTGGTTAT---GTCTATTAGAATTGAATAAGTGTGGTAAATGTGTTCTGCTTCTTCTTC  Cauliflower_A AGGTTTGGATTGGAATGATGTAGGTAAGGCATATTAAATGCAAGCTCTTTCCACATTTATGATTCCACGGGTCAGTGGAAGCATCACCGTGGTGGTTAT---GTCTATTAGAATTGAATAAGTGTGGTAAATGTGTTCTGCTTCTTCTTC  B90_1 AGGTTTGGATTGGAATGATGTAGGTAAGGCATATTAAATGCAAGCTCTTTCCACATTTATGATTCCACGGGTCAGTGGAAGCATCACCGTGGTGGTTAT---GTCTATTAGAATTGAATAAGTGTGGTAAATGTGTTCTGCTTCTTCTTC  B90_2 AGGTTTGGATTGGAATGATGTAGGTAAGGCATATTAAATGCAAGCTCTTTCCACATTTATGATTCCACGGGTCAGTGGAAGCATCACCGTGGTGGTTAT---GTCTATTAGAATTGAATAAGTGTGGTAAATGTGTTCTGCTTCTTCTTC  PurpleB AGGTTTGGATTGGAATGATGTAGGTAAGGCATATTAAATGCAAGCTCTTTCCACATTTATGATTCCACGGGTCAGTGGAAGCATCACCGTGGTGGTTAT---GTCTATTAGAATTGAATAAGTGTGGTAAATGTGTTCTGCTTCTTCTTC  *************************************************************************************************** ******************* ***************** **********  TO1000 CTCGTCCAACTATTATTGATTTGACCATGCACGCTTCTCTCAACCCACCGGTCCAAGTCAAACTTCTATCCATGTAGCTATCATCCCCTATATATCAATTGCTACTCGTAAACAACCTCACAATCAATCAACTAACAATAGAAACCTACC  Kale_B CTCGTCCAACTATTATTGATTTGACCATGCACGCTTCTCTCAACCCACCGGTCCAAGTCAAACTTCTATCCATGTAGCTATCATCCCCTATATATCAATTGCTACTCGTAAACAACCTCACAATCAATCAACTAACAATAGAAACCTACC  Cauliflower_B CTCGTCCAACTATTATTGATTTGACCATGCACGCTTCTCTCAACCCACCGGTCCAAGTCAAACTTCTATCCATGTAGCTATCATCCCCTATATATCAATTGCTACTCGTAAACAACCTCACAATCAATCAACTAACAATAGAAACCTACC  154_1 CTCGTCCAACTATTATTGATTTGACCATGCACGCTTCTCTCAACCCACCGGTCCAAGTCAAACTTCTATCCATGTAGCTATCATCCCCTATATATCAATTGCTTCTCGTAAACAACCTCACAATCAATCAACTAACAATAGAAACCTACC  GreenB CTCGTCCAACTATTATTGATTTGACCATGCACGCTTCTCTCAACCCACCGGTCCAAGTCAAACTTCTATCCATGTAGCTATCATCCCCTATATATCAATTGCTTCTCGTAAACAACCTCACAATCAATCAACTAACAATAGAAACCTACC  154_2 CTCGTCCAACTATTATTGATTTGACCATGCACGCTTCTCTCAACCCACCGGTCCAAGTCAAACTTCTATCCATGTAGCTATCATCCCCTATATATCAATTGCTTCTCGTAAACAACCTCACAATCAATCAACTAACAATAGAAACCTACC  Broccoli CTCGTCCAACTATTATTGATTTGACCATGCACGCTTCTCTCAACCCACCGGTCCAAGTCAAACTTCTATCCATGTAGCTATCATCCCCTATATATCAATTGCTTCTCGTAAACAACCTCACAATCAATCAACTAACAATAGAAACCTACC  GreenA CTCGTCCAACTATTATTGATTTGACCATGCACGCTTCTCTCAACCCACCGGTCCAAGTCAAACTTCTATCCATGTAGCTATCATCCCCTATATATCAATTGCTTCTCGTAAACAACCTCACAATCAATCAACTAACAATAGAAACCTACC  2409_1 CTCGTCCAACTATTATTGATTTGACCATGCACGCTTCTCTCAACCCACCGGTCCAAGTCAAACTTCTATCCATGTAGCTATCATCCCCTATATATCAATTGCTTCTCGTAAACAACCTCACAATCAATCAACTAACAATAGAAACCTACC  2409_2 CTCGTCCAACTATTATTGATTTGACCATGCACGCTTCTCTCAACCCACCGGTCCAAGTCAAACTTCTATCCATGTAGCTATCATCCCCTATATATCAATTGCTTCTCGTAAACAACCTCACAATCAATCAACTAACAATAGAAACCTACC  09WH45 CTCGTCCAACTATTATTGATTTGACCATGCACGCTTCTCTCAACCCACCGGTCCAAGTCAAACTTCTATCCATGTAGCTATCATCCCCTATATATCAATTGCTTCTCGTAAACAACCTCACAATCAATCAACTAACAATAGAAACCTACC  842 CTCGTCCAACTATTATTGATTTGACCATGCACGCTTCTCTCAACCCACCGGTCCAAGTCAAACTTCTATCCATGTAGCTATCATCCCCTATATATCAATTGCTTCTCGTAAACAACCTCACAATCAATCAACTAACAATAGAAACCTACC  Kale_A CTCGTCCAACTATTATTGATTTGACCATGCACGCTTCTCTCAACCCACCGGTCCAAGTCAAACTTCTATCCATGTAGCTATCATCCCCTATATATCAATTGCTTCTCGTAAACAACCTCACAATCAATCAACTAACAATAGAAACCTACC  Cauliflower_A CTCGTCCAACTATTATTGATTTGACCATGCACGCTTCTCTCAACCCACCGGTCCAAGTCAAACTTCTATCCATGTAGCTATCATCCCCTATATATCAATTGCTTCTCGTAAACAACCTCACAATCAATCAACTAACAATAGAAACCTACC  B90_1 CTCGTCCAACTATTATTGATTTGACCATGCACGCTTCTCTCAACCCACCGGTCCAAGTCAAACTTCTATCCATGTAGCTATCATCCCCTATATATCAATTGCTTCTCGTAAACAACCTCACAATCAATCAACTAACAATAGAAACCTACC  B90_2 CTCGTCCAACTATTATTGATTTGACCATGCACGCTTCTCTCAACCCACCGGTCCAAGTCAAACTTCTATCCATGTAGCTATCATCCCCTATATATCAATTGCTTCTCGTAAACAACCTCACAATCAATCAACTAACAATAGAAACCTACC  PurpleB CTCGTCCAACTATTATTGATTTGACCATGCACGCTTCTCTCAACCCACCGGTCCAAGTCAAACTTCTATCCATGTAGCTATCATCCCCTATATATCAATTGCTTCTCGTAAACAACCTCACAATCAATCAACTAACAATAGAAACCTACC  ******************************************************************************************************* **********************************************  TO1000 AGTCTCTCCTTTGAAGAAGACATGAACAAAATTAGCCACGGCGCTCTATCTCGGCCTTCCGGTAACGTTTCTCGTTCAATATTGTTGTATTTGCTTTCATATGACCAAATTCTTCATAATTAAAGATCGGTATAGAAGTCATAGATTACA  Kale_B AGTCTCTCCTTTGAAGAAGACATGAACAAAATTAGCCACGGCGCTCTATCTCGGCCTTCCGGTAACGTTTCTCGTTCAATATTGTTGTATTTGCTTTCATATGACCAAATTCTTCATAATTAAAGATCGGTATAGAAGTCATAGATTACA  Cauliflower_B AGTCTCTCCTTTGAAGAAGACATGAACAAAATTAGCCACGGCGCTCTATCTCGGCCTTCCGGTAACGTTTCTCGTTCAATATTGTTGTATTTGCTTTCATATGACCAAATTCTTCATAATTAAAGATCGGTATAGAAGTCATAGATTACA  154_1 AGTCTCTCCTTTGAAGAAGACATGAACAAAATTAGCCACGGCGCTCTATCTCGGCCTTCCGGTAACGTTTCTTGTTCAATATTGTTGTATTTGCTTTCATATGACCAAATTCTTCATAATTAAAGATCGGTATAGAAGTCATAGATTACA  GreenB AGTCTCTCCTTTGAAGAAGACATGAACAAAATTAGCCACGGCGCTCTATCTCGGCCTTCCGGTAACGTTTCTTGTTCAATATTGTTGTATTAGCTTTCATATGACCAAATTCTTCATAATTAAAGATCGGTATAGAAGTCATAGATTACA  154_2 AGTCTCTCCTTTGAAGAAGACATGAACAAAATTAGCCACGGCGCTCTATCTCGGCCTTCCGGTAACGTTTCTTGTTCAATATTGTTGTATTAGCTTTCATATGACCAAATTCTTCATAATTAAAGATCGGTATAGAAGTCATAGATTACA  Broccoli AGTCTCTCCTTTGAAGAAGACATGAACAAAATTAGCCACGGCGCTCTATCTCGGCCTTCCGGTAACGTTTCTTGTTCAATATTGTTGTATTTGCTTTCATATGACCAAATTCTTCATAATTAAAGATCGGTATAGAAGTCATAGATTACA  GreenA AGTCTCTCCTTTGAAGAAGACATGAACAAAATTAGCCACGGCGCTCTATCTCGGCCTTCCGGTAACGTTTCTTGTTCAATATTGTTGTATTTGCTTTCATATGACCAAATTCTTCATAATTAAAGATCGGTATAGAAGTCATAGATTACA  2409_1 AGTCTCTCCTTTGAAGAAGACATGAACAAAATTAGCCACGGCGCTCTATCTCGGCCTTCCGGTAACGTTTCTTGTTCAATATTGTTGTATTTGCTTTCATATGACCAAATTCTTCATAATTAAAGATCGGTATAGAAGTCATAGATTACA  2409_2 AGTCTCTCCTTTGAAGAAGACATGAACAAAATTAGCCACGGCGCTCTATCTCGGCCTTCCGGTAACGTTTCTTGTTCAATATTGTTGTATTTGCTTTCATATGACCAAATTCTTCATAATTAAAGATCGGTATAGAAGTCATAGATTACA  09WH45 AGTCTCTCCTTTGAAGAAGACATGAACAAAATTAGCCACGGCGCTCTATCTCGGCCTTCCGGTAACGTTTCTTGTTCAATATTGTTGTATTTGCTTTCATATGACCAAATTCTTCATAATTAAAGATCGGTATAGAAGTCATAGATTACA  842 AGTCTCTCCTTTGAAGAAGACATGAACAAAATTAGCCACGGCGCTCTATCTCGGCCTTCCGGTAACGTTTCTTGTTCAATATTGTTGTATTTGCTTTCATATGACCAAATTCTTCATAATTAAAGATCGGTATAGAAGTCATAGATTACA  Kale_A AGTCTCTCCTTTGAAGAAGACATGAACAAAATTAGCCACGGCGCTCTATCTCGGCCTTCCGGTAACGTTTCTTGTTCAATATTGTTGTATTTGCTTTCATATGACCAAATTCTTCATAATTAAAGATCGGTATAGAAGTCATAGATTACA  Cauliflower_A AGTCTCTCCTTTGAAGAAGACATGAACAAAATTAGCCACGGCGCTCTATCTCGGCCTTCCGGTAACGTTTCTTGTTCAGTATTGTTGTATTTGCTTTCATATGACCAAATTCTTCATAATTAAAGATCGGTATAGAAGTCATAGATTACA  B90_1 AGTCTCTCCTTTGAAGAAGACATGAACAAAATTAGCCACGGCGCTCTATCTCGGCCTTCCGGTAACGTTTCTTGTTCAATATTGTTGTATTTGCTTTCATATGACCAAATTCTTCATAATTAAAGATCGGTATAGAAGTCATAGATTACA  B90_2 AGTCTCTCCTTTGAAGAAGACATGAACAAAATTAGCCACGGCGCTCTATCTCGGCCTTCCGGTAACGTTTCTTGTTCAATATTGTTGTATTTGCTTTCATATGACCAAATTCTTCATAATTAAAGATCGGTATAGAAGTCATAGATTACA  PurpleB AGTCTCTCCTTTGAAGAAGACATGAACAAAATTAGCCACGGCGCTCTATCTCGGCCTTCCGGTAACGTTTCTTGTTCAATATTGTTGTATTTGCTTTCATATGACCAAATTCTTCATAATTAAAGATCGGTATAGAAGTCATAGATTACA  ************************************************************************ ***** ************ **********************************************************  TO1000 TATATGTACATTTGCACGGGTGAGTTTGCAACAAATGTCGTTTTACTTTGTGAAATTTAATCCCTAATCATGTTTTAGGAATGCTGCACCGTGCCAAGAGGTATAGAGGGAGAAAGTACGCAAAGCCAGAACTTAAACAAAGCAACTTCT  Kale_B TATATGTACATTTGCACGGGTGAGTTTGCAACAAATGTCGTTTTACTTTGTGAAATTTAATCCCTAATCATGTTTTAGGAATGCTGCACCGTGCCAAGAGGTATAGAGGGAGAAAGTACGCAAAGCCAGAACTTAAACAAAGCAACTTCT  Cauliflower_B TATATGTACATTTGCACGGGTGAGTTTGCAACAAATGTCGTTTTACTTTGTGAAATTTAATCCCTAATCATGTTTTAGGAATGCTGCACCGTGCCAAGAGGTATAGAGGGAGAAAGTACGCAAAGCCAGAACTTAAACAAAGCAACTTCT  154_1 TATATGTACATTTGCACGGGTGAGTTTGCAACAAATGTCGTTTTACTTTGTGAAATTTAATCCCTAATCATGTTTTAGGAATGCTGCACCGTGCCAAGAGGTATAGAGGGAGAAAGTACGCAAAGCCAGAACTTAAACAAAGCAACTTCT  GreenB TATATGTACATTTGCACGGGTGAGTTTGCAACAAATGTCGTTTTACTTTGTGAAATTTAATCCCTAATCATGTTTTAGGAATGCTGCACCGTGCCAAGAGGTATAGAGGGAGAAAGTACGCAAAGCCAGAACTTAAACAAAGCAACTTCT  154_2 TATATGTACATTTGCACGGGTGAGTTTGCAACAAATGTCGTTTTACTTTGTGAAATTTAATCCCTAATCATGTTTTAGGAATGCTGCACCGTGCCAAGAGGTATAGAGGGAGAAAGTACGCAAAGCCAGAACTTAAACAAAGCAACTTCT  Broccoli TATATGTACATTTGCACGGGTGAGTTTGCAACAAATGTCGTTTTACTTTGTGAAATTTAATCCCTAATCATGTTTTAGGAATGCTGCACCGTGCCAAGAGGTATAGAGGGAGAAAGTACGCAAAGCCAGAACTTAAACAAAGCAACTTCT  GreenA TATATGTACATTTGCACGGGTGAGTTTGCAACAAATGTCGTTTTACTTTGTGAAATTTAATCCCTAATCATGTTTTAGGAATGCTGCACCGTGCCAAGAGGTATAGAGGGAGAAAGTACGCAAAGCCAGAACTTAAACAAAGCAACTTCT  2409_1 TATATGTACATTTGCACGGGTGAGTTTGCAACAAATGTCGTTTTACTTTGTGAAATTTAATCCCTAATCATGTTTTAGGAATGCTGCACCGTGCCAAGAGGTATAGAGGGAGAAAGTACGCAAAGCCAGAACTTAAACAAAGCAACTTCT  2409_2 TATATGTACATTTGCACGGGTGAGTTTGCAACAAATGTCGTTTTACTTTGTGAAATTTAATCCCTAATCATGTTTTAGGAATGCTGCACCGTGCCAAGAGGTATAGAGGGAGAAAGTACGCAAAGCCAGAACTTAAACAAAGCAACTTCT  09WH45 TATATGTACATTTGCACGGGTGAGTTTGCAACAAATGTCGTTTTACTTTGTGAAATTTAATCCCTAATCATGTTTTAGGAATGCTGCACCGTGCCAAGAGGTATAGAGGGAGAAAGTACGCAAAGCCAGAACTTAAACAAAGCAACTTCT  842 TATATGTACATTTGCACGGGTGAGTTTGCAACAAATGTCGTTTTACTTTGTGAAATTTAATCCCTAATCATGTTTTAGGAATGCTGCACCGTGCCAAGAGGTATAGAGGGAGAAAGTACGCAAAGCCAGAACTTAAACAAAGCAACTTCT  Kale_A TATATGTACATTTGCACGGGTGAGTTTGCAACAAATGTCGTTTTACTTTGTGAAATTTAATCCCTAATCATGTTTTAGGAATGCTGCACCGTGCCAAGAGGTATAGAGGGAGAAAGTACGCAAAGCCAGAACTTAAACAAAGCAACTTCT  Cauliflower_A TATATGTACATTTGCACGGGTGAGTTTGCAACAAATGTCGTTTTACTTTGTGAAATTTAATCCCTAATCATGTTTTAGGAATGCTGCACCGTGCCAAGAGGTATAGAGGGAGAAAGTACGCAAAGCCAGAACTTAAACAAAGCAACTTCT  B90_1 TATATGTACATTTGCACGGGTGAGTTTGCAACAAATGTCGTTTTACTTTGTGAAATTTAATCCCTAATCATGTTTTAGGAATGCTGCACCGTGCCAAGAGGTATAGAGGGAGAAAGTACGCAAAGCCAGAACTTAAACAAAGCAACTTCT  B90_2 TATATGTACATTTGCACGGGTGAGTTTGCAACAAATGTCGTTTTACTTTGTGAAATTTAATCCCTAATCATGTTTTAGGAATGCTGCACCGTGCCAAGAGGTATAGAGGGAGAAAGTACGCAAAGCCAGAACTTAAACAAAGCAACTTCT  PurpleB TATATGTACATTTGCACGGGTGAGTTTGCAACAAATGTCGTTTTACTTTGTGAAATTTAATCCCTAATCATGTTTTAGGAATGCTGCACCGTGCCAAGAGGTATAGAGGGAGAAAGTACGCAAAGCCAGAACTTAAACAAAGCAACTTCT  ******************************************************************************************************************************************************  TO1000 CAAAAGACGAGGACGATCTCATCCTCAAGCTTCATGCACTTCTTGGCAATAGGTTTTCATTCTTCTTCTTTAATTCAATTTCGAGCGTTATTATAAAAATATACCGTAATGTGAATGTTTGTTTTTTTTTTT-GCAAAAAATAATGTGAA  Kale_B CAAAAGACGAGGACGATCTCATCCTCAAGCTTCATGCACTTCTTGGCAATAGGTTTTCATTCTTCTTCTTTAATTCAATTTCGAGCGTTATTATAAAAATATACCGTAATGTGAATGTTTGTTTTTTTTTTT-GCAAAAAATAATGTGAA  Cauliflower_B CAAAAGACGAGGACGATCTCATCCTCAAGCTTCATGCACTTCTTGGCAATAGGTTTTCATTCTTCTTCTTTAATTCAATTTCGAGCGTTATTATAAAAATATACCGTAATGTGAATGTTTGTTTTTTTTTT--GCAAAAAATAATGTGAA  154_1 CAAAAGACGAGGACGATCTCATCCTCAAGCTTCATGCACTTCTTGGCAATAGGTTTTCATTCTTCTTCTTTAATTCAATTTCGAGCGTTATTATAAAAATATACCGTAATGTGAATGTTTGTTTTTTTTTTT-GCAAAAAATAATGTGAA  GreenB CAAAAGACGAGGACGATCTCATCCTCAAGCTTCATGCACTTCTTGGCAATAGGTTTTCATTCTTCTTCTTTAATTCAATTTCGAGCGTTATTATAAAAATATACCGTAATGTGAATGTTTGTTTTTTTTTT--GCAAAAAATAATGTGAA  154_2 CAAAAGACGAGGACGATCTCATCCTCAAGCTTCATGCACTTCTTGGCAATAGGTTTTCATTCTTCTTCTTTAATTCAATTTCGAGCGTTATTATAAAAATATACCGTAATGTGAATGTTTGTTTTTTTTTTT-GCAAAAAATAATGTGAA  Broccoli CAAAAGACGAGGACGATCTCATCCTCAAGCTTCATGCACTTCTTGGCAATAGGTTTTCATTCTTCTTCTTTAATTCAATTTCGAGCGTTATTATAAAAATATACCGTAATGTGAATGTTTGTTTTTTTTTT--GCAAAAAATAATGTGAA  GreenA CAAAAGACGAGGACGATCTCATCCTCAAGCTTCATGCACTTCTTGGCAATAGGTTTTCATTCTTCTTCTTTAATTCAATTTCGAGCGTTATTATAAAAATATACCGTAATGTGAATGTTTGTTTTTTTTTT--GCAAAAAATAATGTGAA  2409_1 CAAAAGACGAGGACGATCTCATCCTCAAGCTTCATGCACTTCTTGGCAATAGGTTTTCATTCTTCTTCTTTAATTCAATTTCGAGCGTTATTATAAAAATATACCGTAATGTGAATGTTTGTTTTTTTTTTT-GCAAAAAATAATGTGAA  2409_2 CAAAAGACGAGGACGATCTCATCCTCAAGCTTCATGCACTTCTTGGCAATAGGTTTTCATTCTTCTTCTTTAATTCAATTTCGAGCGTTATTATAAAAATATACCGTAATGTGAATGTTTGTTTTTTTTTTT-GCAAAAAATAATGTGAA  09WH45 CAAAAGACGAGGACGATCTCATCCTCAAGCTTCATGCACTTCTTGGCAATAGGTTTTCATTCTTCTTCTTTAATTCAATTTCGAGCGTTATTATAAAAATATACCGTAATGTGAATGTTTGTTTTTTTTTTT-GCAAAAAATAATGTGAA  842 CAAAAGACGAGGACGATCTCATCCTCAAGCTTCATGCACTTCTTGGCAATAGGTTTTCATTCTTCTTCTTTAATTCAATTTCGAGCGTTATTATAAAAATATACCGTAATGTGAATGTTTGTTTTTTTTTTT-GCAAAAAATAATGTGAA  Kale_A CAAAAGACGAGGACGATCTCATCCTCAAGCTTCATGCACTTCTTGGCAATAGGTTTTCATTCTTCTTCTTTAATTCAATTTCGAGCGTTATTATAAAAATATACCGTAATGTGAATGTTTGTTTTTTTTTTT-GCAAAAAATAATGTGAA  Cauliflower_A CAAAAGACGAGGACGATCTCATCCTCAAGCTTCATGCACTTCTTGGCAATAGGTTTTCATTCTTCTTCTTTAATTCAATTTCGAGCGTTATTATAAAAATATACCGTAATGTGAATGT---TTTTTTTTTTT-GCAAAAAATAATGTGAA  B90_1 CAAAAGACGAGGACGATCTCATCCTCAAGCTTCATGCACTTCTTGGCAATAGGTTTTCATTCTTCTTCTTTAATTCAATTTCGAGCGTTATTATAAAAATATACCGTAATGTGAATGTTTGTTTTTTTTTTTTGCAAAAAATAATGTGAA  B90_2 CAAAAGACGAGGACGATCTCATCCTCAAGCTTCATGCACTTCTTGGCAATAGGTTTTCATTCTTCTTCTTTAATTCAATTTCGAGCGTTATTATAAAAATATACCGTAATGTGAATGTTTGTTTTTTTTTTTTGCAAAAAATAATGTGAA  PurpleB CAAAAGACGAGGACGATCTCATCCTCAAGCTTCATGCACTTCTTGGCAATAGGTTTTCATTCTTCTTCTTTAATTCAATTTCGAGCGTTATTATAAAAATATACCGTAATGTGAATGTTTGTTTTTTTTTTT-GCAAAAAATAATGTGAA  ********************************************************************************************************************** ********** *****************  TO1000 TGTTTGTAATAATAAGATTTTGATGGTACGCTTTCAGATGGTCATTGATCGCGGGAAGATTGCCTGGACGAACCGACAACGAAGTAAGGATCCATTGGGAAACTTGCGTAGAGAAGAAACTCATGAAAATGGGAATCGATCCAACCAATC  Kale_B TGTTTGTAATAATAAGATTTTGATGGTACGCTTTCAGATGGTCATTGATCGCGGGAAGATTGCCTGGACGAACCGACAACGAAGTAAGGATCCATTGGGAAACTTGCGTAGAGAAGAAACTCATGAAAATGGGAATCGATCCAACCAATC  Cauliflower_B TGTTTGTAATAATAAGATTTTGATGGTACGCTTTCAGATGGTCATTGATCGCGGGAAGATTGCCTGGACGAACCGACAACGAAGTAAGGATCCATTGGGAAACTTGCGTAGAGAAGAAACTCATGAAAATGGGAATCGATCCAACCAATC  154_1 TGTTTGTAATAATAAGATTTTGATGGTACGCTTTCAGATGGTCATTGATCGCGGGAAGATTGCCTGGACGAACCGACAACGAAGTAAGGATCCATTGGGAAACTTACTTAGAGAAGAAACTCATGAAAATGGGAATCGATCCAACCAATC  GreenB TGTTTGTAATAATAAGATTTTGATGGTACGCTTTCAGATGGTCATTGATCGCGGGAAGATTGCCTGGACGAACCGACAACGAAGTAAGGATCCATTGGGAAACTTACTTAGAGAAGAAACTCATGAAAATGGGAATCGATCCAACCAATC  154_2 TGTTTGTAATAATAAGATTTTGATGGTACGCTTTCAGATGGTCATTGATCGCGGGAAGATTGCCTGGACGAACCGACAACGAAGTAAGGATCCATTGGGAAACTTACTTAGAGAAGAAACTCATGAAAATGGGAATCGATCCAACCAATC  Broccoli TGTTTGTAATAATAAGATTTTGATGGTACGCTTTCAGATGGTCATTGATCGCGGGAAGATTGCCTGGACGAACCGACAACGAAGTAAGGATCCATTGGGAAACTTACTTAGAGAAGAAACTCATGAAAATGGGAATCGATCCAACCAATC  GreenA TGTTTGTAATAATAAGATTTTGATGGTACGCTTTCAGATGGTCATTGATCGCGGGAAGATTGCCTGGACGAACCGACAACGAAGTAAGGATCCATTGGGAAACTTACTTAGAGAAGAAACTCATGAAAATGGGAATCGATCCAACCAATC  2409_1 TGTTTGTAATAATAAGATTTTGATGGTACGCTTTCAGATGGTCATTGATCGCGGGAAGATTGCCTGGACGAACCGACAACGAAGTAAGGATCCATTGGGAAACTTACTTAGAGAAGAAACTCATGAAAATGGGAATCGATCCAACCAATC  2409_2 TGTTTGTAATAATAAGATTTTGATGGTACGCTTTCAGATGGTCATTGATCGCGGGAAGATTGCCTGGACGAACCGACAACGAAGTAAGGATCCATTGGGAAACTTACTTAGAGAAGAAACTCATGAAAATGGGAATCGATCCAACCAATC  09WH45 TGTTTGTAATAATAAGATTTTGATGGTACGCTTTCAGATGGTCATTGATCGCGGGAAGATTGCCTGGACGAACCGACAACGAAGTAAGGATCCATTGGGAAACTTACTTAGAGAAGAAACTCATGAAAATGGGAATCGATCCAACCAATC  842 TGTTTGTAATAATAAGATTTTGATGGTACGCTTTCAGATGGTCATTGATCGCGGGAAGATTGCCTGGACGAACCGACAACGAAGTAAGGATCCATTGGGAAACTTACTTAGAGAAGAAACTCATGAAAATGGGAATCGATCCAACCAATC  Kale_A TGTTTGTAATAATAAGATTTTGATGGTACGCTTTCAGATGGTCATTGATCGCGGGAAGATTGCCTGGACGAACCGACAACGAAGTAAGGATCCATTGGGAAACTTACTTAGAGAAGAAACTCATGAAAATGGGAATCGATCCAACCAATC  Cauliflower_A TGTTTGTAATAATAAGATTTTGATGGTACGCTTTCAGATGGTCATTGATCGCGGGAAGATTGCCTGGACGAACCGACAACGAAGTAAGGATCCATTGGGAAACTTACTTAGAGAAGAAACTCATGAAAATGGGAATCGATCCAACCAATC  B90_1 TGTTTGTAATAATAAGATTTTGATGGTACGCTTTCAGATGGTCATTGATCGCGGGAAGATTGCCTGGACGAACCGACAACGAAGTAAGGATCCATTGGGAAACTTACTTAGAGAAGAAACTCATGAAAATGGGAATCGATCCAACCAATC  B90_2 TGTTTGTAATAATAAGATTTTGATGGTACGCTTTCAGATGGTCATTGATCGCGGGAAGATTGCCTGGACGAACCGACAACGAAGTAAGGATCCATTGGGAAACTTACTTAGAGAAGAAACTCATGAAAATGGGAATCGATCCAACCAATC  PurpleB TGTTTGTAATAATAAGATTTTGATGGTACGCTTTCAGATGGTCATTGATCGCGGGAAGATTGCCTGGACGAACCGACAACGAAGTAAGGATCCATTGGGAAACTTACTTAGAGAAGAAACTCATGAAAATGGGAATCGATCCAACCAATC  ********************************************************************************************************* * ******************************************  TO1000 ATCGTCTCTACCATCACACAAACTACACTTCTAGAAGATACATCAATGCCTCGTATAAGAAACATGAAACCGATATTATTAGTGATCAATCTTCTTCGGTATCGGAATCATGTAATATGACACTATTACCCGTTTCAAGTACCAAATTCT  Kale_B ATCGTCTCTACCATCACACAAACTACACTTCTAGAAGATACATCAATGCCTCGTATAAGAAACATGAAACCGATATTATTAGTGATCAATCTTCTTCGGTATCGGAATCATGTAATATGACACTATTACCCGTTTCAAGTACCAAATTCT  Cauliflower_B ATCGTCTCTACCATCACACAAACTACACTTCTAGAAGATACATCAATGCCTCGTATAAGAAACATGAAACCGATATTATTAGTGATCAATCTTCTTCGGTATCGGAATCATGTAATATGACACTATTACCCGTTTCAAGTACCAAATTCT  154_1 ATCGTCTCTACCATCACACAAACTACACTTCTAGAAGATACATCAATGCCTCGTATAAGAAACATGAAACCGATATTATTAGTGATCAATCTTCTTCGGTATCGGAATCATGTAATATGACACTATTACCCGTTTCAAGTACCAAATTCT  GreenB ATCGTCTCTACCATCACACAAACTACACTTCTAGAAGATACATCAATGCCTCGTATAAGAAACATGAAACCGATATTATTAGTGATCAATCTTCTTCGGTATCGGAATCATGTAATATGACACTATTACCCGTTTCAAGTACCAAATTCT  154_2 ATCGTCTCTACCATCACACAAACTACACTTCTAGAAGATACATCAATGCCTCGTATAAGAAACATGAAACCGATATTATTAGTGATCAATCTTCTTCGGTATCGGAATCATGTAATATGACACTATTACCCGTTTCAAGTACCAAATTCT  Broccoli ATCGTCTCTACCATCACACAAACTACACTTCTAGAAGATACATCAATGCCTCGTATAAGAAACATGAAACCGATATTATTAGTGATCAATCTTCTTCGGTATCGGAATCATGTAATATGACACTATTACCCGTTTCAAGTACCAAATTCT  GreenA ATCGTCTCTACCATCACACAAACTACACTTCTAGAAGATACATCAATGCCTCGTATAAGAAACATGAAACCGATATTATTAGTGATCAATCTTCTTCGGTATCGGAATCATGTAATATGACACTATTACCCGTTTCAAGTACCAAATTCT  2409_1 ATCGTCTCTACCATCACACAAACTACACTTCTAGAAGATACATCAATGCCTCGTATAAGAAACATGAAACCGATATTATTAGTGATCAATCTTCTTCGGTATCGGAATCATGTAATATGACACTATTACCCGTTTCAAGTACCAAATTCT  2409_2 ATCGTCTCTACCATCACACAAACTACACTTCTAGAAGATACATCAATGCCTCGTATAAGAAACATGAAACCGATATTATTAGTGATCAATCTTCTTCGGTATCGGAATCATGTAATATGACACTATTACCCGTTTCAAGTACCAAATTCT  09WH45 ATCGTCTCTACCATCACACAAACTACACTTCTAGAAGATACATCAATGCCTCGTATAAGAAACATGAAACCGATATTATTAGTGATCAATCTTCTTCGGTATCGGAATCATGTAATATGACACTATTACCCGTTTCAAGTACCAAATTCT  842 ATCGTCTCTACCATCACACAAACTACACTTCTAGAAGATACATCAATGCCTCGTATAAGAAACATGAAACCGATATTATTAGTGATCAATCTTCTTCGGTATCGGAATCATGTAATATGACACTATTACCCGTTTCAAGTACCAAATTCT  Kale_A ATCGTCTCTACCATCACACAAACTACACTTCTAGAAGATACATCAATGCCTCGTATAAGAAACATGAAACCGATATTATTAGTGATCAATCTTCTTCGGTATCGGAATCATGTAATATGACACTATTACCCGTTTCAAGTACCAAATTCT  Cauliflower_A ATCGTCTCTACCATCACACAAACTACACTTCTAGAAGATACATCAATGCCTCGTATAAGAAACATGAAACCGATATTATTAGTGATCAATCTTCTTCGGTATCGGAATCATGTAATATGACACTATTACCCGTTTCAAGTACCAAATTCT  B90_1 ATCGTCTCTACCATCACACAAACTACACTTCTAGAAGATACATCAATGCCTCGTATAAGAAACATGAAACCGATATTATTAGTGATCAATCTTCTTCGGTATCGGAATCATGTAATATGACACTATTACCCGTTTCAAGTACCAAATTCT  B90_2 ATCGTCTCTACCATCACACAAACTACACTTCTAGAAGATACATCAATGCCTCGTATAAGAAACATGAAACCGATATTATTAGTGATCAATCTTCTTCGGTATCGGAATCATGTAATATGACACTATTACCCGTTTCAAGTACCAAATTCT  PurpleB ATCGTCTCTACCATCACACAAACTACACTTCTAGAAGATACATCAATGCCTCGTATAAGAAACATGAAACCGATATTATTAGTGATCAATCTTCTTCGGTATCGGAATCATGTAATATGACACTATTACCCGTTTCAAGTACCAAATTCT  ******************************************************************************************************************************************************  TO1000 CTGAGGATAATGCTAGTGCCGGACATAACTGGTTGCCTGACCTCAACATCGGTCTCGTCCCGATGAAAACCGTGACTTCTTTGCCAGTTTGCTCCCTTCAAGAACCTAGCGAATCCTCTAACCATGGTTCAACGAGTCAAGAAACACTTC  Kale_B CTGAGGATAATGCTAGTGCCGGACATAACTGGTTGCCTGACCTCAACATCGGTCTCGTCCCGATGAAAACCGTGACTTCTTTGCCAGTTTGCTCCCTTCAAGAACCTAGCGAATCCTCTAACCATGGTTCAACGAGTCAAGAAACACTTC  Cauliflower_B CTGAGGATAATGCTAGTGCCGGACATAACTGGTTGCCTGACCTCAACATCGGTCTCGTCCCGATGAAAACCGTGACTTCTTTGCCAGTTTGCTCCCTTCAAGAACCTAGCGAATCCTCTAACCATGGTTCAACGAGTCAAGAAACACTTC  154_1 CTAAGGATAATGCTACTGCCGGACATAACTGGTTGCCTGACCTCAACATCGGTCTCGTCCCGATGAAAACCGTGACTTCTTTGCCAGTTTGCTCCCTTCAAGAACCTAGCGAATCCTCTAACCATGGTTCAACGAGTCAAGAAACACTTC  GreenB CTGAGGATAATGCTAGTGCCGGACATAACTGGTTGCCTGACCTCAACATCGGTCTCGTCCCGATGAAAACCGTGACTTCTTTGCCAGTTTGCTCCCTTCAAGAACCTAGCGAATCCTCTAACCATGGTTCAACGAGTCAAGAAACACTTC  154_2 CTAAGGATAATGCTACTGCCGGACATAACTGGTTGCCTGACCTCAACATCGGTCTCGTCCCGATGAAAACCGTGACTTCTTTGCCAGTTTGCTCCCTTCAAGAACCTAGCGAATCCTCTAACCATGGTTCAACGAGTCAAGAAACACTTC  Broccoli CTAAGGATAATGCTACTGCCGGACATAACTGGTTGCCTGACCTCAACATCGGTCTCGTCCCGATGAAAACCGTGACTTCTTTGCCAGTTTGCTCCCTTCAAGAACCTAGCGAATCCTCTAACCATGGTTCAACGAGTCAAGAAACACTTC  GreenA CTAAGGATAATGCTACTGCCGGACATAACTGGTTGCCTGACCTCAACATCGGTCTCGTCCCGATGAAAACCGTGACTTCTTTGCCAGTTTGCTCCCTTCAAGAACCTAGCGAATCCTCTAACCATGGTTCAACGAGTCAAGAAACACTTC  2409_1 CTAAGGATAATGCTAGTGCCGGACATAACTGGTTGCCTGACCTCAACATCGGTCTCGTCCCGATGAAAACCGTGACTTCTTTGCCAGTTTGCTCCCTTCAAGAACCTAGCGAATCCTCTAACCATGGTTCAACGAGTCAAGAAACACTTC  2409_2 CTAAGGATAATGCTAGTGCCGGACATAACTGGTTGCCTGACCTCAACATCGGTCTCGTCCCGATGAAAACCGTGACTTCTTTGCCAGTTTGCTCCCTTCAAGAACCTAGCGAATCCTCTAACCATGGTTCAACGAGTCAAGAAACACTTC  09WH45 CTAAGGATAATGCTAGTGCCGGACATAACTGGTTGCCTGACCTCAACATCGGTCTCGTCCCGATGAAAACCGTGACTTCTTTGCCAGTTTGCTCCCTTCAAGAACCTAGCGAATCCTCTAACCATGGTTCAACGAGTCAAGAAACACTTC  842 CTAAGGATAATGCTAGTGCCGGACATAACTGGTTGCCTGACCTCAACATCGGTCTCGTCCCGATGAAAACCGTGACTTCTTTGCCAGTTTGCTCCCTTCAAGAACCTAGCGAATCCTCTAACCATGGTTCAACGAGTCAAGAAACACTTC  Kale_A CTAAGGATAATGCTACTGCCGGACATAACTGGTTGCCTGACCTCAACATCGGTCTCGTCCCGATGAAAACCGTGACTTCTTTGCCAGTTTGCTCCCTTCAAGAACCTAGCGAATCCTCTAACCATGGTTCAACGAGTCAAGAAACACTTC  Cauliflower_A CTGAGGATAATGCTAGTGCCGGACATAACTGGTTGCCTGACCTCAACATCGGTCTCGTCCCGATGAAAACCGTGACTTCTTTGCCAGTTTGCTCCCTTCAAGAACCTAGCGAATCCTCTAACCATGGTTCAACGAGTCAAGAAACACTTC  B90_1 CTAAGGATAATGCTAGTGCCGGACATAACTGGTTGCCTGACCTCAACATCGGTCTCGTCCCGATGAAAACCGTGACTTCTTTGCCAGTTTGCTCCCTTCAAGAACCTAGCGAATCCTCTAACCATGGTTCAACGAGTCAAGAAACACTTC  B90_2 CTAAGGATAATGCTAGTGCCGGACATAACTGGTTGCCTGACCTCAACATCGGTCTCGTCCCGATGAAAACCGTGACTTCTTTGCCAGTTTGCTCCCTTCAAGAACCTAGCGAATCCTCTAACCATGGTTCAACGAGTCAAGAAACACTTC  PurpleB CTAAGGATAATGCTAGTGCCGGACATAACTGGTTGCCTGACCTCAACATCGGTCTCGTCCCGATGAAAACCGTGACTTCTTTGCCAGTTTGCTCCCTTCAAGAACCTAGCGAATCCTCTAACCATGGTTCAACGAGTCAAGAAACACTTC  ** ************ **************************************************************************************************************************************  TO1000 TTCTTTTCCGGTGAAGAAATGTACTGTGAGATCAAGGAAAACTCTTAGTCAAAGTATTTGTACACATCTTCACATATACCTACCACTTGATAATAAAATGATTTTTGAAAAATTTAATCCCGAGTTGGACCGGCTGATTTGCATCAGACT  Kale_B TTCTTTTCCGGTGAAGAAATGTACTGTGAGATCAAGGAAAACTCTTAGTCAAAGTATTTGTACACATCTTCACATATACCTACCACTTGATAATAAAATGATTTTTGAAAAATTTAATCCCGAGTTGGACCGGCTGATTTGCATCAGACT  Cauliflower_B TTCTTTTCCGGTGAAGAAATGTACTGTGAGATCAAGGAAAACTCTTAGTCAAAGTATTTGTACACATCTTCACATATACCTACCACTTGATAATAAAATGATTTTTGAAAAATTTAATCCCGAGTTGGACCGGCTGATTTGCATCAGACT  154_1 TTCTTTTCCGGTGAAGAAATGTACTGTGAGATCAAGGAAAACTCTTAGTCAAAGTATTTGTACACATCTTCACATATACCTACCACTTGATAATAAAATGATTTTTGAAAAATTTAATCCCGAGTTGGACCGGCTGATTTGCATCAGACT  GreenB TTCTTTTCCGGTGAAGAAATGTACTGTGAGATCAAGGAAAACTCTTAGTCAAAGTATTTGTACACATCTTCACATATACCTACCACTTGATAATAAAATGATTTTTGAAAAATTTAATCCCGAGTTGGACCGGCTGATTTGCATCAGACT  154_2 TTCTTTTCCGGTGAAGAAATGTACTGTGAGATCAAGGAAAACTCTTAGTCAAAGTATTTGTACACATCTTCACATATACCTACCACTTGATAATAAAATGATTTTTGAAAAATTTAATCCCGAGTTGGACCGGCTGATTTGCATCAGACT  Broccoli TTCTTTTCCGGTGAAGAAATGTACTGTGAGATCAAGGAAAACTCTTAGTCAAAGTATTTGTACACATCTTCACATATACCTACCACTTGATAATAAAATGATTTTTGAAAAATTTAATCCCGAGTTGGACCGGCTGATTTGCATCAGACT  GreenA TTCTTTTCCGGTGAAGAAATGTACTGTGAGATCAAGGAAAACTCTTAGTCAAAGTATTTGTACACATCTTCACATATACCTACCACTTGATAATAAAATGATTTTTGAAAAATTTAATCCCGAGTTGGACCGGCTGATTTGCATCAGACT  2409_1 TTCTTTTCCGGTGAAGAAATGTACTGTGAGATCAAGGAAAACTCTTAGTCAAAGTATTTGTACACATCTTCACATATACCTACCACTTGATAATAAAATGATTTTTGAAAAATTTAATCCCGAGTTGGACCGGCTGATTTGCATCAGACT  2409_2 TTCTTTTCCGGTGAAGAAATGTACTGTGAGATCAAGGAAAACTCTTAGTCAAAGTATTTGTACACATCTTCACATATACCTACCACTTGATAATAAAATGATTTTTGAAAAATTTAATCCCGAGTTGGACCGGCTGATTTGCATCAGACT  09WH45 TTCTTTTCCGGTGAAGAAATGTACTGTGAGATCAAGGAAAACTCTTAGTCAAAGTATTTGTACACATCTTCACATATACCTACCACTTGATAATAAAATGATTTTTGAAAAATTTAATCCCGAGTTGGACCGGCTGATTTGCATCAGACT  842 TTCTTTTCCGGTGAAGAAATGTACTGTGAGATCAAGGAAAACTCTTAGTCAAAGTATTTGTACACATCTTCACATATACCTACCACTTGATAATAAAATGATTTTTGAAAAATTTAATCCCGAGTTGGACCGGCTGATTTGCATCAGACT  Kale_A TTCTTTTCCGGTGAAGAAATGTACTGTGAGATCAAGGAAAACTCTTAGTCAAAGTATTTGTACACATCTTCACATATACCTACCACTTGATAATAAAATGATTTTTGAAAAATTTAATCCCGAGTTGGACCGGCTGATTTGCATCAGACT  Cauliflower_A TTCTTTTCCGGTGAAGAAATGTACTGTGAGATCAAGGAAAACTCTTAGTCAAAGTATTTGTACACATCTTCACATATACCTACCACTTGATAATAAAATGATTTTTGAAAAATTTAATCCCGAGTTGGACCGGCTGATTTGCATCAGACT  B90_1 TTCTTTTCCGGTGAAGAAATGTACTGTGAGATCAAGGAAAACTCTTAGTCAAAGTATTTGTACACATCTTCACATATACCTACCACTTGATAATAAAATGATTTTTGAAAAATTTAATCCCGAGTTGGACCGGCTGATTTGCATCAGACT  B90_2 TTCTTTTCCGGTGAAGAAATGTACTGTGAGATCAAGGAAAACTCTTAGTCAAAGTATTTGTACACATCTTCACATATACCTACCACTTGATAATAAAATGATTTTTGAAAAATTTAATCCCGAGTTGGACCGGCTGATTTGCATCAGACT  PurpleB TTCTTTTCCGGTGAAGAAATGTACTGTGAGATCAAGGAAAACTCTTAGTCAAAGTATTTGTACACATCTTCACATATACCTACCACTTGATAATAAAATGATTTTTGAAAAATTTAATCCCGAGTTGGACCGGCTGATTTGCATCAGACT  ******************************************************************************************************************************************************  TO1000 CGTTGATCAACTATTCTCTCAATCAAATGTCCGGTTTCTTTTGGTGTGGAACCATTGGTATAAGTAAACCGTTGATAACCCGTGACCGGTTCAATAAAGCCGTAAGATATTAGCTATCAGAGATAAGAAATTGCGGTGCACGTTGAATGG  Kale_B CGTTGATCAACTATTCTCTCAATCAAATGTCCGGTTTCTTTTGGTGTGGAACCATTGGTATAAGTAAACCGTTGATAACCCGTGACCGGTTCAATAAAGCCGTAAGATATTAGCTATCAGAGATAAGAAATTGCGGTGCACGTTGAATGG  Cauliflower_B CGTTGATCAACTATTCTCTCAATCAAATGTCCGGTTTCTTTTGGTGTGGAACCATTGGTATAAGTAAACCGTTGATAACCCGTGACCGGTTCAATAAAGCCGTAAGATATTAGCTATCAGAGATAAGAAATTGCGGTGCACGTTGAATGG  154_1 CGTTGATCAACTATCCTCTTAATCAAATGTCCGGTTTCTTTTGGTGTGGAACCATTGGTATAAGTAAACCGTTGATAACCCGTGACCGGTTCAATAAAGCCGTAAGATATTAGCTATCAGAGATAAGAAATTGCGGTGCACGTTGAATGG  GreenB CGTTGATCAACTATCCTCTTAATCAAATGTCCGGTTTCTTTTGGTGTGGAACCATTGGTATAAGTAAACCGTTGATAACCCGTGACCGGTTCAATAAAGCCGTAAGATATTAGCTATCAGAGATAAGAAATTGCGGTGCACGTTGAATGG  154_2 CGTTGATCAACTATCCTCTTAATCAAATGTCCGGTTTCTTTTGGTGTGGAACCATTGGTATAAGTAAACCGTTGATAACCCGTGACCGGTTCAATAAAGCCGTAAGATATTAGCTATCAGAGATAAGAAATTGCGGTGCACGTTGAATGG  Broccoli CGTTGATCAACTATTCTCTTAATCAAATGTCCGGTTTCTTTTGGTGTGGAACCATTGGTATAAGTAAACCGTTGATAACCCGTGACCGGTTCAATAAAGCCGTAAGATATTAGCTATCAGAGATAAGAAATTGCGGTGCACGTTGAATGG  GreenA CGTTGATCAACTATTCTCTTAATCAAATGTCCGGTTTCTTTTGGTGTGGAACCATTGGTATAAGTAAACCGTTGATAACCCGTGACCGGTTCAATAAAGCCGTAAGATATTAGCTATCAGAGATAAGAAATTGCGGTGCACGTTGAATGG  2409_1 CGTTGATCAACTATCCTCTTAATCAAATGTCCGGTTTCTTTTGGTGTGGAACCATTGGTATAAGTAAACCGTTGATAACCCGTGACCGGTTCAATAAAGCCGTAAGATATTAGCTATCAGAGATAAGAAATTGCGGTGCACGTTGAATGG  2409_2 CGTTGATCAACTATCCTCTTAATCAAATGTCCGGTTTCTTTTGGTGTGGAACCATTGGTATAAGTAAACCGTTGATAACCCGTGACCGGTTCAATAAAGCCGTAAGATATTAGCTATCAGAGATAAGAAATTGCGGTGCACGTTGAATGG  09WH45 CGTTGATCAACTATTCTCTTAATCAAATGTCCGGTTTCTTTTGGTGTGGAACCATTGGTATAAGTAAACCGTTGATAACCCGTGACCGGTTCAATAAAGCCGTAAGATATTAGCTATCAGAGATAAGAAATTGCGGTGCACGTTGAATGG  842 CGTTGATCAACTATTCTCTTAATCAAATGTCCGGTTTCTTTTGGTGTGGAACCATTGGTATAAGTAAACCGTTGATAACCCGTGACCGGTTCAATAAAGCCGTAAGATATTAGCTATCAGAGATAAGAAATTGCGGTGCACGTTGAATGG  Kale_A CGTTGATCAACTATTCTCTTAATCAAATGTCCGGTTTCTTTTGGTGTGGAACCATTGGTATAAGTAAACCGTTGATAACCCGTGACCGGTTCAATAAAGCCGTAAGATATTAGCTATCAGAGATAAGAAATTGCGGTGCACGTTGAATGG  Cauliflower_A CGTTGATCAACTATTCTCTTAATCAAATGTCCGGTTTCTTTTGGTGTGGAACCATTGGTATAAGTAAACCGTTGATAACCCGTGACCGGTTCAATAAAGCCGTAAGATATTAGCTATCAGAGATAAGAAATTGCGGTGCACGTTGAATGG  B90_1 CGTTGATCAACTATTCTCTTAATCAAATGTCCGGTTTCTTTTGGTGTGGAACCATTGGTATAAGTAAACCGTTGATAACCCGTGACCGGTTCAATAAAGCCGTAAGATATTAGCTATCAGAGATAAGAAATTGCGGTGCACGTTGAATGG  B90_2 CGTTGATCAACTATTCTCTTAATCAAATGTCCGGTTTCTTTTGGTGTGGAACCATTGGTATAAGTAAACCGTTGATAACCCGTGACCGGTTCAATAAAGCCGTAAGATATTAGCTATCAGAGATAAGAAATTGCGGTGCACGTTGAATGG  PurpleB CGTTGATCAACTATTCTCTTAATCAAATGTCCGGTTTCTTTTGGTGTGGAACCATTGGTATAAGTAAACCGTTGATAACCCGTGACCGGTTCAATAAAGCCGTAAGATATTAGCTATCAGAGATAAGAAATTGCGGTGCACGTTGAATGG  ************** **** **********************************************************************************************************************************  TO1000 ATGCTTTCCACGCCAGTAATGAACTAACTCAC  Kale_B ATGCTTTCCACGCCAGTAATGAACTAACTCAC  Cauliflower_B ATGCTTTCCACGCCAGTAATGAACTAACTCAC  154_1 ATGCTTTCCACGCCAGTAATGAACTAACTCAC  GreenB ATGCTTTCCACGCCAGTAATGAACTAACTCAC  154_2 ATGCTTTCCACGCCAGTAATGAACTAACTCAC  Broccoli ATGCTTTCCACGCCAGTAATGAACTAACTCAC  GreenA ATGCTTTCCACGCCAGTAATGAACTAACTCAC  2409_1 ATGCTTTCCACGCCAGTAATGAACTAACTCAC  2409_2 ATGCTTTCCACGCCAGTAATGAACTAACTCAC  09WH45 ATGCTTTCCACGCCAGTAATGAACTAACTCAC  842 ATGCTTTCCACGCCAGTAATGAACTAACTCAC  Kale_A ATGCTTTCCACGCCAGTAATGAACTAACTCAC  Cauliflower_A ATGCTTTCCACGCCAGTAATGAACTAACTCAC  B90_1 ATGCTTTCCACGCCAGTAATGAACTAACTCAC  B90_2 ATGCTTTCCACGCCAGTAATGAACTAACTCAC  PurpleB ATGCTTTCCACGCCAGTAATGAACTAACTCAC  ******************************** |
| --- |
| **Additional file 7: Figure S5.** Comparison of different *BoMYBL2-1* nucleotide sequences obtained from cabbages. Shaded regions indicate exon sequences. Sequences corresponding to *Bol016162* from *B. oleracea* var. *capitata* were omitted. |
