# Supplementary material for: Purple Brassica oleracea var. capitata F. rubra is due to the loss of BoMYBL2–1 expression
Source: BMC Plant Biol. 2018 May 8;18:82. doi: 10.1186/s12870-018-1290-9 (PMC5941660; doi:10.1186/s12870-018-1290-9)
Supplement: Supplementary file 8 — Figure S4. Expression of genes associated with anthocyanin biosynthesis in various purple cabbages. Daebakna is a green cabbage used as a reference. (DOCX 387 kb) [file 12870_2018_1290_MOESM8_ESM.docx]

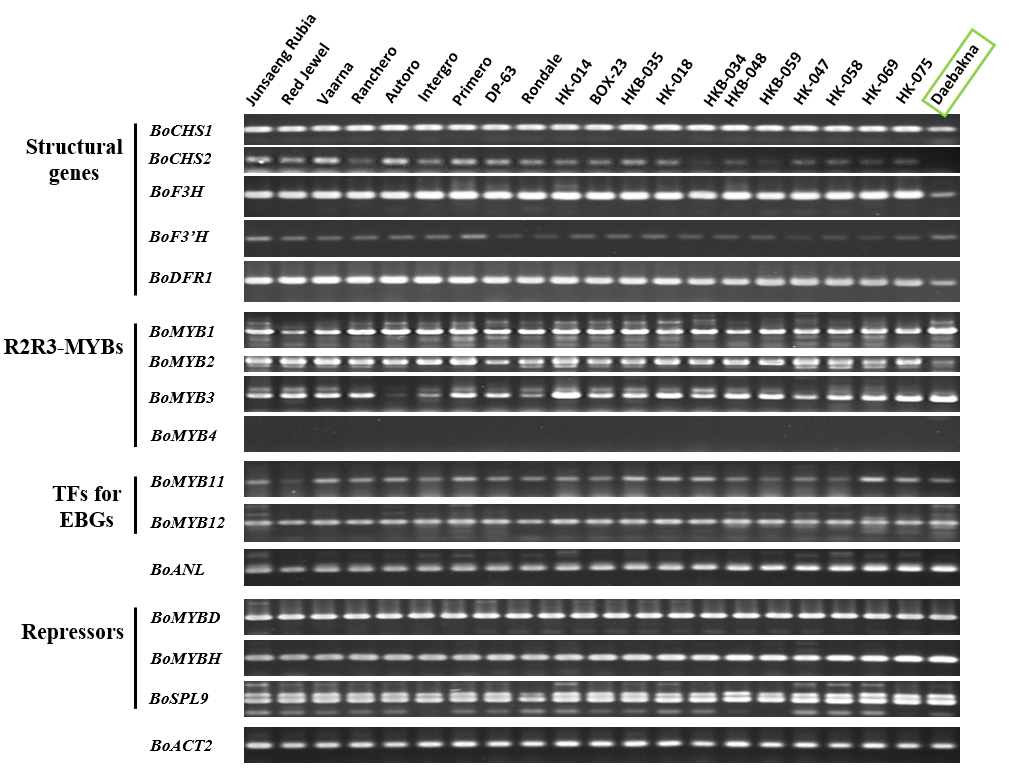


**Additional file 8: Figure S4.** Expression of genes associated with anthocyanin biosynthesis in various purple cabbages. Daebakna is a green cabbage used as a reference.
